# Protein signatures of seminal plasma from bulls with contrasting frozen-thawed sperm viability

**DOI:** 10.1038/s41598-020-71015-9

**Published:** 2020-09-04

**Authors:** Fabio P. Gomes, Robin Park, Arabela G. Viana, Carolina Fernandez-Costa, Einko Topper, Abdullah Kaya, Erdogan Memili, John R. Yates, Arlindo A. Moura

**Affiliations:** 1 The Scripps Research Institute, La Jolla, CA USA; 2 Federal University of Viçosa, Viçosa, MG Brazil; 3 Alta Genetics Inc., Madison, WI USA; 4 Selcuk University, Konya, Turkey; 5 Mississippi State University, Starkville, MS USA; 6 Federal University of Ceará, Fortaleza, CE Brazil

**Keywords:** Proteomics, Animal physiology

## Abstract

The present study investigated the seminal plasma proteome of Holstein bulls with low (LF; n = 6) and high (HF; n = 8) sperm freezability. The percentage of viable frozen-thawed sperm (%ViableSperm) determined by flow cytometry varied from -2.2 in LF to + 7.8 in HF bulls, as compared to the average %ViableSperm (54.7%) measured in an 860-sire population. Seminal proteins were analyzed by label free mass spectrometry, with the support of statistical and bioinformatics analyses. This approach identified 1,445 proteins, associated with protein folding, cell–cell adhesion, NADH dehydrogenase activity, ATP-binding, proteasome complex, among other processes. There were 338 seminal proteins differentially expressed (p < 0.05) in LF and HF bulls. Based on multivariate analysis, BSP5 and seminal ribonuclease defined the HF phenotype, while spermadhesin-1, gelsolin, tubulins, glyceraldehyde-3-phosphate dehydrogenase, calmodulin, ATP synthase, sperm equatorial segment protein 1, peroxiredoxin-5, secretoglobin family 1D and glucose-6-phosphate isomerase characterized the LF phenotype. Regression models indicated that %ViableSperm of bulls was related to seminal plasma peroxiredoxin-5, spermadhesin-1 and the spermadhesin-1 × BSP5 interaction (R^2^ = 0.84 and 0.79; p < 0.05). This report is the largest dataset of bovine seminal plasma proteins. Specific proteins of the non-cellular microenvironment of semen are potential markers of sperm cryotolerance.

## Introduction

Sperm cryopreservation, artificial insemination (AI) and in vitro fertilization followed by embryo transfer are among the most used assisted reproductive technologies (ARTs), allowing genetic selection of farm animals^1^, conservation of wild and endangered species^2^, successful pregnancies for infertile couples^3^ and preservation of fertility in cancer patients^4^. Successful implementation of ARTs depends on cost-effective and efficient cryopreservation of sperm cells. Cryopreservation protocols include the dilution of fresh semen with extender containing cryoprotectants, buffers and antibiotics, followed by freezing in liquid nitrogen. Freezing and thawing semen interferes with the structure of the sperm membranes, alters functions of membrane proteins and ion channels, causes premature capacitation and acrosome reaction and yields excessive reactive oxygen species^5^. Additionally, cryopreservation reduces both sperm metabolism and mitochondrial activity and alters sperm chromatin structure^6^. All these effects result in lower motility and fertilizing capacity of frozen-thawed sperm when compared to untreated cells.

Despite substantial improvements of protocols for cryopreservation of sperm and development of new extenders, it is currently accepted that 40 to 50% of sperm are incapable of proper fertilization after freezing and thawing^1^. Studies indicate that certain males with nearly identical sperm parameters measured in fresh ejaculates present contrasting sperm viability after cryopreservation^7^ and some bulls with high reproductive performance in natural mating have poor semen freezability^8^. Ejaculates have subpopulations of spermatozoa that differ in motility and fertilizing capacity, and these attributes are unique to each sire^9^. Aspects of sperm morphology and physiology are defined during spermatogenesis, epididymal maturation and during post-ejaculation events, influencing fertility of each male. Thus, it is not surprising that pronounced differences exist among individuals regarding sperm´s ability to survive the stress of cryopreservation.

In mammalian species, seminal plasma is mainly composed of secretions of the testes, epididymides and accessory sex glands. The fluid bathing sperm cells in semen contains proteins that participate in sperm metabolism and motility, membrane remodeling and function, protection against reactive oxygen species and immune actions, capacitation and acrosome reaction^10,11^. Many proteins of the seminal plasma bind to sperm, affecting the structure of membranes and sperm function. This is the case with Binder of Sperm proteins (BSPs), a major component of seminal plasma from ruminants and also conserved among several other mammalian species^12^. Studies indicate that BSPs protect ram spermatozoa subjected to cryopreservation^13^ and empirically relate to fertility of dairy bulls evaluated both in vitro^14^ and in vivo^15^. Treatment of sperm with diluted seminal plasma and sperm-rich fraction of seminal plasma is beneficial to frozen-thawed sperm from boars^16,17^ and cryotolerance of boar sperm can be estimated based on phosphorylation patterns of HSP70^18^, indicating that seminal components are important for viability of cryopreserved sperm. In addition to direct effects on sperm physiology, seminal proteins also appear to influence post-fertilization events as well. For instance, one of the members of the bovine BSP family, BSP1, affects cleavage and early in vitro growth of embryos^19^ and osteopontin has significant effects on cleavage rates and in vitro development of bovine^20^, swine^21^ and buffalo embryos^22^. Studies showing that ablation of accessory sex glands in hamsters negatively affects both embryo implantation and development^23,24^ reinforce the notion that paternal factors do play a major role in post-fertilization events. Components of semen also affect the physiology of the female reproductive tract, influencing reproduction success. In humans, seminal plasma stimulates the expression of pro-inflammatory genes in the cervix and vaginal epithelia and transcripts associated with cell proliferation, vascularization and angiogenesis in the endometrium^25,26^. Moreover, a seminal molecule, β-nerve growth factor, acts on the female reproductive tract to help trigger hormonal responses that cause ovulation in species of induced ovulation, such as rabbits and alpacas^27,28^. The attributes of seminal fluid molecules make them suitable targets of studies to elucidate factors controlling specific aspects of sperm function and male fertility. Thus, the present study was aimed at uncovering the protein signatures of seminal plasma from bulls with contrasting phenotypes associated with frozen-thawed sperm viability.

## Results

### The proteome of bovine seminal plasma

Using MudPIT, we identified 1,445 proteins in samples of bovine seminal plasma. BSP1 and spermadhesin-1 were the most abundant proteins of the bull seminal plasma, respectively, making 14.11 and 12.96% of all proteins presently identified. The three BSPs (BSP1, BS3 and BSP5) accounted for 22.57% and spermadhesins (spermadhesin-1 and spermadhesin Z13) made 14.08% of the bovine seminal plasma proteome deciphered by MuDPIT. Clusterin, osteopontin and metalloproteinase inhibitor 2 were the next most abundant proteins, making 4.53, 4.47 and 3.33% of the bull seminal plasma protein amount (Table 1). The 25 most abundant proteins accounted for nearly 70% of the seminal plasma proteome of the bulls (Supplemental Table 1).

**Table 1:** Functional attributes of proteins identified by multidimensional protein identification technology (MudPIT) in the bovine seminal plasma.

Accession #	Gene	Protein description	Abund. (%)*	Fold-change^§^	Functional attributes	Ref
P02784 **P81019** P04557		Seminal plasma protein PDC-109 (BSP1) **Seminal plasma protein BSP-30 kDa (BSP5)** Seminal plasma protein A3 (BSP3)	14.11 6.53 1.93	1.53^LF^ 1.22^HF^ 1.20^HF^	Bind do phospholipids of sperm membrane; sperm capacitation; sperm-oviduct interaction; in vitro fertilization and early embryo development. Protection of sperm against damage caused by cryopreservation	12, 13, 14, 15, 19, 40, 57, 76
P29392 P82292	SPADH1	Spermadhesin-1 Spermadhesin Z13	12.96 1.12	1.85^LF^ 1.43^HF^	Exhibit carbohydrate-binding activity and interact with phospholipids. Sperm capacitation; sperm interaction with the oviduct epithelium; sperm-egg binding; sperm membrane stability and motility. Anti-oxidative effects	46, 47, 48, 81, 82, 83, 84
P17697	CLU	Clusterin	4.53	1.42^LF^	Sperm maturation, lipid transport, sperm membrane remodeling; chaperone effects; inhibits cell lysis by complement-mediated mechanisms. Binds to damaged sperm	49, 50, 51, 53
P31096 P31098	SPP1	Osteopontin Osteopontin-K	2.63 1.84	1.58^LF^ 1.48^LF^	Involved in cell adhesion, tissue and extracellular remodeling, inflammation and immune-mediated events; Promotes acrosome reaction, in vitro fertilization and early embryo development	20, 21, 21, 55, 56, 57, 58, 59
P16368	TIMP2	Metalloproteinase inhibitor 2	3.33	1.50^LF^	Controls the activity of ADAMs (a disintegrin and metalloproteinase), proteins that function in cell adhesion, proteolysis of cell surface components and ECM. ADAMs participate in sperm-egg interactions	61, 62, 63
**P00669**	**SRN**	**Seminal ribonuclease**	2.89	2.58^HF^	Sperm capacitation; antioxidant function; catalytic activity; immunosuppression	77, 78, 78
*Q3SX14*	*GSN*	*Gelsolin*	1.02	4.63^LF^	Actin-binding molecule; maintains actin polymerization; regulated by calcium; triggers acrosome reaction	105
*Q2KJE5*	*GAPDHS*	*Glyceraldehyde-3-phosphate dehydrogenase, testis specific*	0.71	2.39^LF^	Glycolytic enzyme; essential for generation of ATP; play roles in sperm motility and male fertility; binds to sperm fibrous sheath	99, 100
*Q3MHM5* *Q3ZBU7* *Q2KJD0* *Q2KJD0*	*TUBB4B* *TUBB4A* *TUBB5* *TUBB2B*	*Tubulin beta-4B chain* *Tubulin beta-4A* *Tubulin beta-5 chain* *Tubulin beta-2B chain*	1.31 1.18 0.79 0.57	2.37^LF^ 2.38^LF^ 2.43^LF^ 2.39^LF^	Major components of sperm microtubules; binds to GTP; involved in the mechanism of sperm motility	93, 94
*P00829*	*ATP5F1B*	*ATP synthase subunit beta*	0.38	2.68^LF^	Sperm ATP production; may play a role in germ cell differentiation	95, 96
*Q9BGI1*	*PRDX5*	*Peroxiredoxin-5*	0.32	2.68^LF^	An antioxidant effect; increased expression during immune response and inflammation; modulators of redox signaling; overexpressed in certain types of carcinogenic processes	85, 86, 87, 88, 89, 90, 91, 92
*P62157*	*CALM*	*Calmodulin*	0.29	2.89^LF^	Binds to sperm, controls Ca2+-mediated events; participates in sperm capacitation and acrosome reaction; sperm motility	103, 104
*Q32KL7*	*SPESP1*	*Sperm equatorial segment protein 1*	0.25	4.05^LF^	Mediates sperm-egg fusion and fertilization	101, 102
*A0JNP2*	*SCGB1D*	*Secretoglobin family 1D member*	0.23	4.27^LF^	Steroid binding protein; anti-inflammatory effect; inactivates phospholipase A2; decreases proinflammatory cytokine production; alters phagocyte function. May regulate sperm antigenicity	106, 107
*Q3ZBD7*	*GPI*	*Glucose-6-phosphate isomerase*	0.22	2.50^LF^	Converts glucose-6-phosphate to fructose-6-phosphate; important for glycolysis and ATP yield for sperm motility; regulates tumor cell growth; prevents apoptosis and oxidative stress-induced cellular events	97, 98

The list includes proteins with the highest relative quantity in seminal plasma and those proteins identified with the 15 highest VIP scores, as shown in Fig. 2. Highlighted proteins are those that made significant contributions for the definition of the high (bold) and low (italics) sperm freezability phenotypes.

*Refers to how much the abundance of a specific protein represents, in percentage, of the total abundance of all proteins detected in the study.

**Figure 1:**
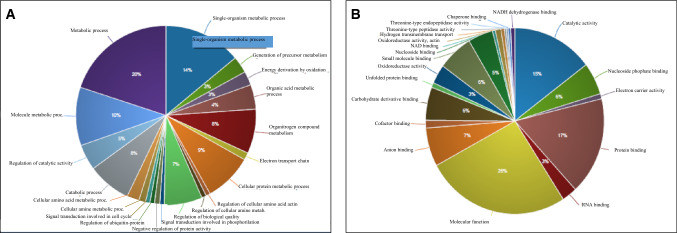
Gene ontology terms of proteins from bovine seminal plasma associated with biological processes **(A)** and molecular function **(B)**. Protein data and identification were analyzed by the Integrated Proteomics Pipeline version 6.5.4. (IP2, https://www.integratedproteomics.com/) and based on UniprotKB database.

Based on gene ontology terms defined in the IP2 platform, predominant biological processes associated with the bull seminal proteins were metabolic process, single-organelle metabolic process, small molecule metabolic process, cellular protein metabolic process, organonitrogen compound metabolic process, regulation of catabolic process and catalytic activity, among others (Fig. 1A). Molecular function of those seminal plasma proteins mainly related to catalytic activity, protein binding, anion binding, nucleoside phosphate binding, and electron carrier activity, among others (Fig. 1B).

We uploaded 1,445 Uniprot accession codes through the DAVID platform which matched to 1,407 genes, distributed in 186 clusters. Each of these functional clusters included substantial numbers of biological mechanisms, pathways, molecules and cell structures (Supplemental Table 2). Functional annotation of the 15 highest cluster enrichment scores based on gene ontology and KEGG pathway database included biological processes such as protein folding and cell–cell adhesion, molecular functions linked to NADH dehydrogenase activity, unfolded protein binding and cadherin and ATP-binding, and cellular components related to lysosome, cell–cell adherens junction and proteasome accessory complex, among others (Table 2).

**Table 2:** Functional annotations of the clusters with the 15 highest enrichment scores, associated with bovine seminal plasma proteins.

Functional annotation clusters	Enrichment scores	Number of genes
**Biological processes**		
Protein folding	17.48	37
Cell–cell adhesion	15.03	31
Tricarboxylic acid cycle	13.12	21
Glycolytic process	9.86	15
Negative regulation of endopeptidase activity	8.61	32
ATP hydrolysis coupled proton transport	7.52	15
ATP synthesis coupled proton transport	7.01	14
**Molecular functions**		
NADH dehydrogenase (ubiquinone) activity	33.93	22
Unfolded protein binding	17.48	26
Cadherin binding involved in cell–cell adhesion	15.03	34
ATP-binding	14.25	149
Serine-type endopeptidase inhibitor activity	8.61	25
Threonine-type endopeptidase activity	8.52	14
Proton-transporting ATPase activity, rotational mechanism	7.52	14
Hydrogen ion transmembrane transporter activity	7.01	9
GTP binding	6.51	72
GTPase activity	6.51	46
GDP binding	6.51	16
**Cellular components**		
Mitochondrial respiratory chain complex I	33.93	34
Lysossome	17.21	46
Cell–cell adherens junction	15.03	36
Proteasome accessory complex	10.4	13
Chromaffin granule	8.61	9
Proteasome core complex	8.52	15
Cytoplasmic mRNA processing body	8.52	4
Mitochondrial proton-transporting ATP synthase complex	7.01	15
**Pathways (based on KEGG pathway database)**		
Oxidative phosphorylation	33.93	83
Lysossome	17.21	42
Citrate cycle (TCA cycle)	13.12	22
Oxocarboxylic acid metabolism	13.12	10
Proteasome	10.4	31
Glycolysis/gluconeogenesis	9.86	28
Proteasome	8.52	31
Collecting duct acid secretion	7.52	12

Clusters were based on Gene Ontology and KEGG pathways. The current list is part of the 186 clusters identified by DAVID platform, as defined in Supplemental Table 2.

### Proteins differentially expressed in the seminal plasma of bulls with high and low sperm freezability

**Figure 2:**
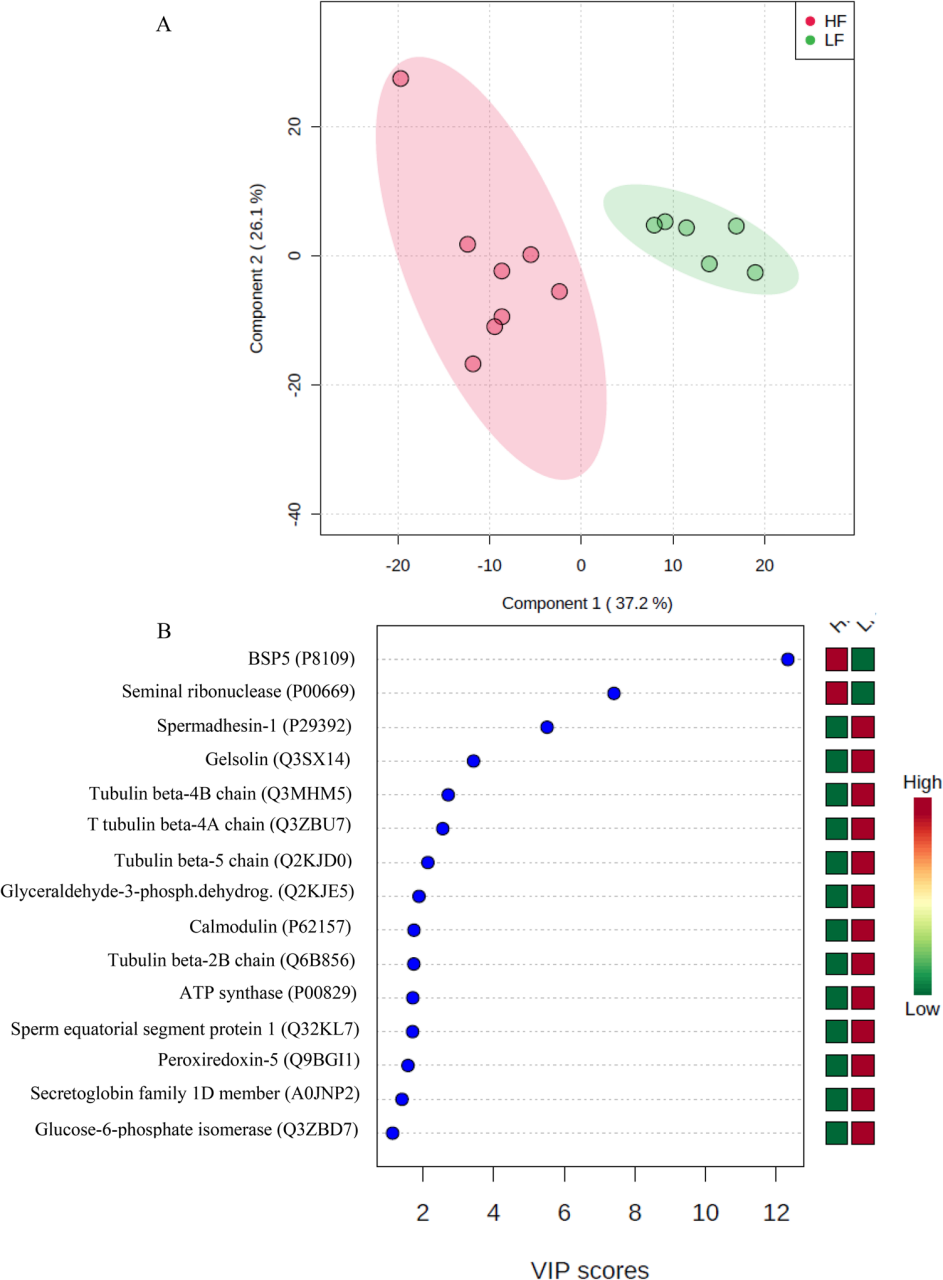
Partial least square discriminant analysis (PLS-DA) of protein abundances in the seminal plasma of dairy bulls with low (LF) and high (HF) sperm freezability phenotypes. Explained variances of components are shown in brackets **(A)**. Variable Importance in Projection (VIP) scores associated with seminal proteins, as identified by PLS-DA **(B)**. Colored boxes on the right indicate the relative abundances of proteins in each phenotype group.

Label free quantitative analysis showed that there were 338 proteins with different abundances (p < 0.05) in the group of bulls with low and high semen freezability. A clear separation of seminal plasma proteomes from LF and HF bulls was also detected by partial least square discriminant analysis (Fig. 2A). Moreover, multivariate analysis identified 15 proteins with the highest VIP scores, which indicates their significant contributions to the definition of sperm freezability phenotypes. Among those 15 proteins, seminal plasma BSP-30 kDa (BSP5) and seminal ribonuclease contributed to characterization of the high freezability phenotype. On the other hand, spermadhesin-1, gelsolin, tubulin beta-4B chain, tubulin beta-4A chain, tubulin beta-5 chain, glyceraldehyde-3-phosphate dehydrogenase, calmodulin, tubulin beta-2B chain, ATP synthase, sperm equatorial segment protein 1, peroxiredoxin-5, secretoglobin family 1D member and glucose-6-phosphate isomerase formed the group of proteins characterizing the low freezability phenotype (Fig. 2B).

Regression analyses using abundances of proteins with the 15 highest VIP scores as independent variables generated equations for prediction of sperm freezability scores of bulls. According to one model, these scores were related to the amount of spermadhesin-1, peroxiredoxin-5 and BSP5 in seminal plasma (R^2^ = 0.80, p < 0.05; Table 3). In another regression equation, parameters explaning the variation in sperm freezability scores were spermadhesin-1, peroxiredoxin-5 and the spermadhesin-1 × BSP5 interaction (R^2^ = 0.79, p < 0.05; Table 3; Suppemental Table 3). There were significant (p < 0.05) correlations among several of those 15 high-VIP score proteins. For instance, the amount of PRDX5 correlated with that of ATP synthase (r = 0.59), which in turn showed correlations with glyceraldehyde-3-phosphate dehydrogenase (r = 0.95), sperm equatorial segment protein 1 (r = 0.95) and glucose-6-phosphate isomarase (r = 0.89) in the seminal plasma. Quantity of GPI in seminal plasma had association with the amount of GAPDHS (r = 0.87), sperm equatorial protein 1 (r = 0.83) and secretaglobin (r = 0.72). GAPDHS levels were correlated with those of spermadhesin-1 (r = 0.74) and calmodulin (r = 0.56), which were linked to the amounts of secretoglobin (r = 0.75) and GPI (r = 0.70) as well. Quantities of spermadhesin-1 were correlated with those of gelsolin (r = 0.64) in the seminal plasma of bulls (Supplemental Table 4).

**Table 3:** Regression models showing the percent deviation of frozen-thawed sperm viability (Y) as a function of seminal plasma proteins from dairy bulls.

	Pr >|t|	R^2^	Pr > F
**Model 1**			
Y = 212.062253 – a.(LogPRDX5) + b.(BSP5) – c.(LogSPADH1)		0.80	0.0008
a = 8.614414 b = 2.2628731E-9 c = 15.033786	0.0164 0.0207 0.0087		
**Model 2**			
Y = 221.301878 – a.(LogSPADH1) – b.(LogPRDX5) + c.(Log SPADH1xLogBSP5)		0.79	0.001
a = 30.270225 b = 7.814084 c = 1.515310	0.0068 0.0333 0.0288		

Analyses of variation, parameter estimates and fit diagnostics are available in Supplemental Table 3. SPADH1: spermadhesin-1 (accession # P29392); PRDX5: peroxiredoxin 5 (accession # Q9BGI1); BSP5: seminal plasma protein BSP-30 kDa (accession # P81019).

*PRDX5* peroxiredoxin, *BSP5* seminal plasma protein BSP-30 kDa, *SPADH1* spermadhesin-1.

**Figure 3:**
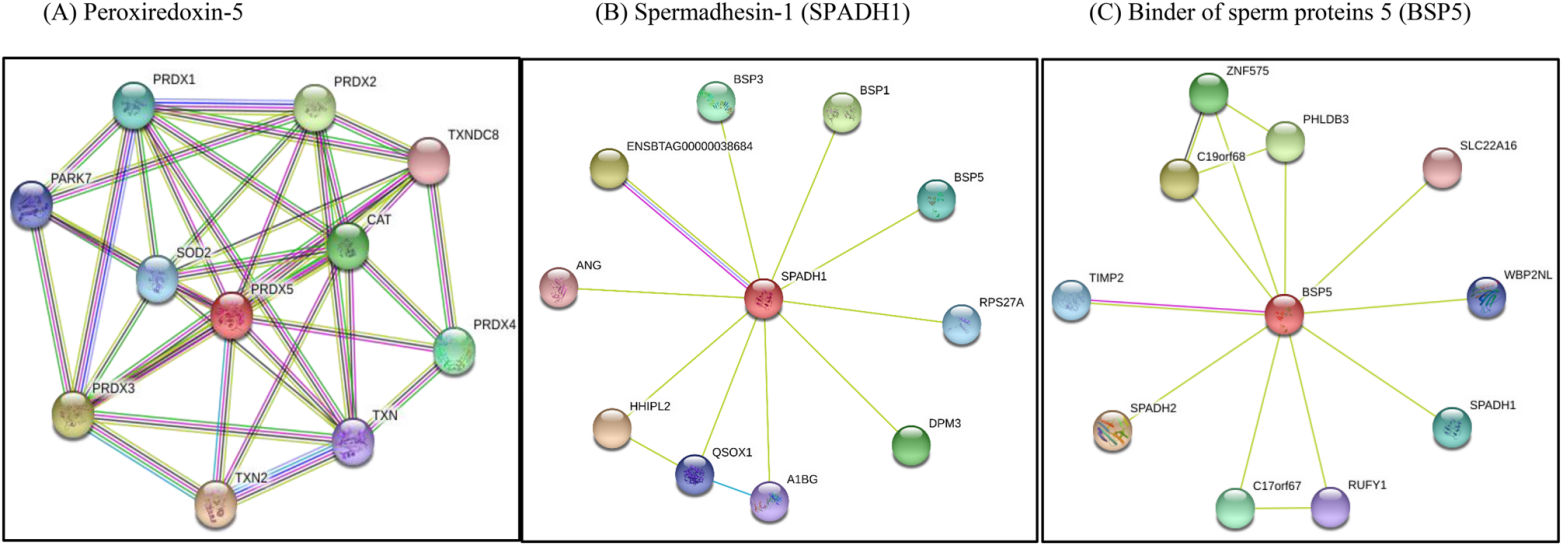
In silico networks of seminal plasma spermadhesin-1 (SPADH1), peroxiredoxin 5 (Prdx5) and Binder of sperm protein 5 (BSP5), based on String platform (https://string-db). SPADH1, PRDX5 and BSP5 were significantly related to freezability scores of dairy bulls, according to the regression model presented in Table 2.

Based on in silico analysis, peroxiredoxin-5 shows potential interactions with peroxiredoxin-1, 2, 3 and 4, three types of thioredoxin, catalasem, superoxide dismutase and protein/nucleic acid deglycase DJ-1 (Fig. 3A). Spermadhesin-1, in turn, has links with BSP5 and other two members of the BSP protein family (BSP1 and BSP3), ubiquitin-40S ribosomal protein S27a, dolichol-phosphate mannosyltransferase subunit 3; alpha-1B-glycoprotein and quiescin Q6 sulfhydryl oxidase 1 (Fig. 3B). BSP5 interacts with tissue inhibitor of metalloproteinase-2, spermadhesin Z13 (in addition to spermadhesin-1), a solute carrier family 22 member 16 and a postacrosomal sheath WW domain-binding protein (Fig. 3C).

## Discussion

The analysis of bovine seminal plasma conducted in the present study relied on multidimensional protein identification technology and tools of bioinformatics. This approach allowed the identification of 1,445 proteins, which currently represents the largest dataset of proteins in seminal plasma from bulls or any ruminant species. In a previous study, we identified 1,159 proteins in seminal plasma of Holstein bulls using a conventional bottom-up proteomics approach^15^. While our current work brings a valuable contribution to the field of male reproductive biology, it probably does not represent the entire proteome of the bovine seminal plasma. Advanced studies estimate that human seminal plasma contains up to 10,000 proteins^29^, and we believe that the bovine seminal plasma likely has a comparable number of proteins. Binder of sperm proteins and spermadhesins were expressed in high levels in the bovine seminal plasma and it is possible that these dominant proteins suppressed the signal of other proteins with lower abundance. Since identifying low abundance proteins is a common drawback of conventional bottom-up proteomics, we utilized the MudPIT approach to analyze our samples in order to increase the proteome coverage^30^. MudPIT is based on multidimensional separation where two stationary phases are used online to increase the separation efficiency of the chromatographic method, which in turn, improves mass spectrometry detection of the peptides. In fact, our strategy improved the overall view of the bovine seminal plasma proteome but it is still far from the results described in humans. Thus, new analytical and separation methods are needed to decipher the complete proteomic atlas of the bovine seminal plasma.

As expected, BSPs (BSP1, BSP5 and BSP3) and spermadhesins were the most abundant proteins detected in the present study, along with other typical components of bovine seminal plasma, such as clusterin, osteopontin and metalloproteinase inhibitor 2^7,15,31–33^. In addition to the bovine, BSPs and their isoforms are present in the seminal plasma of other ruminants such as buffalos^34^, rams^35,36^ and goats^37^. Horses^38^, rabbits^39^ and humans^12^ also express BSPs in semen, but in lower amounts than ruminants. Most studies about BSPs have been conducted in bulls and it is believed that BSPs interact with sperm at the moment of ejaculation, participate in capacitation^12^, mediate the interaction between spermatozoa and oviduct epithelia^40^ and affect fertilization as well^19^.

Spermadhesins were the second most abundant protein family detected in the bull seminal fluid, appearing in our list as spermadhesin-1 and spermadhesin Z13. Like BSPs, spermadhesins are present not only in bulls^7,33,41^, but also in rams^36^, buffalos^42^, goats^43^, boars^44^ and peccaries^45^, among other species. Spermadhesins exhibit carbohydrate-binding activity and interact with certain types of phospholipids^46^. Roles played by spermadhesins vary among species but studies conducted in boars indicate that they affect sperm capacitation, sperm-oviduct interaction, sperm membrane stability and sperm-egg binding^47,48^. Clusterin was the third most abundant protein of the bovine seminal plasma, affecting sperm function in many ways. Clusterin play roles in sperm maturation, lipid transport and membrane remodeling, acts as chaperone^49,50^ and inhibits cell lysis by complement-mediated mechanisms, mitigating female immune reactions to male factors^51^. Seminal plasma clusterin is inversely related to the population of morphologically normal sperm in bulls^52^ and seems to selectively bind sperm with morphologic defects^53^. We have reported that clusterin is consistently expressed in the seminal plasma of several species, such as bulls^7,14,33^, rams^36^, boars^44^, peccaries^45^ and dogs^54^, among others. It seems therefore that males have a selected group of components which we define as “seminal plasma sperm-protecting molecules”. Clusterin belongs to this group, as well as peroxiredoxins, albumin, catalase, gluthathione peroxidase, glutathione S-transferase, tioredoxin peroxidase, superoxide dismutase, catalase and lactoferrin, among others. They all contribute to protection of sperm against oxidative stress, protein precipitation and immune reactions of the female reproductive tract.

Osteopontin (OPN) and metalloproteinase inhibitor 2 were other typical proteins of the bull seminal fluid, although expressed in much less quantity than BSPs and spermadhesins. OPN is involved in cell adhesion, tissue and extracellular remodeling, inflammation and immune-mediated events^55,56^. OPN binds to sperm integrin and CD44 receptors^57,58^ and promotes acrosome reaction, fertilization and early embryo development in the bovine^20,59^ and swine^21^. Seminal plasma OPN has been regarded as a marker of fertility in dairy bulls^41,60^. Metalloproteinase inhibitor 2, in turn, controls the activity of ADAMs (a disintegrin and metalloproteinase), proteins that modulate cell adhesion and act during proteolysis of extracellular matrix and cell surface components^61^. ADAMs participate in sperm-egg interaction and seminal plasma tissue inhibitor of metalloproteinase 2 has been associated with bull fertility^62^ and with the degree of sperm DNA fragmentation in men^63^. Functional attributes of the major bovine seminal plasma proteins as well as those proteins defining the sperm freezability phenotypes of the bulls are summarized in Table 1.

Major biological processes (metabolic process, small molecule metabolic process, regulation of catalytic activity and catabolic process) and molecular functions (catalytic activity, nucleoside phosphate binding, electron carrier activity and protein binding) of the bull seminal proteins detected in the present study appear as common events of mammalian sperm physiology. As expected, functional clusters associated with 1,407 genes identified in the DAVID platform were extremely diverse. Yield of viable sperm by an individual and successful fertilization depend on mitosis, meiosis and differentiation of germ cells in the seminiferous tubules, Sertoli and Leydig cell functionality, epididymal maturation and post-ejaculation events, such as capacitation and acrosome reaction. As seminal plasma molecules are usually in close contact with sperm and affect their structure and metabolism starting in the testes, it is not surprising that terms of functional clusters described in our study are so diverse.

Functional clusters with the 15 highest enrichment scores related to protein folding, cell–cell interaction, energy yield, cell metabolism, protease activity and mitochondrial reactions. Our analysis of seminal plasma indicates 83 genes related to oxidative phosphorylation and 28 to glycolysis, which consist of metabolic pathways used by spermatozoa to generate ATP^64^. Also, lysosome activity was linked to 42 genes, including proteases (such as cathepsins), glycosidades, sulfatases, prosaposin, among others. Previous studies indicate that cathepsin D, an epididymal enzyme, is involved in membrane protein remodeling and is empirically related to fertility scores of dairy bulls^65^. Prosaposin is a glycoprotein found in reproductive tract secretions of several species, including humans^66^. Prosaposin has important functional attributes in male reproduction, as knock-out mouse for this protein has deficient spermatogenesis^67^. Also, prosaposin apparently improves sperm-oocyte interaction and fertilization in bulls and humans^68,69^. The acrosome is formed during spermiogenesis and it consists of a vesicle capping the sperm head, containing proteolytic enzymes that are released prior to fertilization^70^. Because of its nature, types of enzymes and physiology, the acrosome reaction is understood to have a lysosome-like mechanism and our study indicates that a significant number of proteins of the seminal plasma potentially participate in that event.

Pathways with high enrichment scores were also associated with the proteasome, with a pronounced number of seminal plasma components. The proteasome is a protein complex designed to degrade proteins specifically marked by ubiquitination. This conserved cellular proteolytic activity takes place during several biological events, such as cell cycle growth, apoptosis, cell signaling, metabolic reactions and immune-related processes^71^. Ubiquitin–proteasome components are present in the somatic and germ cells of the testis and play a role in the replacement of histones for protamines at the final stages of spermatogenesis^72^. Proteasomes can also be secreted and they are found in human seminal plasma^73^ and epididymal fluid of bulls^74^. Sperm are ubiquitinated during their transit through the epididymis^72^ and it has been postulated that this ubiquitination labels defective sperm, signaling for their degradation mediated by proteasome^72,74^. With the description of several components of the proteasome in the bull seminal plasma, there is an expectation that proteolytic-mediated removal of defective sperm also happens after ejaculation, in the female reproductive tract. Because IFNγ was detected in the seminal plasma samples of the present study, we hypothesize that immunoproteasomes are formed in the male reproductive tract and/or after ejaculation. However, more research is needed to determine the precise roles of these immunoproteasomes in the fertilization process.

Through this innovative study, we report a selected group of proteins with significant associations with the ability of spermatozoa to survive cryopreservation. Considerable differences exist among bulls as regard to how gametes survive freezing and thawing^1,7,8^. Despite the importance of gamete survival to assisted reproduction, there is no definitive explanation of why sperm from some animals “freeze” better than from others. Because semen from dairy bulls are extensively used for commercial AI, these animals are excellent models for studies designed to understand the molecular basis of sperm freezability and male fertility. Also, dairy bulls have been subjected to extensive selection for the last decades so animals with poor semen quality are culled, and those currently used for artificial insemination are routinely evaluated. This workflow of semen semen evaluation and cryopreservation in the AI industry generates a remarkable database about sperm parameters and fertility of bulls, leading to reliable characterization of phenotypes of the animals^5^.

We presently show that bulls with contrasting sperm freezability phenotypes have distinct seminal plasma protein signatures. Freezability scores used in the current research were very reliable because they were based on evaluations of at least 50 semen samples per animal and compared to data from a large population of sires. However, the proteome analyses were carried out in a single seminal plasma sample per bull. Conditions in which bulls of AI centers are raised are well controlled, from nutrition, health and management to frequency of semen collections. This minimizes inter-ejaculate variations in semen composition. However, despite all standard protocols for raising the animals at AI facilities, between-ejaculate variations do occur, of course. Even when all management conditions are perfectly controlled, subtle physiological changes may happen from day to day in any living organism. Thus, associations presently reported between viability of frozen-thawed sperm and seminal plasma proteins need to be validated with different group of animals. Effects of selected proteins on sperm cryotolerance and function could also be tested in in vitro assays.

Based on VIP scores, proper sperm survival to cryopreservation was mostly defined by BSP5 and seminal ribonuclease. BSPs interact with the bovine sperm when epididymal sperm come in contact with secretions of accessory sex glands and participate in capacitation, sperm-oviduct binding and fertilization, as previously mentioned. In rams, seminal plasma from individuals with good sperm freezability improve motility when mixed with frozen-thawed sperm from animals with poor frezability^75^. A positive cause and effect relationship exists between BSP1 and BSP5 and both motility and membrane integrity of frozen-thawed sperm of rams^13^, indicating that BSPs protect spermatozoa against the damages of cryopreservation. In the bovine, sperm become damaged when they are exposed to high concentrations of BSPs and when they are exposed to BSPs in vitro for long periods of time^12^. Such deleterious effect occurs because excess of BSP exposure leads to removal of too much cholesterol and phospholipids from the sperm membrane. In physiological conditions, contact of sperm with seminal plasma after ejaculation is transient and it happens while semen is diluted in the secretions of the female reproductive tract. In vitro, however, this dilution of semen does not seem to happen and thus, BSP effects may be different in vivo and in vitro. Nevertheless, positive associations exist between seminal BSPs and fertility indexes of dairy bulls^14,15,41^ but it is also true that too much BSP in seminal fluid is detrimental to sire fertility^41^. Also, BSP coating to bovine sperm^57^ contributes to stability of sperm membrane structure^76^, supporting the notion that BSPs can protect cryopreserved sperm. Seminal ribonuclease (SRN) is another protein associated with the high freezability phenotype. SRN participates in sperm capacitation and has catalytic activity, acting as antioxidant and suppressor of immune reactions^77^. These last two attributes make SRN a “seminal plasma sperm-protecting molecule”. SRN positively relates to motility of buffalo spermatozoa^78^ while a ribonuclease 4 isoform was inversely associated with cryotolerance of ram sperm^79^. Studies clearly indicate that both BSPs and SRN contribute to proper sperm function but more investigation is needed to characterize the specific mechanisms by which such proteins affect sperm resistance to cryopreservation.

Seminal proteins defining the low sperm freezability phenotype were identified as spermadhesin-1, gelsolin, tubulins, glyceraldehyde-3-phosphate dehydrogenase, calmodulin, ATP synthase, sperm equatorial segment protein 1, peroxiredoxin-5, secretoglobin and glucose-6-phosphate isomerase. Regression analysis indicated that sperm freezability scores of bulls were inversely related to spermadhesin-1 and peroxiredoxin-5. Spermadhesin-1 is also known as acidic seminal fluid protein (aSFP) and secreted by accessory sex glands^31^ and cauda epididymis of bulls^80^. The reason why spermadhesin-1/aSFP has an inverse association with frozen-thawed sperm survival is not well understood. aSFP has protective actions against oxidative stress in semen^81,82^ but effects of aSFP on sperm motility and mitochondrial activity can be either beneficial, when aSFP is at average levels, or detrimental, when at high concentrations^81^. Spermadhesins also share amino acid homology with seminal plasma motility inhibitor, a molecule that interferes with ATPase and reduces sperm motility in humans and boars^83^. However, studies demonstrate that phospholipid binding attributes of AWN, a porcine spermadhesin, promote stability of the sperm membrane^84^. It seems possible, then, that spermadhesins can have either beneficial or damaging effects on sperm, depending on the concentration. Moreover, in silico analyses indicate that spermadhesin-1 interacts with BSPs and other components of the male reproductive tract such as ubiquitin, alpha-1B-glycoprotein, quiescin Q6 sulfhydryl oxidase 1 and angiogenin-1, suggesting that in addition to their isolated effects, the form by which proteins interact in the seminal plasma environment also affects sperm physiology. In accordance with this analysis, one of the regression models presented in the current study indicates that the spermadhesin-1 × BSP5 interaction was significant to determine the variation in sperm freezability scores of bulls.

Peroxiredoxin-5 was another seminal plasma protein defining the low sperm freezability phenotype of the animals and significantly included in the regression models. Peroxiredoxins (PRDX) are thiol-dependent peroxidases capable of protecting cells from reactive oxygen species (ROS) and other damaging molecules^85^. PRDXs induce redox signaling for macrophage actions and are released in response to high yield of peroxides during inflammation processes^85,86^. Certain types of PRDX, such as PRDX5, relate to progression of carcinogenic tumors and are overexpressed in cases of endometrial and ovarian cancer. These associations are certainly linked to intense oxidative stress-related events that characterize cancer pathobiology^87^. As well known, oxidative stress is harmful to sperm’s fertilizing capacity^88^ and seminal plasma PRDX expression is one of the strategies for sperm protection, as discussed above. Peroxiredoxins are present in human sperm and seminal plasma^88^ and both PRDX1 and PRDX6 are altered in sperm of men with seminal oxidative stress and pathological conditions such as varicocele^89,90^. PRDX4 and PRDX5 have been described in the testis^91^ and cauda epididymal sperm^92^ of bulls, respectively. However, no information exists about the expression of PRDXs in the bovine seminal plasma and ejaculated sperm and what controls their synthesis either. The reason PRDX5 had an inverse association with viability of frozen-thawed sperm in the present study is not known at this point. High levels of PRDXs5 in seminal fluid could possibly happen as the response of some animals to increased release of ROS in the epididymis and/or semen. This would significantly damage sperm membrane integrity, decreasing sperm’s freezability. Based on in silico analysis, PRDX5 interacts with other types of peroxiredoxins and enzymes involved in the control of redox reactions, such as thioredoxins, catalase and superoxide dismutase. Moreover, PRDX5 networks with ATP synthase, which in turn, connects with tubulins, glucose-6-phosphate isomerase (GPI) and glyceraldehyde-3-phosphate dehydrogenase (GADPHS; data not shown), proteins that also contributed to definition of the low sperm freezability phenotype of bulls. In agreement with silico assays, the quantity of PRDX5 in seminal plasma correlated with that of ATP synthase, which in turn was related to the amount of spermadhesin-1, tubulins, GAPDHS, sperm equatorial protein 1 and GPI. Tubulins are constituents of the sperm motility apparatus^93,94^, while ATP synthase^95,96^, GPI^97,98^ and GADPHS^99,100^ take part in the process of energy production for sperm cells. Sperm equatorial protein 1 participates in sperm-egg binding and in the process of fertilization^101,102^. As mentioned above, several protein interactions possibly occur in the non-cellular microenvironment of semen, affecting crucial aspects of sperm function. In fact, we detected intricate associations among seminal proteins involved in sperm-egg fusion and fertilization (sperm equatorial segment protein 1, SPADH1); sperm protection (SPADH1 and PRDX5); energy metabolism and sperm motility (tubulins, ATP synthase, GPI, GAPDHS); Ca2+-mediated events, capacitation and acrosome reaction (calmodulin^103,104^, gelsolin^105^); steroid binding and inflammation-related events (secretoglobin^106,107^) and actin polymerization (gelsolin^105^).

In summary, we report the use of multidimensional protein identification technology, mass spectrometry and bioinformatics to study the bovine seminal plasma proteome. This approach allowed the identification of 1,445 proteins, to date the largest database of seminal proteins from bulls and any ruminant species. The present research broadens our view about the complexity and functional attributes of the milieu in which sperm cells subsist after leaving the male reproductive tract. Associations between selected seminal proteins (peroxiredoxin 5, spermadhesin-1 and BSP5) and well-defined phenotypes of sperm freezability are encouraging and set the basis for future indentification of potential markers of male fertility.

## Materials and methods

### Experimental design

In the present study, we used the well-established multidimensional protein identification technology (MudPIT)^108^, tools of bioinformatics and statistical analysis to decipher the proteome of seminal plasma in dairy bulls with low and high sperm freezability. Phenotypes of sperm freezability were based on frozen-thawed sperm viability measured by flow cytometry and compared with the viability of samples in a large population of sires maintained at an artificial insemination company in the USA (Alta Genetics, Inc.).

### Semen collection and seminal plasma samples

Seminal plasma was obtained by centrifugation of semen samples from 14 healthy Holstein bulls. There was one semen sample per bull. As previously described^15^, semen was collected using artificial vagina and a protease inhibitor was immediately added to all samples. Then, semen was centrifuged at 800 × *g* for 15 min (4 °C) and the supernatant (seminal plasma) was transferred into sterile tubes. Seminal plasma was centrifuged again at 10,000 × *g* for 30 min (4 °C) and the resulting fluid was pipetted into new tubes, frozen in liquid nitrogen and stored at − 80 °C until further use. Seminal plasma samples used in the current research were obtained from a commercial source, collected according to established and approved protocols (Alta Genetics, Inc.).

### Determination of semen freezability

Semen samples and phenotypic characterization of the Holstein dairy sires were provided by Alta Genetics Inc. (Watertown, WI, USA), one of the largest breeding companies of the world, with semen collection and processing laboratories in six countries that provide frozen semen to over 90 countries.

Freezability scores of the bulls were based on viability of frozen-thawed sperm (%ViableSperm). In summary, fresh ejaculates were extended with commercial, one-step Egg-Yolk-Tris based extender, and then frozen using standard protocols at Alta Genetics Inc.^109^. Post-thaw sperm viability was assessed by flow cytometry using fluorescent stain SYBR-14 with propidium iodide (SYBR-14/PI, Live/Dead Sperm Viability Kit L-7011; Thermo Fisher Scientific, USA), as previously described^110^. Membrane integrity of 10,000 frozen-thawed sperm cells from each ejaculate allowed the calculation of the percentage of viable frozen-thawed sperm. Then a unique freezability phenotype was generated to characterize post-thaw sperm viability for each bull. For the current research, we used post-thaw viability data generated over 8 years (between 2008 and 2016). The database included 100,448 ejaculates from 860 Holstein bulls, each collected at least 20 different times within approximately 3-month periods. The average post-thaw sperm viability for individual bulls was calculated, and then bulls were ranked relative to %ViableSperm of the population. Post-thaw viability of all bulls ranged from 33.03 to 67.3%, reaching an average of 54.7 ± 5.4%. For the present study, %ViableSperm of bulls with low sperm freezability (LF; n = 6) ranged from − 5.8 to + 0.3 and %ViableSperm of bulls with high sperm freezability (HF; n = 8) varied from + 4.6 to + 11.5 relative to the average %ViableSperm of the population. There were at least 50 frozen-thawed semen samples evaluated per animal (Table 4). The ejaculate from which seminal plasma was used for the proteomic analysis (described below) was not analyzed for calculation of freezability scores of the bulls. This ejaculate was obtained in 2016, right after the analyses of the database for estimation of the LF and HF phenotypes. All bulls were kept under the same housing conditions and nutritional management during semen collections at Alta Genetics facilities.

**Table 4:** Viability of frozen-thawed sperm from dairy bulls, as determined by flow cytometry.

Bull #	Number of frozen-thawed semen samples	Frozen-thaw sperm viability (%)		Phenotype
		Average	Difference from population average (freezability score)	
1	79	48.9	− 5.8	LF
2	107	49.2	− 5.5	LF
3	194	52.7	− 2.0	LF
4	264	54.8	− 0.1	LF
5	71	54.9	0.2	LF
6	229	55.0	0.3	LF
Avg ± SE	157 ± 30.7	52.6 ± 1.1	− 2.2 ± 1.1	
7	138	59.3	4.6	HF
8	81	59.9	5.2	HF
9	113	61.9	7.3	HF
10	153	62.3	7.7	HF
11	207	62.8	8.1	HF
12	266	63.0	8.3	HF
13	50	64.4	9.7	HF
14	116	66.2	11.5	HF
Avg ± SE	141 ± 22.9	64.5 ± 0.5	7.8 ± 0.7*	

Bulls had low (LF) or high (HF) sperm freezability phenotypes based on the percentage deviation of the average post-thaw viability of sperm measured in the population of all bulls. Population average of frozen-thaw sperm viability was 54.7%.

*p < 0.001.

### Sample preparation for mass spectrometry

Seminal plasma samples (30 µl/tube) were thawed at room temperature and subjected to protein precipitation. Briefly, cold acetone (– 20 °C) was added to seminal plasma (four times the sample volume), vortexed and placed at – 20 °C overnight. Afterwards, this mix (sample + acetone) was subjected to three sequential centrifugations at 14,000 × *g* (at 4 °C for 10 min.), always discarding the supernatant and resuspending the protein pellet. Following the last spin, the pellet was left on the bench for air dry and then frozen at – 80 °C. An aliquot of the precipitated protein samples was used to determine the protein concentration using the Pierce BCA Protein Assay Kit (Thermo Scientific, USA).

Precipitated proteins (50 µg) were dissolved in 8 M urea, 100 mM Tris pH 8.5 (60 ul), mixed with 0.3 ul 1 M TCEP—tris(2-carboxyethyl) phosphine and incubated at room temperature (RT) for 20 min. Then, 6.6 ul 500 mM 2-chloro-acetamide was added to the solution and incubated for for 15 min in the dark (at RT). Samples diluted with 100 mM Tris pH 8.5 were mixed with 100 mM CaCl 2 to a final conc. of 1 mM. Then, trypsin (0.5 ug/ul; Promega, USA) was added at 1:20 ratio, followed by incubation at 37 ºC in the dark for 14 h. Trypsin reaction was stopped by adding 13.5 ul 90% formic acid. Samples were finally centrifuged at 14,000 × *g* for 15 min, transferred to a new tube and kept at – 80 °C.

### Liquid chromatography-mass spectrometry (LC–MS) analysis

Seminal plasma samples were subjected to MudPIT^108^. This analysis was carried out as two technical replicates in which each run used 25 µg of protein digest from the same animal samples. An HPLC (Agilent 1100 series) was coupled to an LTQ Velos Orbitrap (Thermo Fisher, USA) mass spectrometer equipped with a nano-electrospray ionization (ESI) source. A fused-silica microcapillary column (100 µm i.d.) was pulled with a Model P-2000 laser puller (Sutter Instrument Co., Novato, CA). A trap column was packed in-house with 3 cm of strong cation exchange resin (Partisphere SCX; Whatman, Clifton, NJ, USA) and 3 cm of 5-µm Aqua C18 resin (Phenomenex, USA). The trap was connected to an analytical C18 column (~ 15 cm fused silica; i.d. 100 µm) packed with a 3-µm Aqua C18 resin (Phenomenex, USA) and then installed on the LC–MS platform.

A fully automated 18-h, 10-step gradient (0, 10, 20, 30, 40, 50, 70, 80, 90, and 100%) was carried out on each sample. The three buffer solutions used for the chromatography were buffer A (95:5:0.1 water/acetonitrile/formic acid, v/v/v), buffer B (20:80:0.1 water/acetonitrile/formic acid, v/v/v), and buffer C (95:5:0.1 water/acetonitrile/formic acid, v/v/v with 500 mM ammonium acetate). The flow-rate was set to 400 nL/min. The mass spectrometer was operated in data-dependent mode. Each MS full scan was acquired in the orbitrap with a resolving power of 60,000 at 400 m/z. The 20 most abundant ions were selected for fragmentation via collision induced dissociation. Normalized collision energy was set to 35% and product ion scans were acquired in the ion trap.

## Data analysis

Protein identification was performed with Integrated Proteomics Pipeline (IP2—https://www.integratedproteomics.com). Raw files were extracted from Thermo raw files using RawConverter. Tandem spectra were searched with ProLuCID^111^ against a *Bos Taurus* database that was combined with a list of contaminant proteins (Uniprot release date 2018–10), and a target decoy strategy was used. Data set was searched with 50 ppm (precursor and fragment mass tolerances) and carbamidomethylation was selected as static modification. Search results were filtered with DTASelect^112,113^ where a minimum of two peptides per protein and protein false discovery rate (FDR) < 1% were required for successful protein identification. Census^114^ used protein identification results from DTASelect and generated chromatogram for each identified peptide. To increase accuracy for finding peptide precursors, we calculated person Pearson product-moment correlation coefficient comparing theoretical and experimental isotope distributions to minimize false peak detection.

### Statistical analysis

Protein intensity data generated by label free analysis were evaluated using SAS University Edition^115^. Following normality check, protein intensities in bulls of low and high sperm freezability were compared by two-tailed t test. Proteins with different abundances (p < 0.05) in the two groups of bulls were subjected to multivariate analysis using MetaboAnalyst 4.0 (https://www.metaboanalyst.ca). For this type of analysis, protein data were normalized by the sum and subjected to pareto scalling (mean-centered and divided by the square root of the standard deviation of each variable). Partial-Least Squares Discriminant Analysis (PLS-DA) was applied to differentiate classes in the complex protein datasets^15^ and Variable Importance in Projection (VIP) to identify seminal proteins for meaningful characterization of bulls with low and high sperm freezability phenotypes. For regression analysis, the freezability score of bulls (%ViableSperm) was used as the dependent variable and log-transformed abundances of proteins with the 15 highest VIP scores, as independent ones. The regression model was evaluated using a stepwise strategy of SAS package^115^. Independent variables were kept in the model if p < 0.05. Pearson’s correlations were also analyzed among abundances of seminal plasma proteins with the highest VIP scores^115^.

### Gene ontology, analysis of functional clusters and in silico protein–protein interactions

Proteins identified in bovine seminal plasma using Integrated Proteomics Pipeline (IP2) were also evaluated according to gene ontology terms for biological process and molecular function, based on UniProtKB and EBI databases. Moreover, functional clusters associated with seminal plasma proteins were analyzed through DAVID platform (DAVID Functional Annotation Bioinformatics Analysis—https://david.ncifcrf.gov)^116^. For this analysis, 1,445 Uniprot accession numbers were uploaded in the DAVID platform and clusters were defined according to enrichment scores and p-values. The STRING (https://string-db.org) version 11.0 database ^15^ was used for in silico analyses of networks for seminal plasma proteins significantly associated with sperm freezability scores of the bulls.

## Supplementary information

Supplementary Table 1.

Supplementary Table 2.

Supplementary Table 3.

Supplementary Table 4.
