# Supplementary material for: Protein signatures of seminal plasma from bulls with contrasting frozen-thawed sperm viability
Source: Sci Rep. 2020 Sep 4;10:14661. doi: 10.1038/s41598-020-71015-9 (PMC7474054; doi:10.1038/s41598-020-71015-9)
Supplement: Supplementary file 2 — Supplementary Table 2. [file 41598_2020_71015_MOESM2_ESM.pdf]

Protein Signatures of Seminal Plasma from Bulls with Contrasting Frozen-Thawed Sperm Viability

Fabio P. Gomes, Robin Park, Arabela G. Viana, Carolina Fernandez-Costa, Einko Topper, Abdullah Kaya, Erdogan Memili, John R. Yates, III, Arlindo A. Moura

Supplemental table 1. Uniprot accession numbers, gene codes, description, abundances and fold-changes of 1,445 seminal plasma proteins in bulls with low and high sperm freezability phenotypes. Proteins were identified by multidimensional protein identification technology, mass spectrometry and tools of bioinformatics. Proteins differentially expressed ($p < 0.05$) in the seminal plasma of LF and HF bulls are marked with an “X”. Relative abundance: the abundance of a specific protein as the percentage of total abundance of all proteins. AVG: average; LF: low sperm freezability; HF: high sperm freezability. Freezability scores: percentage deviation of frozen-thawed sperm viability of each bull relative to the average frozen-thawed sperm viability (54.7 ± 5.4%) measured in the population.

Functional Annotation Clustering

Current Gene List: List_1

Current Background: Bos taurus

1407 DAVID IDs

Annotation Cluster 1	Enrichment Score: 57.69	G	Count	P_Value	Benjamini
UP_KEYWORDS	Mitochondrion	RT	246	4.5E-106	4.8E-104
UP_KEYWORDS	Transit peptide	RT	131	8.6E-58	4.6E-56
UP_SEQ_FEATURE	transit peptide:Mitochondrion	RT	123	2.2E-11	6.2E-8
Annotation Cluster 2	Enrichment Score: 33.93	G	Count	P_Value	Benjamini
UP_KEYWORDS	Mitochondrion inner membrane	RT	102	7.2E-60	4.4E-58
KEGG_PATHWAY	Oxidative phosphorylation	RT	83	3.9E-46	1.0E-43
KEGG_PATHWAY	Parkinson's disease	RT	84	2.0E-43	2.7E-41
UP_KEYWORDS	Electron transport	RT	56	2.5E-38	8.2E-37
UP_KEYWORDS	Respiratory chain	RT	46	2.0E-37	6.1E-36
KEGG_PATHWAY	Huntington's disease	RT	83	2.1E-31	1.4E-29
KEGG_PATHWAY	Alzheimer's disease	RT	78	3.0E-31	1.6E-29
GOTERM_CC_DIRECT	mitochondrial respiratory chain complex I	RT	34	7.4E-25	8.9E-23
KEGG_PATHWAY	Non-alcoholic fatty liver disease (NAFLD)	RT	59	3.2E-19	9.5E-18
GOTERM_MF_DIRECT	NADH dehydrogenase (ubiquinone) activity	RT	22	1.3E-14	4.6E-12
Annotation Cluster 3	Enrichment Score: 33.14	G	Count	P_Value	Benjamini
UP_KEYWORDS	Glycoprotein	RT	305	4.4E-87	3.7E-85
UP_KEYWORDS	Secreted	RT	238	2.8E-70	2.0E-68
UP_KEYWORDS	Disulfide bond	RT	288	2.3E-41	8.0E-40
UP_KEYWORDS	Signal	RT	335	4.2E-16	5.6E-15
UP_SEQ_FEATURE	signal peptide	RT	298	5.5E-11	7.7E-8
UP_SEQ_FEATURE	disulfide bond	RT	236	3.6E-7	2.0E-4
UP_SEQ_FEATURE	glycosylation site:N-linked (GlcNAc...)	RT	254	4.2E-4	7.4E-2
Annotation Cluster 4	Enrichment Score: 17.48	G	Count	P_Value	Benjamini
UP_KEYWORDS	Chaperone	RT	55	3.1E-28	7.0E-27
GOTERM_MF_DIRECT	unfolded protein binding	RT	26	8.9E-14	2.4E-11
GOTERM_BP_DIRECT	protein folding	RT	37	1.3E-12	7.9E-10
Annotation Cluster 5	Enrichment Score: 17.21	G	Count	P_Value	Benjamini
UP_KEYWORDS	Lysosome	RT	50	1.2E-26	2.5E-25
GOTERM_CC_DIRECT	lysosome	RT	46	4.2E-15	2.3E-13
KEGG_PATHWAY	Lysosome	RT	42	4.4E-12	1.1E-10
Annotation Cluster 6	Enrichment Score: 15.03	G	Count	P_Value	Benjamini
GOTERM_MF_DIRECT	cadherin binding involved in cell-cell adhesion	RT	34	4.6E-18	2.5E-15
GOTERM_CC_DIRECT	cell-cell adherens junction	RT	36	1.2E-14	5.1E-13
GOTERM_BP_DIRECT	cell-cell adhesion	RT	31	1.4E-14	1.1E-11
Annotation Cluster 7	Enrichment Score: 14.25	G	Count	P_Value	Benjamini
UP_KEYWORDS	Nucleotide-binding	RT	217	6.4E-37	1.8E-35

Annotation Cluster 1	Enrichment Score: 57.69	G	Count	P_Value	Benjamini
UP_KEYWORDS	ATP-binding	RT	137	5.4E-16	6.7E-15
GOTERM_MF_DIRECT	ATP binding	RT	149	1.1E-4	4.4E-3
UP_SEQ_FEATURE	nucleotide phosphate-binding region:ATP	RT	64	2.8E-2	9.2E-1
Annotation Cluster 8	Enrichment Score: 13.12	G	Count	P_Value	Benjamini
UP_KEYWORDS	Tricarboxylic acid cycle	RT	20	1.2E-18	1.6E-17
GOTERM_BP_DIRECT	tricarboxylic acid cycle	RT	21	1.7E-16	1.2E-13
KEGG_PATHWAY	Citrate cycle (TCA cycle)	RT	22	6.9E-15	1.9E-13
KEGG_PATHWAY	2-Oxocarboxylic acid metabolism	RT	10	2.4E-5	2.8E-4
Annotation Cluster 9	Enrichment Score: 10.4	G	Count	P_Value	Benjamini
KEGG_PATHWAY	Proteasome	RT	31	2.0E-19	6.9E-18
GOTERM_CC_DIRECT	proteasome accessory complex	RT	13	2.2E-12	8.3E-11
KEGG_PATHWAY	Epstein-Barr virus infection	RT	17	1.4E-1	4.4E-1
Annotation Cluster 10	Enrichment Score: 9.86	G	Count	P_Value	Benjamini
KEGG_PATHWAY	Glycolysis / Gluconeogenesis	RT	28	1.5E-11	3.1E-10
UP_KEYWORDS	Glycolysis	RT	16	4.1E-11	3.4E-10
GOTERM_BP_DIRECT	glycolytic process	RT	15	4.1E-9	1.6E-6
Annotation Cluster 11	Enrichment Score: 8.61	G	Count	P_Value	Benjamini
GOTERM_BP_DIRECT	negative regulation of endopeptidase activity	RT	32	1.5E-17	2.3E-14
UP_KEYWORDS	Protease inhibitor	RT	30	7.6E-17	1.4E-15
UP_KEYWORDS	Serine protease inhibitor	RT	22	2.1E-15	2.4E-14
GOTERM_CC_DIRECT	chromaffin granule	RT	9	1.3E-7	3.2E-6
GOTERM_MF_DIRECT	serine-type endopeptidase inhibitor activity	RT	25	2.9E-7	2.4E-5
SMART	SERPIN	RT	15	5.3E-7	4.6E-5
INTERPRO	Protease inhibitor I4, serpin, conserved site	RT	15	7.1E-7	1.9E-4
INTERPRO	Serpin domain	RT	15	3.9E-6	5.6E-4
INTERPRO	Serpin family	RT	15	3.9E-6	5.6E-4
UP_SEQ_FEATURE	site:Reactive bond	RT	9	1.7E-2	8.1E-1
Annotation Cluster 12	Enrichment Score: 8.52	G	Count	P_Value	Benjamini
UP_KEYWORDS	Proteasome	RT	37	3.5E-30	8.8E-29
KEGG_PATHWAY	Proteasome	RT	31	2.0E-19	6.9E-18
GOTERM_CC_DIRECT	proteasome core complex	RT	15	1.1E-11	3.7E-10
UP_KEYWORDS	Threonine protease	RT	14	2.6E-11	2.2E-10
INTERPRO	Proteasome, subunit alpha/beta	RT	14	1.0E-10	1.1E-7
GOTERM_MF_DIRECT	threonine-type endopeptidase activity	RT	14	2.6E-9	2.8E-7
SMART	SM00948	RT	7	2.5E-6	1.2E-4
INTERPRO	Proteasome A-type subunit	RT	7	6.7E-6	8.3E-4
INTERPRO	Proteasome, alpha-subunit, N-terminal domain	RT	7	6.7E-6	8.3E-4
GOTERM_CC_DIRECT	proteasome core complex, alpha-subunit complex	RT	7	1.0E-5	2.0E-4
INTERPRO	Proteasome, beta-type subunit, conserved site	RT	8	1.2E-5	1.3E-3
INTERPRO	Proteasome B-type subunit	RT	7	8.9E-5	8.1E-3
INTERPRO	Peptidase T1A, proteasome beta-subunit	RT	3	1.0E-1	9.0E-1
GOTERM_CC_DIRECT	cytoplasmic mRNA processing body	RT	4	9.0E-1	1.0E0
Annotation Cluster 13	Enrichment Score: 7.52	G	Count	P_Value	Benjamini
UP_KEYWORDS	Hydrogen ion transport	RT	27	1.3E-19	2.0E-18
GOTERM_MF_DIRECT	proton-transporting ATPase activity, rotational mechanism	RT	14	1.2E-8	1.2E-6
GOTERM_BP_DIRECT	ATP hydrolysis coupled proton transport	RT	15	8.7E-8	2.7E-5
KEGG_PATHWAY	Collecting duct acid secretion	RT	12	3.2E-5	3.7E-4
KEGG_PATHWAY	Synaptic vesicle cycle	RT	18	1.3E-4	1.3E-3
KEGG_PATHWAY	Rheumatoid arthritis	RT	21	1.2E-3	9.6E-3
Annotation Cluster 14	Enrichment Score: 7.01	G	Count	P_Value	Benjamini
UP_KEYWORDS	Hydrogen ion transport	RT	27	1.3E-19	2.0E-18
GOTERM_CC_DIRECT	mitochondrial proton-transporting ATP synthase complex	RT	15	3.2E-11	1.0E-9

Category	Term	RT	Count	P_Value	Benjamini
Annotation Cluster 1	Enrichment Score: 57.69		Count	P_Value	Benjamini
UP_KEYWORDS	ATP synthesis	RT	12	2.1E-10	1.7E-9
GOTERM_BP_DIRECT	ATP synthesis coupled proton transport	RT	14	2.7E-9	1.2E-6
UP_KEYWORDS	CF(0)	RT	9	5.5E-7	3.2E-6
GOTERM_MF_DIRECT	proton-transporting ATP synthase activity, rotational mechanism	RT	9	8.0E-6	4.8E-4
GOTERM_MF_DIRECT	hydrogen ion transmembrane transporter activity	RT	9	8.4E-5	3.9E-3
GOTERM_CC_DIRECT	mitochondrial proton-transporting ATP synthase complex, coupling factor F(o)	RT	6	4.7E-4	6.7E-3
UP_KEYWORDS	CF(1)	RT	4	8.2E-3	2.5E-2
GOTERM_CC_DIRECT	proton-transporting ATP synthase complex, catalytic core F(1)	RT	3	2.1E-2	1.7E-1
Annotation Cluster 15	Enrichment Score: 6.51		Count	P_Value	Benjamini
UP_KEYWORDS	GTP-binding	RT	68	1.9E-21	3.3E-20
UP_KEYWORDS	Prenylation	RT	26	2.1E-11	1.9E-10
GOTERM_MF_DIRECT	GTP binding	RT	72	2.5E-11	4.5E-9
GOTERM_MF_DIRECT	GTPase activity	RT	46	5.4E-11	8.4E-9
INTERPRO	Small GTP-binding protein domain	RT	33	3.5E-7	1.2E-4
GOTERM_MF_DIRECT	GDP binding	RT	16	2.7E-6	2.0E-4
UP_SEQ_FEATURE	nucleotide phosphate-binding region:GTP	RT	61	3.5E-5	9.7E-3
UP_SEQ_FEATURE	propeptide:Removed in mature form	RT	37	8.3E-5	2.1E-2
UP_SEQ_FEATURE	short sequence motif:Effector region	RT	19	1.5E-3	1.9E-1
INTERPRO	Small GTPase superfamily	RT	21	2.9E-3	1.3E-1
INTERPRO	P-loop containing nucleoside triphosphate hydrolase	RT	88	5.2E-3	1.9E-1
UP_SEQ_FEATURE	lipid moiety-binding region:S-geranylgeranyl cysteine	RT	20	5.6E-3	4.8E-1
GOTERM_BP_DIRECT	small GTPase mediated signal transduction	RT	29	1.3E-2	4.4E-1
Annotation Cluster 16	Enrichment Score: 6.38		Count	P_Value	Benjamini
UP_KEYWORDS	Lipid metabolism	RT	55	1.1E-14	1.1E-13
UP_KEYWORDS	Fatty acid metabolism	RT	23	7.4E-10	5.8E-9
UP_KEYWORDS	Lipid biosynthesis	RT	20	3.9E-5	1.8E-4
UP_KEYWORDS	Fatty acid biosynthesis	RT	11	6.9E-4	2.5E-3
GOTERM_BP_DIRECT	fatty acid biosynthetic process	RT	7	5.7E-2	8.3E-1
Annotation Cluster 17	Enrichment Score: 5.76		Count	P_Value	Benjamini
GOTERM_BP_DIRECT	mitochondrial electron transport, ubiquinol to cytochrome c	RT	11	2.6E-8	9.0E-6
GOTERM_CC_DIRECT	mitochondrial respiratory chain complex III	RT	9	1.3E-7	3.2E-6
GOTERM_BP_DIRECT	aerobic respiration	RT	9	1.6E-3	1.1E-1
Annotation Cluster 18	Enrichment Score: 5.41		Count	P_Value	Benjamini
UP_KEYWORDS	Immunity	RT	37	7.2E-9	5.5E-8
UP_KEYWORDS	Innate immunity	RT	27	2.5E-8	1.8E-7
GOTERM_BP_DIRECT	innate immune response	RT	24	3.2E-1	1.0E0
Annotation Cluster 19	Enrichment Score: 5.34		Count	P_Value	Benjamini
UP_KEYWORDS	Blood coagulation	RT	14	3.7E-7	2.2E-6
UP_KEYWORDS	Hemostasis	RT	14	3.7E-7	2.2E-6
KEGG_PATHWAY	Complement and coagulation cascades	RT	24	6.0E-7	1.0E-5
GOTERM_BP_DIRECT	blood coagulation	RT	12	5.3E-3	2.5E-1
Annotation Cluster 20	Enrichment Score: 4.89		Count	P_Value	Benjamini
SMART	ACTIN	RT	14	2.9E-9	1.0E-6
INTERPRO	Actin-related protein	RT	14	2.3E-8	1.6E-5
UP_KEYWORDS	Oxidation	RT	10	6.2E-8	4.2E-7
INTERPRO	Actin/actin-like conserved site	RT	9	2.3E-7	9.9E-5
INTERPRO	Actin, conserved site	RT	7	8.9E-5	8.1E-3
GOTERM_BP_DIRECT	mesenchyme migration	RT	4	6.5E-3	2.9E-1
UP_KEYWORDS	Muscle protein	RT	7	2.0E-2	5.4E-2
GOTERM_BP_DIRECT	skeletal muscle thin filament assembly	RT	3	6.8E-2	8.7E-1
Annotation Cluster 21	Enrichment Score: 4.57		Count	P_Value	Benjamini
UP_KEYWORDS	Microtubule	RT	31	2.2E-8	1.6E-7

	Category	Term		Count	P_Value	Benjamini
Annotation Cluster 1		Enrichment Score: 57.69	G	Count	P_Value	Benjamini
	SMART	SM00865	RT	11	3.8E-7	6.5E-5
	SMART	SM00864	RT	11	1.1E-6	6.2E-5
	GOTERM_BP_DIRECT	microtubule-based process	RT	15	1.3E-6	3.0E-4
	INTERPRO	Tubulin/FtsZ, 2-layer sandwich domain	RT	11	1.8E-6	4.1E-4
	INTERPRO	Tubulin, C-terminal	RT	11	3.0E-6	5.3E-4
	INTERPRO	Tubulin, conserved site	RT	11	3.0E-6	5.3E-4
	INTERPRO	Tubulin	RT	11	4.9E-6	6.5E-4
	INTERPRO	Tubulin/FtsZ, C-terminal	RT	11	4.9E-6	6.5E-4
	GOTERM_MF_DIRECT	structural constituent of cytoskeleton	RT	18	5.3E-6	3.4E-4
	INTERPRO	Tubulin/FtsZ, GTPase domain	RT	11	7.8E-6	8.7E-4
	INTERPRO	Beta tubulin, autoregulation binding site	RT	6	3.3E-4	2.3E-2
	INTERPRO	Beta tubulin	RT	6	3.3E-4	2.3E-2
	INTERPRO	Alpha tubulin	RT	5	6.0E-3	2.1E-1
	GOTERM_BP_DIRECT	cytoskeleton organization	RT	15	7.0E-3	3.0E-1
	KEGG_PATHWAY	Gap junction	RT	18	7.7E-3	5.4E-2
	UP_SEQ_FEATURE	site:Involved in polymerization	RT	4	3.0E-1	1.0E0
Annotation Cluster 22		Enrichment Score: 4.42	G	Count	P_Value	Benjamini
	UP_KEYWORDS	Spermatogenesis	RT	18	1.8E-7	1.1E-6
	UP_KEYWORDS	Differentiation	RT	37	4.1E-7	2.5E-6
	UP_KEYWORDS	Developmental protein	RT	37	4.7E-4	1.8E-3
	GOTERM_BP_DIRECT	cell differentiation	RT	29	6.1E-2	8.5E-1
Annotation Cluster 23		Enrichment Score: 4.38	G	Count	P_Value	Benjamini
	GOTERM_BP_DIRECT	hydrogen ion transmembrane transport	RT	14	2.7E-6	5.3E-4
	KEGG_PATHWAY	Cardiac muscle contraction	RT	23	9.9E-6	1.3E-4
	GOTERM_MF_DIRECT	cytochrome-c oxidase activity	RT	9	2.6E-3	6.3E-2
Annotation Cluster 24		Enrichment Score: 4.31	G	Count	P_Value	Benjamini
	UP_KEYWORDS	Ubiquinone	RT	12	1.2E-7	7.8E-7
	UP_KEYWORDS	Iron-sulfur	RT	15	4.1E-6	2.1E-5
	UP_KEYWORDS	4Fe-4S	RT	10	5.1E-5	2.3E-4
	GOTERM_MF_DIRECT	4 iron, 4 sulfur cluster binding	RT	10	1.3E-3	3.5E-2
	UP_SEQ_FEATURE	metal ion-binding site:Iron-sulfur (4Fe-4S)	RT	7	9.2E-3	6.1E-1
Annotation Cluster 25		Enrichment Score: 4.2	G	Count	P_Value	Benjamini
	SMART	14_3_3	RT	7	6.7E-7	4.6E-5
	INTERPRO	14-3-3 protein	RT	7	1.8E-6	3.8E-4
	INTERPRO	14-3-3 domain	RT	7	1.8E-6	3.8E-4
	INTERPRO	14-3-3 protein, conserved site	RT	7	1.8E-6	3.8E-4
	GOTERM_MF_DIRECT	protein domain specific binding	RT	15	4.0E-6	2.7E-4
	PIR_SUPERFAMILY	14-3-3 protein	RT	7	5.6E-5	1.0E-2
	UP_SEQ_FEATURE	site:Interaction with phosphoserine on interacting protein	RT	7	1.8E-3	2.2E-1
	GOTERM_BP_DIRECT	protein targeting	RT	6	1.1E-2	4.1E-1
	KEGG_PATHWAY	Cell cycle	RT	9	9.5E-1	9.9E-1
Annotation Cluster 26		Enrichment Score: 4.16	G	Count	P_Value	Benjamini
	UP_KEYWORDS	Antibiotic	RT	16	6.9E-7	4.0E-6
	UP_KEYWORDS	Antimicrobial	RT	17	2.5E-6	1.3E-5
	GOTERM_BP_DIRECT	defense response to bacterium	RT	11	2.0E-1	9.9E-1
Annotation Cluster 27		Enrichment Score: 3.93	G	Count	P_Value	Benjamini
	INTERPRO	Chaperonin TCP-1, conserved site	RT	8	6.9E-7	2.1E-4
	GOTERM_CC_DIRECT	chaperonin-containing T-complex	RT	8	1.2E-6	2.5E-5
	INTERPRO	TCP-1-like chaperonin intermediate domain	RT	8	2.1E-6	4.1E-4
	INTERPRO	Chaperone tailless complex polypeptide 1 (TCP-1)	RT	8	2.1E-6	4.1E-4
	INTERPRO	GroEL-like equatorial domain	RT	9	3.4E-6	5.6E-4
	INTERPRO	GroEL-like apical domain	RT	9	3.4E-6	5.6E-4
	INTERPRO	Chaperonin Cpn60/TCP-1	RT	9	6.8E-6	8.0E-4

	Annotation Cluster 1	Enrichment Score: 57.69	G		Count	P_Value	Benjamini
	GOTERM_CC_DIRECT	zona pellucida receptor complex	RT		7	1.0E-5	2.0E-4
	GOTERM_BP_DIRECT	binding of sperm to zona pellucida	RT		12	1.1E-5	1.5E-3
	GOTERM_BP_DIRECT	positive regulation of protein localization to Cajal body	RT		6	1.1E-4	1.0E-2
	GOTERM_BP_DIRECT	positive regulation of establishment of protein localization to telomere	RT		5	3.5E-3	1.9E-1
	GOTERM_BP_DIRECT	positive regulation of telomerase RNA localization to Cajal body	RT		6	4.2E-3	2.2E-1
	GOTERM_BP_DIRECT	toxin transport	RT		8	7.0E-3	3.0E-1
	GOTERM_BP_DIRECT	scaRNA localization to Cajal body	RT		3	4.4E-2	7.6E-1
	GOTERM_BP_DIRECT	positive regulation of telomere maintenance via telomerase	RT		6	1.2E-1	9.4E-1
	GOTERM_BP_DIRECT	positive regulation of telomerase activity	RT		3	7.2E-1	1.0E0
	Annotation Cluster 28	Enrichment Score: 3.64	G		Count	P_Value	Benjamini
	INTERPRO	Heat shock protein 70, conserved site	RT		9	3.4E-6	5.6E-4
	INTERPRO	Heat shock protein 70 family	RT		9	3.4E-6	5.6E-4
	KEGG_PATHWAY	Spliceosome	RT		7	9.9E-1	1.0E0
	Annotation Cluster 29	Enrichment Score: 3.63	G		Count	P_Value	Benjamini
	SMART	PINT	RT		10	4.6E-7	5.3E-5
	INTERPRO	Proteasome component (PCI) domain	RT		10	3.5E-6	5.3E-4
	GOTERM_CC_DIRECT	proteasome regulatory particle, lid subcomplex	RT		6	4.7E-4	6.7E-3
	UP_SEQ_FEATURE	domain:PCI	RT		9	9.6E-4	1.4E-1
	INTERPRO	Winged helix-turn-helix DNA-binding domain	RT		12	9.7E-1	1.0E0
	Annotation Cluster 30	Enrichment Score: 3.51	G		Count	P_Value	Benjamini
	KEGG_PATHWAY	Fatty acid metabolism	RT		17	1.1E-5	1.4E-4
	KEGG_PATHWAY	Valine, leucine and isoleucine degradation	RT		16	8.6E-5	9.0E-4
	KEGG_PATHWAY	Fatty acid degradation	RT		14	1.4E-4	1.3E-3
	GOTERM_BP_DIRECT	fatty acid beta-oxidation	RT		7	6.5E-2	8.6E-1
	Annotation Cluster 31	Enrichment Score: 3.47	G		Count	P_Value	Benjamini
	INTERPRO	Thioredoxin-like fold	RT		30	9.2E-8	4.9E-5
	GOTERM_BP_DIRECT	cellular oxidant detoxification	RT		15	1.5E-7	3.8E-5
	UP_KEYWORDS	Antioxidant	RT		10	1.6E-7	1.0E-6
	UP_KEYWORDS	Redox-active center	RT		13	5.2E-7	3.1E-6
	INTERPRO	Thioredoxin domain	RT		12	1.4E-4	1.2E-2
	GOTERM_BP_DIRECT	cell redox homeostasis	RT		16	1.5E-4	1.3E-2
	UP_KEYWORDS	Peroxidase	RT		8	2.8E-4	1.1E-3
	INTERPRO	Peroxiredoxin, C-terminal	RT		4	4.6E-3	1.9E-1
	INTERPRO	Alkyl hydroperoxide reductase subunit C/ Thiol specific antioxidant	RT		4	4.6E-3	1.9E-1
	GOTERM_MF_DIRECT	peroxiredoxin activity	RT		4	6.4E-3	1.2E-1
	INTERPRO	Peroxiredoxin, AhpC-type	RT		3	1.9E-2	4.2E-1
	UP_SEQ_FEATURE	active site:Cysteine sulfenic acid (-SOH) intermediate	RT		5	4.3E-2	9.6E-1
	GOTERM_MF_DIRECT	thioredoxin peroxidase activity	RT		3	4.3E-2	4.5E-1
	UP_SEQ_FEATURE	domain:Thioredoxin	RT		8	5.3E-2	9.8E-1
	PIR_SUPERFAMILY	peroxiredoxin, AhpC type	RT		3	5.8E-2	9.7E-1
	Annotation Cluster 32	Enrichment Score: 3.1	G		Count	P_Value	Benjamini
	UP_KEYWORDS	Milk protein	RT		7	1.6E-6	8.7E-6
	GOTERM_BP_DIRECT	response to dehydroepiandrosterone	RT		5	3.1E-4	2.5E-2
	GOTERM_BP_DIRECT	response to 11-deoxycorticosterone	RT		5	3.1E-4	2.5E-2
	GOTERM_BP_DIRECT	response to progesterone	RT		6	1.8E-3	1.2E-1
	GOTERM_CC_DIRECT	Golgi lumen	RT		5	3.0E-3	3.3E-2
	GOTERM_BP_DIRECT	response to estradiol	RT		5	1.3E-2	4.4E-1
	INTERPRO	Casein, alpha/beta	RT		3	1.9E-2	4.2E-1
	Annotation Cluster 33	Enrichment Score: 3.05	G		Count	P_Value	Benjamini
	GOTERM_BP_DIRECT	proteolysis involved in cellular protein catabolic process	RT		16	1.2E-7	3.5E-5
	UP_KEYWORDS	Thiol protease	RT		18	9.9E-6	4.9E-5

Annotation Cluster 1	Enrichment Score: 57.69		Count	P_Value	Benjamini
INTERPRO	Cysteine peptidase, asparagine active site	RT	7	8.9E-5	8.1E-3
SMART	Pept_C1	RT	7	1.1E-4	3.6E-3
INTERPRO	Peptidase C1A, papain C-terminal	RT	7	2.9E-4	2.1E-2
INTERPRO	Peptidase C1A, papain	RT	7	2.9E-4	2.1E-2
INTERPRO	Cysteine peptidase, histidine active site	RT	6	1.0E-3	5.6E-2
INTERPRO	Cysteine peptidase, cysteine active site	RT	7	5.2E-3	1.9E-1
GOTERM_MF_DIRECT	cysteine-type endopeptidase activity	RT	10	5.8E-3	1.1E-1
GOTERM_MF_DIRECT	cysteine-type peptidase activity	RT	6	6.0E-3	1.1E-1
SMART	SM00848	RT	4	9.1E-3	1.5E-1
INTERPRO	Proteinase inhibitor I29, cathepsin propeptide	RT	4	1.4E-2	3.7E-1
GOTERM_BP_DIRECT	cellular response to thyroid hormone stimulus	RT	4	5.5E-2	8.3E-1
UP_SEQ_FEATURE	propeptide:Activation peptide	RT	11	1.5E-1	1.0E0

Annotation Cluster 34	Enrichment Score: 2.61		Count	P_Value	Benjamini
GOTERM_MF_DIRECT	G-protein beta/gamma-subunit complex binding	RT	9	1.6E-5	9.0E-4
SMART	SM00275	RT	8	4.5E-5	1.7E-3
GOTERM_MF_DIRECT	G-protein coupled receptor binding	RT	12	4.9E-5	2.5E-3
INTERPRO	G protein alpha subunit, helical insertion	RT	8	1.3E-4	1.2E-2
INTERPRO	Guanine nucleotide binding protein (G-protein), alpha subunit	RT	8	1.3E-4	1.2E-2
INTERPRO	G-protein alpha subunit, group I	RT	5	2.3E-3	1.1E-1
GOTERM_CC_DIRECT	heterotrimeric G-protein complex	RT	8	1.3E-2	1.1E-1
GOTERM_MF_DIRECT	signal transducer activity	RT	20	2.9E-2	3.6E-1
GOTERM_BP_DIRECT	adenylate cyclase-modulating G-protein coupled receptor signaling pathway	RT	5	8.7E-2	9.1E-1
KEGG_PATHWAY	Long-term depression	RT	8	4.0E-1	7.3E-1
UP_KEYWORDS	Transducer	RT	18	1.0E0	1.0E0

Annotation Cluster 35	Enrichment Score: 2.58		Count	P_Value	Benjamini
KEGG_PATHWAY	2-Oxocarboxylic acid metabolism	RT	10	2.4E-5	2.8E-4
SMART	SM01329	RT	5	1.0E-4	3.5E-3
INTERPRO	Isopropylmalate dehydrogenase-like domain	RT	5	2.0E-4	1.5E-2
INTERPRO	Isocitrate/isopropylmalate dehydrogenase, conserved site	RT	5	2.0E-4	1.5E-2
UP_SEQ_FEATURE	site:Critical for catalysis	RT	5	8.8E-3	6.2E-1
INTERPRO	Isocitrate dehydrogenase NAD-dependent	RT	3	1.9E-2	4.2E-1
INTERPRO	Isocitrate and isopropylmalate dehydrogenases family	RT	3	1.9E-2	4.2E-1
GOTERM_MF_DIRECT	isocitrate dehydrogenase (NAD+) activity	RT	3	2.3E-2	3.3E-1
GOTERM_BP_DIRECT	isocitrate metabolic process	RT	3	2.3E-2	5.9E-1
UP_SEQ_FEATURE	metal ion-binding site:Magnesium or manganese	RT	5	1.1E-1	1.0E0

Annotation Cluster 36	Enrichment Score: 2.46		Count	P_Value	Benjamini
UP_KEYWORDS	Cell junction	RT	40	5.9E-5	2.6E-4
UP_KEYWORDS	Synapse	RT	19	2.4E-2	6.3E-2
GOTERM_CC_DIRECT	cell junction	RT	28	3.0E-2	2.1E-1

Annotation Cluster 37	Enrichment Score: 2.45		Count	P_Value	Benjamini
SMART	RNAse_Pc	RT	9	6.5E-6	2.8E-4
INTERPRO	Ribonuclease A, active site	RT	8	1.2E-5	1.3E-3
INTERPRO	Ribonuclease A	RT	9	2.2E-5	2.2E-3
INTERPRO	Ribonuclease A-domain	RT	9	2.2E-5	2.2E-3
GOTERM_MF_DIRECT	endonuclease activity	RT	10	2.1E-4	7.9E-3
UP_KEYWORDS	Endonuclease	RT	13	3.3E-4	1.3E-3
UP_KEYWORDS	Nuclease	RT	15	1.5E-3	5.3E-3
GOTERM_BP_DIRECT	RNA phosphodiester bond hydrolysis	RT	4	6.5E-3	2.9E-1
GOTERM_MF_DIRECT	ribonuclease A activity	RT	3	2.3E-2	3.3E-1
GOTERM_MF_DIRECT	ribonuclease activity	RT	5	6.1E-2	5.5E-1
UP_SEQ_FEATURE	chain:Angiogenin-1	RT	3	2.0E-1	1.0E0

			Count	P_Value	Benjamini
Annotation Cluster 1	Enrichment Score: 57.69		Count	P_Value	Benjamini
UP_SEQ_FEATURE	chain:Angiogenin-2	RT	3	2.0E-1	1.0E0
UP_SEQ_FEATURE	chain:Ribonuclease 4	RT	3	2.0E-1	1.0E0
GOTERM_BP_DIRECT	RNA phosphodiester bond hydrolysis, endonucleolytic	RT	4	4.7E-1	1.0E0
GOTERM_MF_DIRECT	nucleic acid binding	RT	13	1.0E0	1.0E0
Annotation Cluster 38	Enrichment Score: 2.42		Count	P_Value	Benjamini
KEGG_PATHWAY	Pyruvate metabolism	RT	15	1.1E-5	1.4E-4
KEGG_PATHWAY	Cysteine and methionine metabolism	RT	14	5.6E-5	6.1E-4
INTERPRO	Lactate dehydrogenase/glycoside hydrolase, family 4, C-terminal	RT	6	1.6E-4	1.3E-2
INTERPRO	Lactate/malate dehydrogenase, N-terminal	RT	5	1.2E-3	6.4E-2
INTERPRO	Lactate/malate dehydrogenase, C-terminal	RT	5	1.2E-3	6.4E-2
INTERPRO	L-lactate/malate dehydrogenase	RT	5	1.2E-3	6.4E-2
PIR_SUPERFAMILY	L-lactate/malate dehydrogenase	RT	5	5.2E-3	3.8E-1
INTERPRO	L-lactate dehydrogenase, active site	RT	3	3.5E-2	5.9E-1
INTERPRO	L-lactate dehydrogenase	RT	3	3.5E-2	5.9E-1
UP_SEQ_FEATURE	binding site:NAD	RT	11	3.7E-2	9.5E-1
GOTERM_MF_DIRECT	L-lactate dehydrogenase activity	RT	3	4.3E-2	4.5E-1
GOTERM_BP_DIRECT	carboxylic acid metabolic process	RT	4	7.1E-2	8.7E-1
UP_SEQ_FEATURE	binding site:NAD or substrate	RT	3	2.9E-1	1.0E0
Annotation Cluster 39	Enrichment Score: 2.33		Count	P_Value	Benjamini
INTERPRO	GroES-like	RT	8	6.7E-4	4.1E-2
INTERPRO	Alcohol dehydrogenase, C-terminal	RT	7	7.3E-4	4.3E-2
SMART	SM00829	RT	6	2.5E-3	6.0E-2
INTERPRO	Polyketide synthase, enoylreductase	RT	6	5.2E-3	2.0E-1
INTERPRO	Alcohol dehydrogenase superfamily, zinc-type	RT	6	1.2E-2	3.4E-1
INTERPRO	Alcohol dehydrogenase GroES-like	RT	4	1.3E-1	9.4E-1
Annotation Cluster 40	Enrichment Score: 2.31		Count	P_Value	Benjamini
UP_KEYWORDS	Cilium biogenesis/degradation	RT	14	1.3E-5	6.1E-5
GOTERM_BP_DIRECT	cilium morphogenesis	RT	12	9.5E-3	3.7E-1
GOTERM_BP_DIRECT	cilium assembly	RT	6	9.4E-1	1.0E0
Annotation Cluster 41	Enrichment Score: 2.27		Count	P_Value	Benjamini
INTERPRO	ATP-citrate lyase/succinyl-CoA ligase	RT	4	2.0E-3	9.5E-2
INTERPRO	Succinyl-CoA synthetase-like	RT	4	2.0E-3	9.5E-2
INTERPRO	ATP-grasp fold, subdomain 2	RT	6	1.2E-2	3.4E-1
INTERPRO	Succinyl-CoA synthetase, beta subunit, conserved site	RT	3	1.9E-2	4.2E-1
Annotation Cluster 42	Enrichment Score: 2.18		Count	P_Value	Benjamini
INTERPRO	ATPase, alpha/beta subunit, nucleotide-binding domain, active site	RT	4	4.6E-3	1.9E-1
INTERPRO	ATPase, alpha/beta subunit, N-terminal	RT	4	4.6E-3	1.9E-1
INTERPRO	ATPase, F1/V1/A1 complex, alpha/beta subunit, nucleotide-binding domain	RT	4	4.6E-3	1.9E-1
INTERPRO	ATPase, F1/V1/A1 complex, alpha/beta subunit, C-terminal	RT	3	1.9E-2	4.2E-1
Annotation Cluster 43	Enrichment Score: 2.17		Count	P_Value	Benjamini
UP_KEYWORDS	Complement alternate pathway	RT	5	1.1E-3	3.9E-3
GOTERM_BP_DIRECT	complement activation, alternative pathway	RT	5	1.9E-3	1.2E-1
KEGG_PATHWAY	Staphylococcus aureus infection	RT	10	1.4E-1	4.3E-1
Annotation Cluster 44	Enrichment Score: 2.17		Count	P_Value	Benjamini
GOTERM_MF_DIRECT	cytochrome-c oxidase activity	RT	9	2.6E-3	6.3E-2
GOTERM_BP_DIRECT	mitochondrial electron transport, cytochrome c to oxygen	RT	6	2.8E-3	1.6E-1
GOTERM_CC_DIRECT	respiratory chain complex IV	RT	6	9.7E-3	9.0E-2
GOTERM_CC_DIRECT	mitochondrial respiratory chain complex IV	RT	5	2.8E-2	2.1E-1
Annotation Cluster 45	Enrichment Score: 2.14		Count	P_Value	Benjamini
GOTERM_BP_DIRECT	defense response to Gram-negative bacterium	RT	13	1.1E-4	1.0E-2
SMART	LYZ1	RT	6	3.4E-3	7.6E-2
INTERPRO	Glycoside hydrolase, family 22	RT	6	7.0E-3	2.3E-1

	Annotation Cluster 1	Enrichment Score: 57.69	G		Count	P_Value	Benjamini
	INTERPRO	Lysozyme-like domain	RT		6	9.3E-3	2.8E-1
	GOTERM_BP_DIRECT	cell wall macromolecule catabolic process	RT		6	1.1E-2	4.1E-1
	GOTERM_MF_DIRECT	lysozyme activity	RT		6	1.5E-2	2.4E-1
	INTERPRO	Glycoside hydrolase, family 22, conserved site	RT		5	2.2E-2	4.6E-1
	GOTERM_BP_DIRECT	defense response to Gram-positive bacterium	RT		12	2.3E-2	5.8E-1
	INTERPRO	Glycoside hydrolase, family 22, lysozyme	RT		5	2.8E-2	5.3E-1
	Annotation Cluster 46	Enrichment Score: 2.11	G		Count	P_Value	Benjamini
	INTERPRO	Lactate dehydrogenase/glycoside hydrolase, family 4, C-terminal	RT		6	1.6E-4	1.3E-2
	GOTERM_MF_DIRECT	L-malate dehydrogenase activity	RT		3	2.3E-2	3.3E-1
	GOTERM_BP_DIRECT	malate metabolic process	RT		3	1.3E-1	9.6E-1
	Annotation Cluster 47	Enrichment Score: 2.1	G		Count	P_Value	Benjamini
	INTERPRO	Mitochondrial carrier protein	RT		9	5.5E-4	3.6E-2
	INTERPRO	Mitochondrial substrate/solute carrier	RT		13	6.0E-4	3.8E-2
	INTERPRO	Mitochondrial carrier domain	RT		13	6.0E-4	3.8E-2
	UP_SEQ_FEATURE	repeat:Solcar 1	RT		13	4.3E-3	4.2E-1
	UP_SEQ_FEATURE	repeat:Solcar 2	RT		13	4.3E-3	4.2E-1
	UP_SEQ_FEATURE	repeat:Solcar 3	RT		10	4.2E-2	9.7E-1
	GOTERM_MF_DIRECT	structural constituent of ribosome	RT		28	4.4E-2	4.5E-1
	GOTERM_BP_DIRECT	translation	RT		22	9.9E-2	9.3E-1
	GOTERM_BP_DIRECT	transmembrane transport	RT		14	2.0E-1	9.9E-1
	Annotation Cluster 48	Enrichment Score: 1.95	G		Count	P_Value	Benjamini
	INTERPRO	Mitochondrial carrier protein	RT		9	5.5E-4	3.6E-2
	INTERPRO	Adenine nucleotide translocator 1	RT		4	2.0E-3	9.5E-2
	UP_SEQ_FEATURE	short sequence motif:Substrate recognition	RT		4	3.3E-2	9.4E-1
	UP_SEQ_FEATURE	repeat:Solcar 3	RT		10	4.2E-2	9.7E-1
	UP_SEQ_FEATURE	binding site:Nucleotide	RT		3	1.2E-1	1.0E0
	Annotation Cluster 49	Enrichment Score: 1.93	G		Count	P_Value	Benjamini
	GOTERM_CC_DIRECT	proteasome regulatory particle, base subcomplex	RT		8	2.0E-5	3.7E-4
	GOTERM_CC_DIRECT	nuclear proteasome complex	RT		5	3.0E-3	3.3E-2
	INTERPRO	ATPase, AAA-type, conserved site	RT		8	4.6E-3	1.9E-1
	INTERPRO	ATPase, AAA-type, core	RT		10	7.6E-3	2.5E-1
	INTERPRO	26S proteasome subunit P45	RT		4	8.7E-3	2.7E-1
	GOTERM_BP_DIRECT	positive regulation of proteasomal protein catabolic process	RT		5	9.1E-3	3.6E-1
	GOTERM_MF_DIRECT	proteasome-activating ATPase activity	RT		4	1.2E-2	2.0E-1
	GOTERM_CC_DIRECT	cytosolic proteasome complex	RT		4	1.8E-2	1.4E-1
	GOTERM_BP_DIRECT	positive regulation of RNA polymerase II transcriptional preinitiation complex assembly	RT		4	2.0E-2	5.4E-1
	GOTERM_MF_DIRECT	TBP-class protein binding	RT		4	5.5E-2	5.2E-1
	GOTERM_BP_DIRECT	ER-associated ubiquitin-dependent protein catabolic process	RT		9	6.5E-2	8.6E-1
	SMART	AAA	RT		14	1.2E-1	7.2E-1
	INTERPRO	AAA+ ATPase domain	RT		14	2.7E-1	9.9E-1
	Annotation Cluster 50	Enrichment Score: 1.9	G		Count	P_Value	Benjamini
	INTERPRO	Kringle-like fold	RT		9	7.4E-4	4.3E-2
	SMART	FN2	RT		6	1.8E-3	4.6E-2
	INTERPRO	Fibronectin, type II, collagen-binding	RT		6	3.7E-3	1.6E-1
	GOTERM_BP_DIRECT	sperm capacitation	RT		5	6.2E-2	8.5E-1
	UP_SEQ_FEATURE	domain:Fibronectin type-II 2	RT		4	1.2E-1	1.0E0
	UP_SEQ_FEATURE	domain:Fibronectin type-II 1	RT		4	1.2E-1	1.0E0
	Annotation Cluster 51	Enrichment Score: 1.87	G		Count	P_Value	Benjamini
	UP_SEQ_FEATURE	metal ion-binding site:Manganese	RT		12	8.3E-6	2.9E-3
	GOTERM_BP_DIRECT	protein dephosphorylation	RT		13	1.7E-5	2.3E-3
	GOTERM_MF_DIRECT	phosphoprotein phosphatase activity	RT		10	1.0E-4	4.3E-3

	Category	Term		Count	P_Value	Benjamini
	Annotation Cluster 1	Enrichment Score: 57.69	G	Count	P_Value	Benjamini
☐	INTERPRO	Metallophosphoesterase domain	RT	10	1.8E-4	1.4E-2
☐	GOTERM_MF_DIRECT	phosphatase activity	RT	8	6.4E-4	1.9E-2
☐	SMART	PP2Ac	RT	6	1.2E-3	3.4E-2
☐	INTERPRO	Serine/threonine-specific protein phosphatase/bis(5-nucleosyl)-tetraphosphatase	RT	6	2.5E-3	1.1E-1
☐	GOTERM_MF_DIRECT	protein serine/threonine phosphatase activity	RT	10	5.8E-3	1.1E-1
☐	UP_KEYWORDS	Protein phosphatase	RT	14	8.6E-3	2.6E-2
☐	UP_KEYWORDS	Glycogen metabolism	RT	5	1.1E-2	3.3E-2
☐	GOTERM_CC_DIRECT	MLL5-L complex	RT	4	2.7E-2	2.0E-1
☐	UP_KEYWORDS	Biological rhythms	RT	7	5.4E-2	1.3E-1
☐	GOTERM_BP_DIRECT	glycogen metabolic process	RT	5	6.2E-2	8.5E-1
☐	GOTERM_CC_DIRECT	PTW/PP1 phosphatase complex	RT	3	1.2E-1	5.2E-1
☐	GOTERM_BP_DIRECT	entrainment of circadian clock by photoperiod	RT	4	2.0E-1	9.9E-1
☐	UP_SEQ_FEATURE	metal ion-binding site:Iron	RT	9	2.7E-1	1.0E0
☐	KEGG_PATHWAY	mRNA surveillance pathway	RT	9	7.0E-1	9.0E-1
☐	GOTERM_BP_DIRECT	circadian regulation of gene expression	RT	4	8.0E-1	1.0E0
☐	GOTERM_BP_DIRECT	regulation of circadian rhythm	RT	3	8.0E-1	1.0E0
☐	GOTERM_CC_DIRECT	nuclear chromosome, telomeric region	RT	6	8.9E-1	1.0E0
☐	KEGG_PATHWAY	Insulin resistance	RT	9	8.9E-1	9.7E-1
	Annotation Cluster 52	Enrichment Score: 1.86	G	Count	P_Value	Benjamini
☐	GOTERM_BP_DIRECT	translational elongation	RT	8	5.3E-5	5.6E-3
☐	UP_KEYWORDS	Elongation factor	RT	7	2.5E-4	1.0E-3
☐	GOTERM_MF_DIRECT	translation elongation factor activity	RT	7	3.9E-3	8.4E-2
☐	GOTERM_CC_DIRECT	eukaryotic translation elongation factor 1 complex	RT	3	2.1E-2	1.7E-1
☐	INTERPRO	Translation elongation factor EFTu/EF1A, domain 2	RT	3	2.8E-1	1.0E0
☐	INTERPRO	Elongation factor, GTP-binding domain	RT	3	4.6E-1	1.0E0
☐	INTERPRO	Translation elongation/initiation factor/Ribosomal, beta-barrel	RT	3	6.5E-1	1.0E0
	Annotation Cluster 53	Enrichment Score: 1.82	G	Count	P_Value	Benjamini
☐	UP_KEYWORDS	Transmembrane beta strand	RT	5	1.1E-3	3.9E-3
☐	INTERPRO	Porin domain	RT	3	5.5E-2	7.3E-1
☐	INTERPRO	Eukaryotic porin/Tom40	RT	3	5.5E-2	7.3E-1
	Annotation Cluster 54	Enrichment Score: 1.76	G	Count	P_Value	Benjamini
☐	UP_KEYWORDS	Protein biosynthesis	RT	23	1.2E-5	5.9E-5
☐	UP_KEYWORDS	Ligase	RT	26	5.0E-5	2.3E-4
☐	UP_KEYWORDS	Aminoacyl-tRNA synthetase	RT	9	2.3E-3	7.7E-3
☐	INTERPRO	Rossmann-like alpha/beta/alpha sandwich fold	RT	8	4.9E-2	7.0E-1
☐	KEGG_PATHWAY	Aminoacyl-tRNA biosynthesis	RT	9	8.2E-2	3.2E-1
☐	INTERPRO	Aminoacyl-tRNA synthetase, class I, conserved site	RT	4	1.2E-1	9.2E-1
☐	INTERPRO	Aminoacyl-tRNA synthetase, class II (G/ H/ P/ S), conserved domain	RT	3	1.3E-1	9.4E-1
☐	INTERPRO	Aminoacyl-tRNA synthetase, class II	RT	4	1.5E-1	9.6E-1
☐	UP_SEQ_FEATURE	short sequence motif:"KMSKS" region	RT	3	3.8E-1	1.0E0
☐	UP_SEQ_FEATURE	short sequence motif:"HIGH" region	RT	3	4.6E-1	1.0E0
	Annotation Cluster 55	Enrichment Score: 1.76	G	Count	P_Value	Benjamini
☐	UP_KEYWORDS	Thiamine pyrophosphate	RT	5	5.5E-3	1.7E-2
☐	SMART	SM00861	RT	4	1.4E-2	2.1E-1
☐	INTERPRO	Transketolase-like, pyrimidine-binding domain	RT	4	2.2E-2	4.6E-1
☐	INTERPRO	Transketolase, C-terminal/Pyruvate-ferredoxin oxidoreductase, domain II	RT	3	5.5E-2	7.3E-1
	Annotation Cluster 56	Enrichment Score: 1.75	G	Count	P_Value	Benjamini
☐	GOTERM_MF_DIRECT	serine-type endopeptidase activity	RT	30	8.6E-5	3.9E-3
☐	UP_KEYWORDS	Serine protease	RT	21	1.6E-4	6.8E-4
☐	UP_SEQ_FEATURE	active site:Charge relay system	RT	29	2.4E-4	5.5E-2

	Annotation Cluster 1	Enrichment Score: 57.69	G			Count	P_Value	Benjamini
	SMART	Tryp_SPc	RT			13	8.1E-2	6.2E-1
	INTERPRO	Trypsin-like cysteine/serine peptidase domain	RT			15	1.1E-1	9.1E-1
	UP_SEQ_FEATURE	domain:Peptidase S1	RT			13	1.3E-1	1.0E0
	INTERPRO	Peptidase S1	RT			13	2.1E-1	9.8E-1
	INTERPRO	Peptidase S1A, chymotrypsin-type	RT			10	4.7E-1	1.0E0
	INTERPRO	Peptidase S1, trypsin family, active site	RT			9	4.8E-1	1.0E0
	Annotation Cluster 57	Enrichment Score: 1.74	G			Count	P_Value	Benjamini
	UP_KEYWORDS	Glycosidase	RT			14	1.3E-3	4.5E-3
	KEGG_PATHWAY	Other glycan degradation	RT			7	1.2E-2	7.6E-2
	INTERPRO	Glycoside hydrolase, superfamily	RT			10	1.4E-2	3.6E-1
	INTERPRO	Glycoside hydrolase, catalytic domain	RT			7	4.6E-2	6.8E-1
	KEGG_PATHWAY	Glycosaminoglycan degradation	RT			5	2.0E-1	5.1E-1
	Annotation Cluster 58	Enrichment Score: 1.73	G			Count	P_Value	Benjamini
	UP_KEYWORDS	Carboxypeptidase	RT			7	2.6E-3	8.9E-3
	GOTERM_BP_DIRECT	peptide catabolic process	RT			7	5.3E-3	2.5E-1
	GOTERM_MF_DIRECT	carboxypeptidase activity	RT			3	6.8E-2	5.9E-1
	GOTERM_MF_DIRECT	metallodipeptidase activity	RT			3	1.3E-1	7.8E-1
	Annotation Cluster 59	Enrichment Score: 1.72	G			Count	P_Value	Benjamini
	UP_KEYWORDS	Growth factor binding	RT			6	5.5E-4	2.0E-3
	SMART	IB	RT			6	4.6E-3	8.9E-2
	INTERPRO	Insulin-like growth factor binding protein	RT			4	8.7E-3	2.7E-1
	INTERPRO	Insulin-like growth factor-binding protein family 1-6, chordata	RT			4	8.7E-3	2.7E-1
	GOTERM_MF_DIRECT	insulin-like growth factor II binding	RT			4	1.2E-2	2.0E-1
	GOTERM_MF_DIRECT	insulin-like growth factor I binding	RT			4	1.2E-2	2.0E-1
	INTERPRO	Insulin-like growth factor binding protein, N-terminal, Cys-rich conserved site	RT			4	1.4E-2	3.7E-1
	INTERPRO	Insulin-like growth factor-binding protein, IGFBP	RT			6	1.5E-2	3.7E-1
	SMART	TY	RT			5	1.6E-2	2.4E-1
	INTERPRO	Thyroglobulin type-1	RT			5	2.8E-2	5.3E-1
	GOTERM_BP_DIRECT	positive regulation of insulin-like growth factor receptor signaling pathway	RT			4	3.0E-2	6.6E-1
	GOTERM_BP_DIRECT	regulation of insulin-like growth factor receptor signaling pathway	RT			4	3.0E-2	6.6E-1
	UP_SEQ_FEATURE	domain:Thyroglobulin type-1	RT			5	4.3E-2	9.6E-1
	GOTERM_BP_DIRECT	regulation of glucose metabolic process	RT			5	6.2E-2	8.5E-1
	UP_SEQ_FEATURE	domain:IGFBP N-terminal	RT			5	7.1E-2	9.9E-1
	GOTERM_BP_DIRECT	regulation of cell growth	RT			7	1.0E-1	9.3E-1
	GOTERM_BP_DIRECT	type B pancreatic cell proliferation	RT			3	1.3E-1	9.6E-1
	Annotation Cluster 60	Enrichment Score: 1.7	G			Count	P_Value	Benjamini
	INTERPRO	Biotin/lipoyl attachment	RT			5	6.0E-3	2.1E-1
	INTERPRO	Single hybrid motif	RT			5	8.8E-3	2.7E-1
	UP_KEYWORDS	Lipoyl	RT			3	5.3E-2	1.3E-1
	INTERPRO	2-oxo acid dehydrogenase, lipoyl-binding site	RT			3	5.5E-2	7.3E-1
	Annotation Cluster 61	Enrichment Score: 1.7	G			Count	P_Value	Benjamini
	UP_SEQ_FEATURE	metal ion-binding site:Magnesium 2	RT			12	5.9E-4	9.6E-2
	UP_SEQ_FEATURE	metal ion-binding site:Magnesium 1	RT			13	6.8E-4	1.1E-1
	INTERPRO	Inositol monophosphatase	RT			4	4.6E-3	1.9E-1
	INTERPRO	Inositol monophosphatase, conserved site	RT			4	4.6E-3	1.9E-1
	UP_SEQ_FEATURE	metal ion-binding site:Magnesium 1; via carbonyl oxygen	RT			5	8.8E-3	6.2E-1
	UP_KEYWORDS	Lithium	RT			3	1.8E-2	4.9E-2
	INTERPRO	Inositol monophosphatase, metal-binding site	RT			3	3.5E-2	5.9E-1
	GOTERM_BP_DIRECT	phosphatidylinositol phosphorylation	RT			4	3.2E-1	1.0E0
	KEGG_PATHWAY	Inositol phosphate metabolism	RT			7	7.4E-1	9.1E-1
	KEGG_PATHWAY	Phosphatidylinositol signaling system	RT			6	9.8E-1	1.0E0

Category	Term		Count	P_Value	Benjamini
Annotation Cluster 1	Enrichment Score: 57.69	G	Count	P_Value	Benjamini
Annotation Cluster 62	Enrichment Score: 1.67	G	Count	P_Value	Benjamini
INTERPRO	Thioredoxin domain	RT	12	1.4E-4	1.2E-2
GOTERM_BP_DIRECT	response to endoplasmic reticulum stress	RT	10	2.1E-2	5.6E-1
INTERPRO	Protein disulphide isomerase	RT	3	3.5E-2	5.9E-1
INTERPRO	Disulphide isomerase	RT	3	3.5E-2	5.9E-1
INTERPRO	Thioredoxin, conserved site	RT	5	4.3E-2	6.6E-1
GOTERM_MF_DIRECT	protein disulfide isomerase activity	RT	5	6.1E-2	5.5E-1
UP_SEQ_FEATURE	domain:Thioredoxin 1	RT	4	7.0E-2	9.9E-1
UP_SEQ_FEATURE	domain:Thioredoxin 2	RT	4	7.0E-2	9.9E-1
Annotation Cluster 63	Enrichment Score: 1.65	G	Count	P_Value	Benjamini
GOTERM_MF_DIRECT	growth factor activity	RT	15	4.7E-3	9.6E-2
UP_KEYWORDS	Mitogen	RT	6	1.4E-2	3.9E-2
UP_KEYWORDS	Growth factor	RT	14	5.4E-2	1.3E-1
GOTERM_BP_DIRECT	positive regulation of cell division	RT	6	7.1E-2	8.7E-1
Annotation Cluster 64	Enrichment Score: 1.58	G	Count	P_Value	Benjamini
INTERPRO	Carbon-nitrogen hydrolase	RT	4	1.4E-2	3.7E-1
GOTERM_BP_DIRECT	nitrogen compound metabolic process	RT	5	1.8E-2	5.3E-1
UP_SEQ_FEATURE	domain:CN hydrolase	RT	4	7.0E-2	9.9E-1
Annotation Cluster 65	Enrichment Score: 1.57	G	Count	P_Value	Benjamini
UP_KEYWORDS	Multifunctional enzyme	RT	9	3.5E-4	1.4E-3
INTERPRO	ATP-grasp fold, subdomain 2	RT	6	1.2E-2	3.4E-1
INTERPRO	ATP-grasp fold, subdomain 1	RT	5	2.8E-2	5.3E-1
UP_SEQ_FEATURE	domain:ATP-grasp	RT	5	4.3E-2	9.6E-1
INTERPRO	Rudiment single hybrid motif	RT	3	7.9E-2	8.4E-1
INTERPRO	ATP-grasp fold	RT	4	8.2E-2	8.5E-1
INTERPRO	Pre-ATP-grasp domain	RT	3	3.1E-1	1.0E0
Annotation Cluster 66	Enrichment Score: 1.54	G	Count	P_Value	Benjamini
UP_KEYWORDS	Flavoprotein	RT	21	7.5E-6	3.8E-5
UP_KEYWORDS	FAD	RT	20	1.1E-5	5.4E-5
GOTERM_BP_DIRECT	fatty acid beta-oxidation using acyl-CoA dehydrogenase	RT	7	2.8E-3	1.6E-1
COG_ONTOLOGY	Lipid metabolism	RT	11	6.3E-3	5.6E-2
UP_SEQ_FEATURE	nucleotide phosphate-binding region:FAD	RT	12	2.5E-2	8.9E-1
INTERPRO	Acyl-CoA dehydrogenase, conserved site	RT	4	3.1E-2	5.5E-1
GOTERM_MF_DIRECT	fatty-acyl-CoA binding	RT	6	7.0E-2	5.9E-1
INTERPRO	Acyl-CoA dehydrogenase/oxidase, N-terminal	RT	4	8.2E-2	8.5E-1
INTERPRO	Acyl-CoA oxidase/dehydrogenase, central domain	RT	4	8.2E-2	8.5E-1
INTERPRO	Acyl-CoA dehydrogenase/oxidase C-terminal	RT	4	9.8E-2	8.9E-1
INTERPRO	Acyl-CoA dehydrogenase/oxidase	RT	4	9.8E-2	8.9E-1
GOTERM_BP_DIRECT	lipid homeostasis	RT	6	1.2E-1	9.4E-1
GOTERM_MF_DIRECT	oxidoreductase activity, acting on the CH-CH group of donors, with a flavin as acceptor	RT	4	1.3E-1	7.8E-1
GOTERM_MF_DIRECT	flavin adenine dinucleotide binding	RT	9	1.7E-1	8.6E-1
UP_SEQ_FEATURE	binding site:Substrate; via amide nitrogen	RT	6	2.0E-1	1.0E0
GOTERM_MF_DIRECT	acyl-CoA dehydrogenase activity	RT	3	3.3E-1	9.7E-1
UP_SEQ_FEATURE	binding site:FAD	RT	4	3.7E-1	1.0E0
UP_SEQ_FEATURE	binding site:Substrate; via carbonyl oxygen	RT	5	5.1E-1	1.0E0
Annotation Cluster 67	Enrichment Score: 1.53	G	Count	P_Value	Benjamini
GOTERM_BP_DIRECT	nucleotide biosynthetic process	RT	5	5.9E-3	2.7E-1
INTERPRO	Phosphoribosyltransferase domain	RT	5	1.2E-2	3.4E-1
UP_KEYWORDS	Nucleotide biosynthesis	RT	3	3.4E-2	8.6E-2
INTERPRO	Ribose-phosphate diphosphokinase	RT	3	5.5E-2	7.3E-1
GOTERM_MF_DIRECT	ribose phosphate diphosphokinase activity	RT	3	6.8E-2	5.9E-1
GOTERM_BP_DIRECT	nucleoside metabolic process	RT	4	7.1E-2	8.7E-1

	Category	Term		Count	P_Value	Benjamini
	Annotation Cluster 1	Enrichment Score: 57.69	G	Count	P_Value	Benjamini
	Annotation Cluster 68	Enrichment Score: 1.48	G	Count	P_Value	Benjamini
☐	UP_KEYWORDS	HDL	RT	7	2.5E-4	1.0E-3
☐	UP_KEYWORDS	Lipid transport	RT	12	3.1E-4	1.2E-3
☐	GOTERM_CC_DIRECT	chylomicron	RT	5	3.0E-3	3.3E-2
☐	GOTERM_CC_DIRECT	very-low-density lipoprotein particle	RT	6	3.5E-3	3.7E-2
☐	GOTERM_MF_DIRECT	phosphatidylcholine-sterol O-acyltransferase activator activity	RT	4	6.4E-3	1.2E-1
☐	GOTERM_CC_DIRECT	high-density lipoprotein particle	RT	6	7.1E-3	7.1E-2
☐	GOTERM_CC_DIRECT	spherical high-density lipoprotein particle	RT	4	1.1E-2	9.9E-2
☐	GOTERM_BP_DIRECT	neuron projection regeneration	RT	4	1.2E-2	4.2E-1
☐	GOTERM_BP_DIRECT	phospholipid efflux	RT	5	1.8E-2	5.3E-1
☐	GOTERM_BP_DIRECT	positive regulation of cholesterol esterification	RT	4	2.0E-2	5.4E-1
☐	GOTERM_MF_DIRECT	high-density lipoprotein particle receptor binding	RT	3	2.3E-2	3.3E-1
☐	GOTERM_BP_DIRECT	negative regulation of very-low-density lipoprotein particle remodeling	RT	3	2.3E-2	5.9E-1
☐	GOTERM_BP_DIRECT	high-density lipoprotein particle assembly	RT	4	3.0E-2	6.6E-1
☐	GOTERM_BP_DIRECT	lipoprotein metabolic process	RT	5	4.1E-2	7.5E-1
☐	GOTERM_BP_DIRECT	triglyceride catabolic process	RT	5	5.1E-2	8.0E-1
☐	UP_KEYWORDS	VLDL	RT	3	5.3E-2	1.3E-1
☐	UP_KEYWORDS	Chylomicron	RT	3	5.3E-2	1.3E-1
☐	INTERPRO	Apolipoprotein A1/A4/E	RT	3	5.5E-2	7.3E-1
☐	GOTERM_BP_DIRECT	cholesterol efflux	RT	6	6.1E-2	8.5E-1
☐	GOTERM_BP_DIRECT	regulation of intestinal cholesterol absorption	RT	3	6.8E-2	8.7E-1
☐	GOTERM_BP_DIRECT	reverse cholesterol transport	RT	4	7.1E-2	8.7E-1
☐	GOTERM_MF_DIRECT	cholesterol transporter activity	RT	4	8.8E-2	6.6E-1
☐	GOTERM_MF_DIRECT	cholesterol binding	RT	6	1.2E-1	7.5E-1
☐	GOTERM_MF_DIRECT	phospholipid binding	RT	8	1.2E-1	7.6E-1
☐	GOTERM_BP_DIRECT	very-low-density lipoprotein particle remodeling	RT	3	1.3E-1	9.6E-1
☐	GOTERM_MF_DIRECT	phosphatidylcholine binding	RT	4	1.5E-1	8.2E-1
☐	GOTERM_BP_DIRECT	cholesterol biosynthetic process	RT	5	1.7E-1	9.8E-1
☐	GOTERM_BP_DIRECT	triglyceride homeostasis	RT	4	1.7E-1	9.8E-1
☐	GOTERM_BP_DIRECT	cholesterol metabolic process	RT	6	1.9E-1	9.9E-1
☐	GOTERM_BP_DIRECT	high-density lipoprotein particle remodeling	RT	3	2.3E-1	9.9E-1
☐	GOTERM_BP_DIRECT	cholesterol homeostasis	RT	7	2.5E-1	9.9E-1
☐	GOTERM_BP_DIRECT	lipid transport	RT	6	3.0E-1	1.0E0
	Annotation Cluster 69	Enrichment Score: 1.47	G	Count	P_Value	Benjamini
☐	UP_KEYWORDS	Actin capping	RT	7	3.8E-5	1.8E-4
☐	GOTERM_BP_DIRECT	actin filament severing	RT	4	1.2E-2	4.2E-1
☐	GOTERM_BP_DIRECT	barbed-end actin filament capping	RT	5	1.3E-2	4.4E-1
☐	SMART	GEL	RT	4	1.4E-2	2.1E-1
☐	INTERPRO	Villin/Gelsolin	RT	4	2.2E-2	4.6E-1
☐	GOTERM_BP_DIRECT	actin nucleation	RT	3	6.8E-2	8.7E-1
☐	UP_SEQ_FEATURE	repeat:Gelsolin-like 2	RT	4	7.0E-2	9.9E-1
☐	UP_SEQ_FEATURE	repeat:Gelsolin-like 3	RT	4	7.0E-2	9.9E-1
☐	UP_SEQ_FEATURE	repeat:Gelsolin-like 1	RT	4	7.0E-2	9.9E-1
☐	INTERPRO	Gelsolin domain	RT	4	9.8E-2	8.9E-1
☐	UP_SEQ_FEATURE	repeat:Gelsolin-like 6	RT	3	1.2E-1	1.0E0
☐	UP_SEQ_FEATURE	region of interest:Polyphosphoinositide binding	RT	3	1.2E-1	1.0E0
☐	UP_SEQ_FEATURE	repeat:Gelsolin-like 5	RT	3	2.0E-1	1.0E0
☐	UP_SEQ_FEATURE	repeat:Gelsolin-like 4	RT	3	2.0E-1	1.0E0
	Annotation Cluster 70	Enrichment Score: 1.46	G	Count	P_Value	Benjamini
☐	INTERPRO	Peptidase M16, N-terminal	RT	4	1.4E-2	3.7E-1
☐	INTERPRO	Metalloenzyme, LuxS/M16 peptidase-like	RT	4	1.4E-2	3.7E-1

	Category	Term		Count	P_Value	Benjamini
Annotation Cluster 1		Enrichment Score: 57.69	G	Count	P_Value	Benjamini
	INTERPRO	Peptidase M16 domain	RT	4	1.4E-2	3.7E-1
	INTERPRO	Peptidase M16, C-terminal domain	RT	4	1.4E-2	3.7E-1
	INTERPRO	Peptidase M16, zinc-binding site	RT	3	5.5E-2	7.3E-1
	GOTERM_MF_DIRECT	metalloendopeptidase activity	RT	9	6.9E-1	1.0E0
Annotation Cluster 71		Enrichment Score: 1.45	G	Count	P_Value	Benjamini
	UP_KEYWORDS	Metalloprotease inhibitor	RT	4	2.0E-2	5.5E-2
	UP_KEYWORDS	Metalloenzyme inhibitor	RT	4	2.0E-2	5.5E-2
	GOTERM_MF_DIRECT	metalloendopeptidase inhibitor activity	RT	4	1.1E-1	7.3E-1
Annotation Cluster 72		Enrichment Score: 1.45	G	Count	P_Value	Benjamini
	GOTERM_BP_DIRECT	fibrinolysis	RT	8	9.7E-5	9.4E-3
	INTERPRO	Kringle-like fold	RT	9	7.4E-4	4.3E-2
	SMART	KR	RT	4	6.6E-2	6.0E-1
	UP_KEYWORDS	Kringle	RT	4	9.3E-2	2.1E-1
	INTERPRO	Kringle	RT	4	9.8E-2	8.9E-1
	INTERPRO	Kringle, conserved site	RT	3	2.8E-1	1.0E0
	INTERPRO	Peptidase S1A, chymotrypsin-type	RT	10	4.7E-1	1.0E0
	INTERPRO	Peptidase S1, trypsin family, active site	RT	9	4.8E-1	1.0E0
Annotation Cluster 73		Enrichment Score: 1.38	G	Count	P_Value	Benjamini
	GOTERM_BP_DIRECT	chaperone mediated protein folding independent of cofactor	RT	5	3.5E-3	1.9E-1
	GOTERM_CC_DIRECT	prefoldin complex	RT	3	1.2E-1	5.2E-1
	INTERPRO	Prefoldin	RT	4	1.7E-1	9.7E-1
Annotation Cluster 74		Enrichment Score: 1.36	G	Count	P_Value	Benjamini
	GOTERM_MF_DIRECT	protein heterodimerization activity	RT	23	3.4E-4	1.1E-2
	UP_KEYWORDS	Nucleosome core	RT	17	2.7E-3	9.0E-3
	UP_KEYWORDS	Chromosome	RT	28	7.7E-3	2.4E-2
	UP_KEYWORDS	Citrullination	RT	10	8.9E-3	2.6E-2
	GOTERM_CC_DIRECT	nucleosome	RT	14	1.5E-2	1.3E-1
	INTERPRO	Histone core	RT	14	1.5E-2	3.7E-1
	SMART	H3	RT	5	2.5E-2	3.2E-1
	INTERPRO	Histone H3	RT	5	4.3E-2	6.6E-1
	INTERPRO	Histone-fold	RT	16	4.7E-2	6.8E-1
	KEGG_PATHWAY	Systemic lupus erythematosus	RT	23	2.0E-1	5.1E-1
	GOTERM_MF_DIRECT	nucleosomal DNA binding	RT	5	2.2E-1	9.2E-1
	KEGG_PATHWAY	Alcoholism	RT	26	2.7E-1	6.1E-1
	GOTERM_BP_DIRECT	nucleosome assembly	RT	11	5.7E-1	1.0E0
	KEGG_PATHWAY	Transcriptional misregulation in cancer	RT	14	9.0E-1	9.7E-1
	UP_KEYWORDS	DNA-binding	RT	33	1.0E0	1.0E0
Annotation Cluster 75		Enrichment Score: 1.33	G	Count	P_Value	Benjamini
	GOTERM_MF_DIRECT	chondroitin sulfate binding	RT	4	6.4E-3	1.2E-1
	SMART	ANX	RT	5	6.9E-3	1.2E-1
	UP_KEYWORDS	Calcium/phospholipid-binding	RT	5	8.1E-3	2.5E-2
	UP_KEYWORDS	Annexin	RT	5	1.1E-2	3.3E-2
	INTERPRO	Annexin repeat, conserved site	RT	5	1.2E-2	3.4E-1
	INTERPRO	Annexin repeat	RT	5	1.2E-2	3.4E-1
	INTERPRO	Annexin	RT	5	1.2E-2	3.4E-1
	GOTERM_MF_DIRECT	calcium-dependent protein binding	RT	5	5.0E-2	4.9E-1
	UP_SEQ_FEATURE	repeat:Annexin 4	RT	5	2.5E-1	1.0E0
	UP_SEQ_FEATURE	repeat:Annexin 2	RT	5	2.5E-1	1.0E0
	UP_SEQ_FEATURE	repeat:Annexin 3	RT	5	2.5E-1	1.0E0
	UP_SEQ_FEATURE	repeat:Annexin 1	RT	5	2.5E-1	1.0E0
	GOTERM_BP_DIRECT	calcium ion transmembrane transport	RT	5	3.3E-1	1.0E0
	GOTERM_MF_DIRECT	calcium-dependent phospholipid binding	RT	5	5.3E-1	1.0E0
Annotation Cluster 76		Enrichment Score: 1.32	G	Count	P_Value	Benjamini

Annotation Cluster 1	Enrichment Score: 57.69		Count	P_Value	Benjamini
INTERPRO	Lipocalin/cytosolic fatty-acid binding protein domain	RT	8	2.0E-2	4.4E-1
INTERPRO	Calycin	RT	8	2.7E-2	5.2E-1
INTERPRO	Calycin-like	RT	8	3.5E-2	5.9E-1
GOTERM_MF_DIRECT	small molecule binding	RT	4	4.1E-2	4.4E-1
INTERPRO	Lipocalin	RT	4	4.1E-2	6.5E-1
INTERPRO	Lipocalin conserved site	RT	3	4.0E-1	1.0E0
Annotation Cluster 77	Enrichment Score: 1.31		Count	P_Value	Benjamini
UP_KEYWORDS	Steroid metabolism	RT	8	9.2E-3	2.7E-2
UP_KEYWORDS	Sterol metabolism	RT	7	1.4E-2	3.9E-2
UP_KEYWORDS	Cholesterol metabolism	RT	6	4.3E-2	1.1E-1
UP_KEYWORDS	Steroid biosynthesis	RT	4	1.7E-1	3.3E-1
UP_KEYWORDS	Sterol biosynthesis	RT	3	3.0E-1	5.2E-1
Annotation Cluster 78	Enrichment Score: 1.31		Count	P_Value	Benjamini
UP_KEYWORDS	Serine esterase	RT	6	5.5E-4	2.0E-3
UP_SEQ_FEATURE	active site:Acyl-ester intermediate	RT	4	1.7E-1	1.0E0
INTERPRO	Carboxylesterase type B, active site	RT	3	1.9E-1	9.8E-1
INTERPRO	Carboxylesterase, type B	RT	3	3.1E-1	1.0E0
Annotation Cluster 79	Enrichment Score: 1.29		Count	P_Value	Benjamini
UP_KEYWORDS	Dynein	RT	6	3.3E-3	1.1E-2
GOTERM_MF_DIRECT	motor activity	RT	9	4.2E-2	4.4E-1
UP_KEYWORDS	Motor protein	RT	12	6.6E-2	1.5E-1
GOTERM_CC_DIRECT	dynein complex	RT	5	9.1E-2	4.4E-1
GOTERM_CC_DIRECT	cytoplasmic dynein complex	RT	3	4.1E-1	8.8E-1
Annotation Cluster 80	Enrichment Score: 1.27		Count	P_Value	Benjamini
INTERPRO	Thiolase-like	RT	5	6.0E-3	2.1E-1
INTERPRO	Thiolase	RT	3	5.5E-2	7.3E-1
INTERPRO	Thiolase, active site	RT	3	5.5E-2	7.3E-1
INTERPRO	Thiolase, acyl-enzyme intermediate active site	RT	3	5.5E-2	7.3E-1
GOTERM_BP_DIRECT	fatty acid beta-oxidation	RT	7	6.5E-2	8.6E-1
INTERPRO	Thiolase, C-terminal	RT	3	7.9E-2	8.4E-1
INTERPRO	Thiolase, conserved site	RT	3	7.9E-2	8.4E-1
INTERPRO	Thiolase, N-terminal	RT	3	7.9E-2	8.4E-1
UP_SEQ_FEATURE	active site:Acyl-thioester intermediate	RT	3	1.2E-1	1.0E0
Annotation Cluster 81	Enrichment Score: 1.27		Count	P_Value	Benjamini
UP_KEYWORDS	Aminotransferase	RT	6	8.4E-3	2.5E-2
INTERPRO	Aspartate/other aminotransferase	RT	3	1.9E-2	4.2E-1
UP_KEYWORDS	Pyridoxal phosphate	RT	10	1.9E-2	5.3E-2
GOTERM_MF_DIRECT	L-aspartate:2-oxoglutarate aminotransferase activity	RT	3	2.3E-2	3.3E-1
GOTERM_MF_DIRECT	pyridoxal phosphate binding	RT	11	2.7E-2	3.5E-1
GOTERM_BP_DIRECT	aspartate biosynthetic process	RT	3	6.8E-2	8.7E-1
INTERPRO	Pyridoxal phosphate-dependent transferase	RT	7	1.5E-1	9.6E-1
INTERPRO	Pyridoxal phosphate-dependent transferase, major region, subdomain 1	RT	7	1.5E-1	9.6E-1
INTERPRO	Aminotransferase, class I/classII	RT	4	1.7E-1	9.7E-1
INTERPRO	Pyridoxal phosphate-dependent transferase, major region, subdomain 2	RT	5	4.3E-1	1.0E0
Annotation Cluster 82	Enrichment Score: 1.26		Count	P_Value	Benjamini
GOTERM_MF_DIRECT	lipopolysaccharide binding	RT	7	2.8E-3	6.5E-2
GOTERM_CC_DIRECT	lipopolysaccharide receptor complex	RT	3	9.0E-2	4.4E-1
GOTERM_BP_DIRECT	positive regulation of interferon-gamma production	RT	4	6.7E-1	1.0E0
Annotation Cluster 83	Enrichment Score: 1.26		Count	P_Value	Benjamini
GOTERM_CC_DIRECT	vacuolar membrane	RT	5	1.2E-2	1.0E-1
INTERPRO	NSF attachment protein	RT	3	1.9E-2	4.2E-1
GOTERM_MF_DIRECT	soluble NSF attachment protein activity	RT	3	2.3E-2	3.3E-1

					Count	P_Value	Benjamini
Annotation Cluster 1	Enrichment Score: 57.69	G			Count	P_Value	Benjamini
GOTERM_BP_DIRECT	membrane fusion	RT			6	3.6E-2	7.2E-1
GOTERM_CC_DIRECT	synaptobrevin 2-SNAP-25-syntaxin-1a complex	RT			3	4.0E-2	2.7E-1
GOTERM_CC_DIRECT	SNARE complex	RT			6	3.9E-1	8.7E-1
GOTERM_MF_DIRECT	syntaxin binding	RT			5	5.7E-1	1.0E0
Annotation Cluster 84	Enrichment Score: 1.25	G			Count	P_Value	Benjamini
INTERPRO	Bactericidal permeability-increasing protein, alpha/beta domain	RT			5	2.8E-2	5.3E-1
INTERPRO	Lipid-binding serum glycoprotein, N-terminal	RT			5	2.8E-2	5.3E-1
SMART	BPI1	RT			3	7.8E-2	6.3E-1
INTERPRO	Lipid-binding serum glycoprotein, C-terminal	RT			3	1.6E-1	9.6E-1
Annotation Cluster 85	Enrichment Score: 1.24	G			Count	P_Value	Benjamini
UP_KEYWORDS	2Fe-2S	RT			7	9.6E-4	3.4E-3
GOTERM_MF_DIRECT	2 iron, 2 sulfur cluster binding	RT			7	1.4E-2	2.3E-1
INTERPRO	Beta-grasp domain	RT			4	1.2E-1	9.2E-1
INTERPRO	2Fe-2S ferredoxin-type domain	RT			3	1.3E-1	9.4E-1
UP_SEQ_FEATURE	domain:2Fe-2S ferredoxin-type	RT			3	3.8E-1	1.0E0
UP_SEQ_FEATURE	metal ion-binding site:Iron-sulfur (2Fe-2S)	RT			4	4.3E-1	1.0E0
Annotation Cluster 86	Enrichment Score: 1.23	G			Count	P_Value	Benjamini
UP_KEYWORDS	Cell division	RT			23	3.5E-3	1.1E-2
UP_KEYWORDS	Cell cycle	RT			31	4.8E-3	1.5E-2
GOTERM_BP_DIRECT	cell division	RT			20	8.6E-2	9.1E-1
GOTERM_BP_DIRECT	mitotic nuclear division	RT			13	2.4E-1	9.9E-1
UP_KEYWORDS	Mitosis	RT			11	2.9E-1	5.2E-1
GOTERM_CC_DIRECT	spindle	RT			7	4.3E-1	8.9E-1
Annotation Cluster 87	Enrichment Score: 1.23	G			Count	P_Value	Benjamini
GOTERM_BP_DIRECT	prostaglandin biosynthetic process	RT			6	8.5E-3	3.4E-1
UP_KEYWORDS	Prostaglandin biosynthesis	RT			4	3.9E-2	9.7E-2
UP_KEYWORDS	Prostaglandin metabolism	RT			4	5.0E-2	1.2E-1
KEGG_PATHWAY	Arachidonic acid metabolism	RT			7	7.6E-1	9.1E-1
Annotation Cluster 88	Enrichment Score: 1.21	G			Count	P_Value	Benjamini
INTERPRO	Adenylate kinase	RT			4	3.1E-2	5.5E-1
GOTERM_MF_DIRECT	adenylate kinase activity	RT			4	4.1E-2	4.4E-1
GOTERM_BP_DIRECT	ADP biosynthetic process	RT			3	4.4E-2	7.6E-1
UP_SEQ_FEATURE	binding site:AMP	RT			6	9.4E-2	1.0E0
GOTERM_BP_DIRECT	AMP metabolic process	RT			3	9.7E-2	9.2E-1
UP_SEQ_FEATURE	nucleotide phosphate-binding region:AMP	RT			5	1.1E-1	1.0E0
Annotation Cluster 89	Enrichment Score: 1.19	G			Count	P_Value	Benjamini
GOTERM_BP_DIRECT	acetyl-CoA biosynthetic process from pyruvate	RT			3	4.4E-2	7.6E-1
GOTERM_CC_DIRECT	pyruvate dehydrogenase complex	RT			3	6.4E-2	3.6E-1
GOTERM_MF_DIRECT	pyruvate dehydrogenase (NAD+) activity	RT			3	9.6E-2	6.9E-1
Annotation Cluster 90	Enrichment Score: 1.19	G			Count	P_Value	Benjamini
UP_KEYWORDS	Translocation	RT			9	7.2E-3	2.2E-2
UP_KEYWORDS	Nuclear pore complex	RT			5	1.1E-2	3.3E-2
UP_KEYWORDS	mRNA transport	RT			6	1.5E-1	3.1E-1
GOTERM_CC_DIRECT	nuclear pore	RT			6	2.5E-1	7.3E-1
GOTERM_BP_DIRECT	mRNA transport	RT			4	3.7E-1	1.0E0
Annotation Cluster 91	Enrichment Score: 1.17	G			Count	P_Value	Benjamini
INTERPRO	Ubiquitin-conjugating enzyme, E2	RT			11	4.1E-4	2.8E-2
INTERPRO	Ubiquitin-conjugating enzyme, active site	RT			8	1.7E-3	8.4E-2
GOTERM_MF_DIRECT	ubiquitin conjugating enzyme activity	RT			9	2.0E-3	5.2E-2
INTERPRO	Ubiquitin-conjugating enzyme/RWD-like	RT			11	5.9E-3	2.1E-1
UP_SEQ_FEATURE	active site:Glycyl thioester intermediate	RT			10	4.2E-2	9.7E-1
GOTERM_BP_DIRECT	postreplication repair	RT			4	7.1E-2	8.7E-1
GOTERM_BP_DIRECT	protein K63-linked ubiquitination	RT			6	1.0E-1	9.3E-1

	Category	Term			Count	P_Value	Benjamini
	Annotation Cluster 1	Enrichment Score: 57.69	G		Count	P_Value	Benjamini
☐	GOTERM_BP_DIRECT	protein K48-linked ubiquitination	RT		8	1.1E-1	9.4E-1
☐	GOTERM_BP_DIRECT	protein K11-linked ubiquitination	RT		4	3.5E-1	1.0E0
☐	KEGG_PATHWAY	Ubiquitin mediated proteolysis	RT		13	7.4E-1	9.1E-1
☐	GOTERM_MF_DIRECT	ubiquitin-protein transferase activity	RT		15	8.1E-1	1.0E0
☐	GOTERM_BP_DIRECT	protein polyubiquitination	RT		6	8.5E-1	1.0E0
☐	GOTERM_MF_DIRECT	ubiquitin protein ligase activity	RT		11	8.6E-1	1.0E0
☐	GOTERM_BP_DIRECT	protein autoubiquitination	RT		3	9.4E-1	1.0E0
	Annotation Cluster 92	Enrichment Score: 1.16	G		Count	P_Value	Benjamini
☐	GOTERM_MF_DIRECT	chaperone binding	RT		9	1.2E-3	3.3E-2
☐	SMART	DnaJ	RT		8	4.8E-2	4.9E-1
☐	INTERPRO	DnaJ domain	RT		8	1.1E-1	9.2E-1
☐	INTERPRO	DnaJ domain, conserved site	RT		5	1.5E-1	9.6E-1
☐	INTERPRO	HSP40/DnaJ peptide-binding	RT		3	1.6E-1	9.6E-1
☐	INTERPRO	Chaperone DnaJ, C-terminal	RT		3	1.6E-1	9.6E-1
☐	UP_SEQ_FEATURE	domain:J	RT		8	3.2E-1	1.0E0
	Annotation Cluster 93	Enrichment Score: 1.14	G		Count	P_Value	Benjamini
☐	UP_KEYWORDS	Signalosome	RT		3	3.4E-2	8.6E-2
☐	GOTERM_CC_DIRECT	COP9 signalosome	RT		7	4.8E-2	3.0E-1
☐	GOTERM_BP_DIRECT	cullin deneddylation	RT		3	2.3E-1	9.9E-1
	Annotation Cluster 94	Enrichment Score: 1.14	G		Count	P_Value	Benjamini
☐	INTERPRO	Ubiquitin	RT		12	5.3E-3	1.9E-1
☐	INTERPRO	Ubiquitin conserved site	RT		5	8.8E-3	2.7E-1
☐	INTERPRO	Ubiquitin subgroup	RT		5	1.7E-2	3.9E-1
☐	SMART	UBQ	RT		8	1.8E-2	2.4E-1
☐	UP_SEQ_FEATURE	chain:60S ribosomal protein L40	RT		3	1.2E-1	1.0E0
☐	UP_SEQ_FEATURE	chain:Ubiquitin	RT		4	1.7E-1	1.0E0
☐	UP_SEQ_FEATURE	site:Essential for function	RT		4	1.7E-1	1.0E0
☐	UP_SEQ_FEATURE	binding site:Activating enzyme	RT		4	2.4E-1	1.0E0
☐	UP_SEQ_FEATURE	cross-link:Glycyl lysine isopeptide (Gly-Lys) (interchain with K-? in acceptor proteins)	RT		5	5.1E-1	1.0E0
☐	UP_SEQ_FEATURE	domain:Ubiquitin-like	RT		6	6.1E-1	1.0E0
	Annotation Cluster 95	Enrichment Score: 1.13	G		Count	P_Value	Benjamini
☐	UP_SEQ_FEATURE	site:Transition state stabilizer	RT		7	4.5E-3	4.2E-1
☐	KEGG_PATHWAY	beta-Alanine metabolism	RT		9	1.9E-2	1.1E-1
☐	GOTERM_MF_DIRECT	aldehyde dehydrogenase (NAD) activity	RT		5	3.2E-2	3.8E-1
☐	INTERPRO	Aldehyde dehydrogenase domain	RT		5	5.2E-2	7.1E-1
☐	INTERPRO	Aldehyde dehydrogenase, N-terminal	RT		5	5.2E-2	7.1E-1
☐	INTERPRO	Aldehyde dehydrogenase, C-terminal	RT		5	5.2E-2	7.1E-1
☐	INTERPRO	Aldehyde/histidinol dehydrogenase	RT		5	5.2E-2	7.1E-1
☐	INTERPRO	Aldehyde dehydrogenase, conserved site	RT		4	8.2E-2	8.5E-1
☐	KEGG_PATHWAY	Tryptophan metabolism	RT		9	9.1E-2	3.4E-1
☐	KEGG_PATHWAY	Lysine degradation	RT		9	1.8E-1	4.9E-1
☐	KEGG_PATHWAY	Histidine metabolism	RT		5	1.8E-1	4.9E-1
☐	KEGG_PATHWAY	Glycerolipid metabolism	RT		7	6.3E-1	8.7E-1
☐	KEGG_PATHWAY	Ascorbate and aldarate metabolism	RT		3	6.5E-1	8.8E-1
	Annotation Cluster 96	Enrichment Score: 1.13	G		Count	P_Value	Benjamini
☐	INTERPRO	Profilin, chordates	RT		3	1.9E-2	4.2E-1
☐	SMART	PROF	RT		3	2.6E-2	3.1E-1
☐	INTERPRO	Profilin	RT		3	3.5E-2	5.9E-1
☐	GOTERM_BP_DIRECT	positive regulation of actin filament bundle assembly	RT		4	4.1E-2	7.5E-1
☐	GOTERM_BP_DIRECT	regulation of actin filament polymerization	RT		3	3.0E-1	1.0E0
☐	GOTERM_BP_DIRECT	actin cytoskeleton organization	RT		6	8.6E-1	1.0E0
	Annotation Cluster 97	Enrichment Score: 1.09	G		Count	P_Value	Benjamini
☐	INTERPRO	Glutathione S-transferase, C-terminal-like	RT		9	1.1E-2	3.3E-1

Annotation Cluster 1	Enrichment Score: 57.69		Count	P_Value	Benjamini
GOTERM_BP_DIRECT	glutathione metabolic process	RT	8	1.3E-2	4.4E-1
INTERPRO	Glutathione S-transferase, N-terminal	RT	7	2.2E-2	4.6E-1
UP_SEQ_FEATURE	domain:GST C-terminal	RT	8	5.3E-2	9.8E-1
GOTERM_MF_DIRECT	glutathione transferase activity	RT	7	5.6E-2	5.2E-1
UP_SEQ_FEATURE	domain:GST N-terminal	RT	5	7.1E-2	9.9E-1
INTERPRO	Glutathione S-transferase, C-terminal	RT	5	9.7E-2	8.9E-1
KEGG_PATHWAY	Drug metabolism - cytochrome P450	RT	7	5.5E-1	8.4E-1
KEGG_PATHWAY	Metabolism of xenobiotics by cytochrome P450	RT	7	5.7E-1	8.4E-1
KEGG_PATHWAY	Chemical carcinogenesis	RT	8	5.8E-1	8.4E-1
Annotation Cluster 98	Enrichment Score: 1.09		Count	P_Value	Benjamini
INTERPRO	Phosphoglycerate mutase 1	RT	3	1.9E-2	4.2E-1
GOTERM_MF_DIRECT	2,3-bisphosphoglycerate-dependent phosphoglycerate mutase activity	RT	3	2.3E-2	3.3E-1
GOTERM_MF_DIRECT	bisphosphoglycerate mutase activity	RT	3	2.3E-2	3.3E-1
GOTERM_MF_DIRECT	bisphosphoglycerate 2-phosphatase activity	RT	3	4.3E-2	4.5E-1
GOTERM_BP_DIRECT	regulation of pentose-phosphate shunt	RT	3	4.4E-2	7.6E-1
SMART	SM00855	RT	3	1.2E-1	7.2E-1
INTERPRO	Phosphoglycerate/bisphosphoglycerate mutase, active site	RT	3	1.3E-1	9.4E-1
INTERPRO	Histidine phosphatase superfamily, clade-1	RT	3	2.2E-1	9.9E-1
UP_SEQ_FEATURE	active site:Tele-phosphohistidine intermediate	RT	5	3.5E-1	1.0E0
UP_SEQ_FEATURE	site:Interaction with carboxyl group of phosphoglycerates	RT	3	5.4E-1	1.0E0
Annotation Cluster 99	Enrichment Score: 1.07		Count	P_Value	Benjamini
INTERPRO	EF-Hand 1, calcium-binding site	RT	25	1.5E-3	7.6E-2
SMART	EFh	RT	19	4.4E-3	9.1E-2
INTERPRO	EF-hand domain	RT	28	5.0E-3	2.0E-1
INTERPRO	EF-hand-like domain	RT	31	1.4E-2	3.6E-1
GOTERM_MF_DIRECT	calcium ion binding	RT	63	7.6E-2	6.2E-1
UP_SEQ_FEATURE	domain:EF-hand 1	RT	24	1.2E-1	1.0E0
UP_SEQ_FEATURE	domain:EF-hand 2	RT	24	1.2E-1	1.0E0
UP_SEQ_FEATURE	calcium-binding region:1	RT	18	1.6E-1	1.0E0
UP_SEQ_FEATURE	domain:EF-hand 5	RT	4	1.7E-1	1.0E0
UP_SEQ_FEATURE	calcium-binding region:2	RT	15	2.8E-1	1.0E0
UP_SEQ_FEATURE	calcium-binding region:5	RT	3	2.9E-1	1.0E0
UP_SEQ_FEATURE	calcium-binding region:4	RT	5	3.0E-1	1.0E0
UP_SEQ_FEATURE	domain:EF-hand 3	RT	11	5.7E-1	1.0E0
UP_SEQ_FEATURE	domain:EF-hand 4	RT	8	7.1E-1	1.0E0
UP_SEQ_FEATURE	calcium-binding region:3	RT	6	7.5E-1	1.0E0
Annotation Cluster 100	Enrichment Score: 1.06		Count	P_Value	Benjamini
GOTERM_BP_DIRECT	peptide catabolic process	RT	7	5.3E-3	2.5E-1
GOTERM_MF_DIRECT	metalloaminopeptidase activity	RT	5	5.0E-2	4.9E-1
GOTERM_MF_DIRECT	peptide binding	RT	7	8.2E-2	6.4E-1
INTERPRO	Peptidase M1, alanine aminopeptidase/leukotriene A4 hydrolase	RT	3	1.9E-1	9.8E-1
INTERPRO	Peptidase M1, membrane alanine aminopeptidase, N-terminal	RT	3	2.2E-1	9.9E-1
UP_SEQ_FEATURE	metal ion-binding site:Zinc; catalytic	RT	9	4.5E-1	1.0E0
Annotation Cluster 101	Enrichment Score: 1.05		Count	P_Value	Benjamini
GOTERM_MF_DIRECT	lipopolysaccharide binding	RT	7	2.8E-3	6.5E-2
GOTERM_BP_DIRECT	cellular response to lipoteichoic acid	RT	3	9.7E-2	9.2E-1
GOTERM_BP_DIRECT	lipopolysaccharide-mediated signaling pathway	RT	5	2.5E-1	9.9E-1
KEGG_PATHWAY	NF-kappa B signaling pathway	RT	7	9.1E-1	9.7E-1
Annotation Cluster 102	Enrichment Score: 1.04		Count	P_Value	Benjamini
INTERPRO	Peptidase S8/S53 domain	RT	4	3.1E-2	5.5E-1
INTERPRO	Peptidase S8, subtilisin, Ser-active site	RT	3	1.3E-1	9.4E-1

	Category	Term				Count	P_Value	Benjamini
	Annotation Cluster 1	Enrichment Score: 57.69	G			Count	P_Value	Benjamini
☐	INTERPRO	Peptidase S8, subtilisin-related	RT			3	1.3E-1	9.4E-1
☐	INTERPRO	Peptidase S8, subtilisin, His-active site	RT			3	1.3E-1	9.4E-1
	Annotation Cluster 103	Enrichment Score: 1.04	G			Count	P_Value	Benjamini
☐	INTERPRO	Insulin family	RT			3	5.5E-2	7.3E-1
☐	SMART	IlGF	RT			3	7.8E-2	6.3E-1
☐	INTERPRO	Insulin-like	RT			3	1.0E-1	9.0E-1
☐	INTERPRO	Insulin, conserved site	RT			3	1.0E-1	9.0E-1
☐	GOTERM_MF_DIRECT	hormone activity	RT			9	1.4E-1	8.1E-1
	Annotation Cluster 104	Enrichment Score: 1.03	G			Count	P_Value	Benjamini
☐	UP_KEYWORDS	Sushi	RT			8	2.5E-3	8.7E-3
☐	UP_SEQ_FEATURE	domain:Sushi 1	RT			8	9.3E-2	1.0E0
☐	UP_SEQ_FEATURE	domain:Sushi 2	RT			8	1.2E-1	1.0E0
☐	INTERPRO	Sushi/SCR/CCP	RT			8	1.2E-1	9.3E-1
☐	SMART	CCP	RT			7	1.2E-1	7.2E-1
☐	UP_SEQ_FEATURE	domain:Sushi 3	RT			5	2.5E-1	1.0E0
☐	UP_SEQ_FEATURE	domain:Sushi 4	RT			3	6.1E-1	1.0E0
	Annotation Cluster 105	Enrichment Score: 1.03	G			Count	P_Value	Benjamini
☐	INTERPRO	Somatomedin B domain	RT			4	4.1E-2	6.5E-1
☐	SMART	SO	RT			3	7.8E-2	6.3E-1
☐	GOTERM_MF_DIRECT	polysaccharide binding	RT			4	8.8E-2	6.6E-1
☐	GOTERM_MF_DIRECT	scavenger receptor activity	RT			7	2.8E-1	9.5E-1
	Annotation Cluster 106	Enrichment Score: 1.02	G			Count	P_Value	Benjamini
☐	GOTERM_BP_DIRECT	nucleoside diphosphate phosphorylation	RT			5	1.9E-3	1.2E-1
☐	GOTERM_BP_DIRECT	nucleoside triphosphate biosynthetic process	RT			5	1.3E-2	4.4E-1
☐	GOTERM_MF_DIRECT	nucleoside diphosphate kinase activity	RT			5	6.1E-2	5.5E-1
☐	UP_SEQ_FEATURE	active site:Pros-phosphohistidine intermediate	RT			3	1.2E-1	1.0E0
☐	SMART	NDK	RT			3	1.2E-1	7.2E-1
☐	INTERPRO	Nucleoside diphosphate kinase	RT			3	1.6E-1	9.6E-1
☐	GOTERM_BP_DIRECT	CTP biosynthetic process	RT			3	1.9E-1	9.9E-1
☐	GOTERM_BP_DIRECT	UTP biosynthetic process	RT			3	1.9E-1	9.9E-1
☐	UP_KEYWORDS	Nucleotide metabolism	RT			3	2.1E-1	4.1E-1
☐	GOTERM_BP_DIRECT	GTP biosynthetic process	RT			3	2.6E-1	9.9E-1
☐	KEGG_PATHWAY	Pyrimidine metabolism	RT			8	8.8E-1	9.7E-1
	Annotation Cluster 107	Enrichment Score: 1	G			Count	P_Value	Benjamini
☐	GOTERM_CC_DIRECT	nucleosome	RT			14	1.5E-2	1.3E-1
☐	INTERPRO	Histone core	RT			14	1.5E-2	3.7E-1
☐	SMART	H2A	RT			5	9.9E-2	6.8E-1
☐	INTERPRO	Histone H2A	RT			5	1.5E-1	9.6E-1
☐	GOTERM_BP_DIRECT	chromatin silencing	RT			5	3.1E-1	1.0E0
☐	GOTERM_CC_DIRECT	nuclear chromatin	RT			8	9.8E-1	1.0E0
	Annotation Cluster 108	Enrichment Score: 0.99	G			Count	P_Value	Benjamini
☐	GOTERM_BP_DIRECT	terminal web assembly	RT			3	2.3E-2	5.9E-1
☐	GOTERM_BP_DIRECT	intestinal D-glucose absorption	RT			3	4.4E-2	7.6E-1
☐	GOTERM_BP_DIRECT	regulation of microvillus length	RT			3	9.7E-2	9.2E-1
☐	GOTERM_BP_DIRECT	positive regulation of establishment of protein localization to plasma membrane	RT			5	2.1E-1	9.9E-1
☐	GOTERM_BP_DIRECT	positive regulation of multicellular organism growth	RT			4	5.4E-1	1.0E0
	Annotation Cluster 109	Enrichment Score: 0.97	G			Count	P_Value	Benjamini
☐	INTERPRO	Alkaline-phosphatase-like, core domain	RT			7	3.0E-2	5.6E-1
☐	INTERPRO	Alkaline phosphatase-like, alpha/beta/alpha	RT			7	3.0E-2	5.6E-1
☐	UP_SEQ_FEATURE	metal ion-binding site:Calcium	RT			8	9.3E-2	1.0E0
☐	INTERPRO	Ectonucleotide pyrophosphatase/phosphodiesterase family	RT			3	1.0E-1	9.0E-1
☐	UP_SEQ_FEATURE	metal ion-binding site:Calcium; via 3-oxoalanine	RT			3	1.2E-1	1.0E0

	Category	Term		Count	P_Value	Benjamini
	Annotation Cluster 1	Enrichment Score: 57.69	G	Count	P_Value	Benjamini
☐	COG_ONTOLOGY	Inorganic ion transport and metabolism	RT	4	2.1E-1	6.5E-1
☐	INTERPRO	Type I phosphodiesterase/nucleotide pyrophosphatase/phosphate transferase	RT	3	2.2E-1	9.9E-1
☐	INTERPRO	Sulfatase	RT	3	3.4E-1	1.0E0
	Annotation Cluster 110	Enrichment Score: 0.97	G	Count	P_Value	Benjamini
☐	INTERPRO	Nitrophenylphosphatase-like domain	RT	3	5.5E-2	7.3E-1
☐	INTERPRO	HAD-superfamily hydrolase, subfamily IIA	RT	3	7.9E-2	8.4E-1
☐	INTERPRO	HAD-like domain	RT	9	2.8E-1	1.0E0
	Annotation Cluster 111	Enrichment Score: 0.96	G	Count	P_Value	Benjamini
☐	GOTERM_BP_DIRECT	mitochondrion organization	RT	8	3.5E-2	7.1E-1
☐	SMART	PHB	RT	3	1.7E-1	8.2E-1
☐	INTERPRO	Band 7 protein	RT	3	2.2E-1	9.9E-1
	Annotation Cluster 112	Enrichment Score: 0.94	G	Count	P_Value	Benjamini
☐	INTERPRO	Phospholipase D/Transphosphatidylase	RT	3	1.0E-1	9.0E-1
☐	UP_SEQ_FEATURE	domain:PLD phosphodiesterase 2	RT	3	1.2E-1	1.0E0
☐	UP_SEQ_FEATURE	domain:PLD phosphodiesterase 1	RT	3	1.2E-1	1.0E0
	Annotation Cluster 113	Enrichment Score: 0.91	G	Count	P_Value	Benjamini
☐	UP_KEYWORDS	Rotamase	RT	8	5.1E-3	1.6E-2
☐	GOTERM_BP_DIRECT	protein peptidyl-prolyl isomerization	RT	8	5.6E-3	2.6E-1
☐	UP_KEYWORDS	Cyclosporin	RT	3	5.3E-2	1.3E-1
☐	GOTERM_MF_DIRECT	peptidyl-prolyl cis-trans isomerase activity	RT	8	7.3E-2	6.0E-1
☐	GOTERM_MF_DIRECT	peptide binding	RT	7	8.2E-2	6.4E-1
☐	INTERPRO	Cyclophilin-type peptidyl-prolyl cis-trans isomerase	RT	4	1.5E-1	9.6E-1
☐	INTERPRO	Cyclophilin-like peptidyl-prolyl cis-trans isomerase domain	RT	4	1.7E-1	9.7E-1
☐	GOTERM_BP_DIRECT	chaperone-mediated protein folding	RT	5	2.7E-1	1.0E0
☐	INTERPRO	Cyclophilin-type peptidyl-prolyl cis-trans isomerase, conserved site	RT	3	3.4E-1	1.0E0
☐	INTERPRO	Peptidyl-prolyl cis-trans isomerase, FKBP-type, domain	RT	3	4.0E-1	1.0E0
☐	INTERPRO	Peptidyl-prolyl cis-trans isomerase, FKBP-type	RT	3	4.9E-1	1.0E0
☐	GOTERM_MF_DIRECT	FK506 binding	RT	3	5.5E-1	1.0E0
☐	UP_SEQ_FEATURE	domain:PPIase cyclophilin-type	RT	4	7.0E-1	1.0E0
	Annotation Cluster 114	Enrichment Score: 0.87	G	Count	P_Value	Benjamini
☐	GOTERM_MF_DIRECT	actin-dependent ATPase activity	RT	5	9.0E-3	1.6E-1
☐	GOTERM_MF_DIRECT	calmodulin binding	RT	12	3.7E-2	4.1E-1
☐	UP_KEYWORDS	Motor protein	RT	12	6.6E-2	1.5E-1
☐	UP_SEQ_FEATURE	domain:IQ 1	RT	5	7.1E-2	9.9E-1
☐	UP_SEQ_FEATURE	domain:IQ 2	RT	5	7.1E-2	9.9E-1
☐	UP_KEYWORDS	Calmodulin-binding	RT	8	9.6E-2	2.1E-1
☐	SMART	IQ	RT	7	1.0E-1	6.7E-1
☐	UP_SEQ_FEATURE	domain:Myosin head-like	RT	4	2.4E-1	1.0E0
☐	UP_KEYWORDS	Myosin	RT	6	2.5E-1	4.6E-1
☐	INTERPRO	IQ motif, EF-hand binding site	RT	8	4.6E-1	1.0E0
☐	SMART	MYSc	RT	4	4.7E-1	9.9E-1
☐	INTERPRO	Myosin head, motor domain	RT	4	5.8E-1	1.0E0
☐	GOTERM_CC_DIRECT	myosin complex	RT	4	7.1E-1	9.9E-1
	Annotation Cluster 115	Enrichment Score: 0.86	G	Count	P_Value	Benjamini
☐	UP_KEYWORDS	Thioester bond	RT	3	3.4E-2	8.6E-2
☐	INTERPRO	Alpha-2-macroglobulin, conserved site	RT	3	1.0E-1	9.0E-1
☐	SMART	SM01361	RT	3	1.5E-1	7.7E-1
☐	INTERPRO	Alpha-2-macroglobulin, thiol-ester bond-forming	RT	3	1.6E-1	9.6E-1
☐	INTERPRO	A-macroglobulin complement component	RT	3	1.9E-1	9.8E-1
☐	INTERPRO	Alpha-macroglobulin, receptor-binding	RT	3	1.9E-1	9.8E-1
☐	INTERPRO	Terpenoid cyclases/protein prenyltransferase alpha-alpha toroid	RT	3	3.1E-1	1.0E0

	Category	Term			Count	P_Value	Benjamini
	Annotation Cluster 1	Enrichment Score: 57.69	G		Count	P_Value	Benjamini
	Annotation Cluster 116	Enrichment Score: 0.85	G		Count	P_Value	Benjamini
☐	INTERPRO	Heat shock protein Hsp90, conserved site	RT		3	3.5E-2	5.9E-1
☐	INTERPRO	Heat shock protein Hsp90	RT		3	5.5E-2	7.3E-1
☐	INTERPRO	Heat shock protein Hsp90, N-terminal	RT		3	5.5E-2	7.3E-1
☐	INTERPRO	Ribosomal protein S5 domain 2-type fold	RT		6	1.4E-1	9.4E-1
☐	SMART	HATPase_c	RT		3	1.5E-1	7.7E-1
☐	PIR_SUPERFAMILY	heat shock protein, HSP90/HTPG types	RT		3	1.6E-1	1.0E0
☐	INTERPRO	Histidine kinase-like ATPase, ATP-binding domain	RT		3	4.9E-1	1.0E0
☐	KEGG_PATHWAY	Prostate cancer	RT		5	9.8E-1	1.0E0
	Annotation Cluster 117	Enrichment Score: 0.85	G		Count	P_Value	Benjamini
☐	INTERPRO	HSP20-like chaperone	RT		7	1.6E-2	3.7E-1
☐	INTERPRO	CS-like domain	RT		3	3.4E-1	1.0E0
☐	UP_SEQ_FEATURE	domain:CS	RT		3	5.4E-1	1.0E0
	Annotation Cluster 118	Enrichment Score: 0.79	G		Count	P_Value	Benjamini
☐	UP_KEYWORDS	Gamma-carboxyglutamic acid	RT		5	8.1E-3	2.5E-2
☐	SMART	GLA	RT		3	2.5E-1	9.1E-1
☐	INTERPRO	Coagulation factor, subgroup, Gla domain	RT		3	2.5E-1	9.9E-1
☐	INTERPRO	Gamma-carboxyglutamic acid-rich (GLA) domain	RT		3	3.1E-1	1.0E0
☐	UP_SEQ_FEATURE	domain:Gla	RT		3	6.7E-1	1.0E0
	Annotation Cluster 119	Enrichment Score: 0.76	G		Count	P_Value	Benjamini
☐	GOTERM_MF_DIRECT	cysteine-type endopeptidase inhibitor activity	RT		6	9.1E-2	6.8E-1
☐	UP_KEYWORDS	Thiol protease inhibitor	RT		4	1.5E-1	3.0E-1
☐	INTERPRO	Proteinase inhibitor I25, cystatin	RT		4	1.9E-1	9.8E-1
☐	SMART	CY	RT		3	3.5E-1	9.7E-1
	Annotation Cluster 120	Enrichment Score: 0.69	G		Count	P_Value	Benjamini
☐	GOTERM_BP_DIRECT	positive regulation of early endosome to late endosome transport	RT		4	1.2E-2	4.2E-1
☐	GOTERM_BP_DIRECT	establishment of endothelial barrier	RT		5	3.2E-2	6.8E-1
☐	INTERPRO	Ezrin/radixin/moesin	RT		3	3.5E-2	5.9E-1
☐	INTERPRO	Ezrin/radixin/moesin, C-terminal	RT		3	3.5E-2	5.9E-1
☐	GOTERM_BP_DIRECT	positive regulation of protein localization to early endosome	RT		3	4.4E-2	7.6E-1
☐	INTERPRO	Moesin	RT		3	5.5E-2	7.3E-1
☐	PIR_SUPERFAMILY	ezrin/radixin/moesin	RT		3	1.0E-1	9.9E-1
☐	GOTERM_BP_DIRECT	positive regulation of cellular protein catabolic process	RT		3	2.3E-1	9.9E-1
☐	UP_SEQ_FEATURE	domain:FERM	RT		4	2.4E-1	1.0E0
☐	INTERPRO	Ezrin/radixin/moesin like	RT		3	2.8E-1	1.0E0
☐	INTERPRO	FERM/acyl-CoA-binding protein, 3-helical bundle	RT		6	3.8E-1	1.0E0
☐	INTERPRO	FERM conserved site	RT		3	5.4E-1	1.0E0
☐	SMART	SM01196	RT		3	5.4E-1	9.9E-1
☐	INTERPRO	FERM, N-terminal	RT		3	6.1E-1	1.0E0
☐	INTERPRO	FERM, C-terminal PH-like domain	RT		3	6.3E-1	1.0E0
☐	SMART	B41	RT		4	6.5E-1	1.0E0
☐	INTERPRO	FERM domain	RT		4	7.3E-1	1.0E0
☐	INTERPRO	FERM central domain	RT		4	7.7E-1	1.0E0
☐	INTERPRO	Band 4.1 domain	RT		4	7.7E-1	1.0E0
☐	GOTERM_CC_DIRECT	extrinsic component of membrane	RT		4	8.8E-1	1.0E0
	Annotation Cluster 121	Enrichment Score: 0.66	G		Count	P_Value	Benjamini
☐	GOTERM_MF_DIRECT	structural constituent of ribosome	RT		28	4.4E-2	4.5E-1
☐	GOTERM_MF_DIRECT	large ribosomal subunit rRNA binding	RT		3	6.8E-2	5.9E-1
☐	GOTERM_BP_DIRECT	cytoplasmic translation	RT		6	7.1E-2	8.7E-1
☐	GOTERM_CC_DIRECT	cytosolic large ribosomal subunit	RT		8	3.1E-1	8.0E-1
☐	GOTERM_CC_DIRECT	preribosome, large subunit precursor	RT		4	4.2E-1	8.9E-1
☐	UP_KEYWORDS	Ribosomal protein	RT		15	4.6E-1	7.0E-1

Annotation Cluster 1	Enrichment Score: 57.69	G	Count	P_Value	Benjamini
UP_KEYWORDS	Ribonucleoprotein	RT	18	6.0E-1	8.3E-1
KEGG_PATHWAY	Ribosome	RT	13	7.3E-1	9.1E-1
Annotation Cluster 122	Enrichment Score: 0.63	G	Count	P_Value	Benjamini
SMART	ADF	RT	3	1.5E-1	7.7E-1
INTERPRO	Actin-binding, cofilin/tropomyosin type	RT	3	1.9E-1	9.8E-1
UP_SEQ_FEATURE	domain:ADF-H	RT	3	4.6E-1	1.0E0
Annotation Cluster 123	Enrichment Score: 0.63	G	Count	P_Value	Benjamini
SMART	UBX	RT	3	1.2E-1	7.2E-1
INTERPRO	UBX	RT	3	2.8E-1	1.0E0
UP_SEQ_FEATURE	domain:UBX	RT	3	3.8E-1	1.0E0
Annotation Cluster 124	Enrichment Score: 0.62	G	Count	P_Value	Benjamini
INTERPRO	HIT-like domain	RT	3	1.6E-1	9.6E-1
UP_SEQ_FEATURE	short sequence motif:Histidine triad motif	RT	3	2.9E-1	1.0E0
UP_SEQ_FEATURE	domain:HIT	RT	3	2.9E-1	1.0E0
Annotation Cluster 125	Enrichment Score: 0.62	G	Count	P_Value	Benjamini
UP_SEQ_FEATURE	domain:EGF-like 1	RT	11	2.0E-2	8.5E-1
INTERPRO	Insulin-like growth factor binding protein, N-terminal	RT	15	5.6E-2	7.3E-1
UP_KEYWORDS	EGF-like domain	RT	17	7.0E-2	1.6E-1
UP_SEQ_FEATURE	domain:EGF-like 3; calcium-binding	RT	5	7.1E-2	9.9E-1
UP_SEQ_FEATURE	domain:EGF-like 2; calcium-binding	RT	6	9.4E-2	1.0E0
UP_SEQ_FEATURE	domain:EGF-like 5; calcium-binding	RT	3	2.0E-1	1.0E0
UP_SEQ_FEATURE	domain:EGF-like 6; calcium-binding	RT	3	2.0E-1	1.0E0
UP_SEQ_FEATURE	domain:EGF-like 2	RT	6	2.5E-1	1.0E0
UP_SEQ_FEATURE	domain:EGF-like 4	RT	3	2.9E-1	1.0E0
SMART	EGF_CA	RT	9	4.9E-1	9.9E-1
SMART	EGF	RT	11	6.3E-1	1.0E0
INTERPRO	EGF-like calcium-binding	RT	9	6.8E-1	1.0E0
INTERPRO	EGF-like calcium-binding, conserved site	RT	7	7.1E-1	1.0E0
INTERPRO	EGF-type aspartate/asparagine hydroxylation site	RT	7	7.4E-1	1.0E0
INTERPRO	EGF-like, conserved site	RT	12	8.3E-1	1.0E0
INTERPRO	Epidermal growth factor-like domain	RT	13	8.8E-1	1.0E0
Annotation Cluster 126	Enrichment Score: 0.61	G	Count	P_Value	Benjamini
SMART	DEFSN	RT	3	2.0E-1	8.6E-1
UP_KEYWORDS	Defensin	RT	4	2.3E-1	4.3E-1
INTERPRO	Beta defensin/Neutrophil defensin	RT	3	2.5E-1	9.9E-1
INTERPRO	Beta defensin type	RT	3	3.1E-1	1.0E0
Annotation Cluster 127	Enrichment Score: 0.61	G	Count	P_Value	Benjamini
INTERPRO	Septin	RT	4	9.8E-2	8.9E-1
PIR_SUPERFAMILY	septin	RT	4	2.9E-1	1.0E0
UP_SEQ_FEATURE	binding site:GTP	RT	5	3.5E-1	1.0E0
UP_SEQ_FEATURE	binding site:GTP; via amide nitrogen and carbonyl oxygen	RT	4	3.7E-1	1.0E0
Annotation Cluster 128	Enrichment Score: 0.6	G	Count	P_Value	Benjamini
SMART	SM01394	RT	5	6.8E-2	5.9E-1
INTERPRO	S100/CaBP-9k-type, calcium binding, subdomain	RT	5	1.1E-1	9.1E-1
INTERPRO	S100/Calbindin-D9k, conserved site	RT	3	4.6E-1	1.0E0
UP_SEQ_FEATURE	calcium-binding region:1; low affinity	RT	4	4.9E-1	1.0E0
UP_SEQ_FEATURE	calcium-binding region:2; high affinity	RT	4	5.5E-1	1.0E0
Annotation Cluster 129	Enrichment Score: 0.59	G	Count	P_Value	Benjamini
UP_KEYWORDS	Synapse	RT	19	2.4E-2	6.3E-2
UP_KEYWORDS	Postsynaptic cell membrane	RT	7	4.7E-1	7.1E-1
GOTERM_CC_DIRECT	postsynaptic density	RT	7	4.9E-1	9.2E-1
GOTERM_CC_DIRECT	postsynaptic membrane	RT	8	7.9E-1	9.9E-1
Annotation Cluster 130	Enrichment Score: 0.57	G	Count	P_Value	Benjamini

	Category	Term				Count	P_Value	Benjamini
	Annotation Cluster 1	Enrichment Score: 57.69	G			Count	P_Value	Benjamini
☐	INTERPRO	Apolipoprotein A1/A4/E	RT			3	5.5E-2	7.3E-1
☐	UP_SEQ_FEATURE	repeat:8	RT			8	9.3E-2	1.0E0
☐	UP_SEQ_FEATURE	repeat:6	RT			9	1.0E-1	1.0E0
☐	UP_SEQ_FEATURE	repeat:5	RT			9	1.0E-1	1.0E0
☐	GOTERM_BP_DIRECT	very-low-density lipoprotein particle remodeling	RT			3	1.3E-1	9.6E-1
☐	UP_SEQ_FEATURE	repeat:7	RT			8	1.5E-1	1.0E0
☐	UP_SEQ_FEATURE	repeat:9	RT			5	3.0E-1	1.0E0
☐	UP_SEQ_FEATURE	repeat:10	RT			5	3.5E-1	1.0E0
☐	UP_SEQ_FEATURE	repeat:12	RT			4	4.3E-1	1.0E0
☐	UP_SEQ_FEATURE	repeat:4	RT			11	4.8E-1	1.0E0
☐	UP_SEQ_FEATURE	repeat:11	RT			4	4.9E-1	1.0E0
☐	UP_SEQ_FEATURE	repeat:13	RT			3	6.7E-1	1.0E0
☐	UP_SEQ_FEATURE	repeat:3	RT			10	7.1E-1	1.0E0
☐	UP_SEQ_FEATURE	repeat:1	RT			12	7.7E-1	1.0E0
☐	UP_SEQ_FEATURE	repeat:2	RT			12	7.9E-1	1.0E0
	Annotation Cluster 131	Enrichment Score: 0.55	G			Count	P_Value	Benjamini
☐	INTERPRO	Cytochrome c domain	RT			3	3.5E-2	5.9E-1
☐	UP_SEQ_FEATURE	binding site:Heme (covalent)	RT			3	1.2E-1	1.0E0
☐	UP_KEYWORDS	Heme	RT			9	5.9E-1	8.2E-1
☐	GOTERM_MF_DIRECT	heme binding	RT			10	7.7E-1	1.0E0
☐	UP_SEQ_FEATURE	metal ion-binding site:Iron (heme axial ligand)	RT			5	9.8E-1	1.0E0
	Annotation Cluster 132	Enrichment Score: 0.54	G			Count	P_Value	Benjamini
☐	GOTERM_BP_DIRECT	actin filament bundle assembly	RT			6	8.1E-2	9.0E-1
☐	GOTERM_BP_DIRECT	actin filament network formation	RT			3	1.6E-1	9.7E-1
☐	INTERPRO	Actinin-type, actin-binding, conserved site	RT			4	1.9E-1	9.8E-1
☐	GOTERM_BP_DIRECT	actin crosslink formation	RT			3	2.3E-1	9.9E-1
☐	SMART	CH	RT			6	3.5E-1	9.7E-1
☐	UP_SEQ_FEATURE	domain:CH 1	RT			3	3.8E-1	1.0E0
☐	UP_SEQ_FEATURE	domain:CH 2	RT			3	3.8E-1	1.0E0
☐	INTERPRO	Calponin homology domain	RT			6	6.4E-1	1.0E0
☐	COG_ONTOLOGY	Cytoskeleton	RT			3	7.3E-1	9.8E-1
	Annotation Cluster 133	Enrichment Score: 0.54	G			Count	P_Value	Benjamini
☐	GOTERM_CC_DIRECT	eukaryotic translation initiation factor 3 complex	RT			4	9.8E-2	4.6E-1
☐	GOTERM_BP_DIRECT	regulation of translational initiation	RT			5	2.1E-1	9.9E-1
☐	GOTERM_BP_DIRECT	translational initiation	RT			5	2.7E-1	1.0E0
☐	UP_KEYWORDS	Initiation factor	RT			6	2.8E-1	5.0E-1
☐	GOTERM_CC_DIRECT	eukaryotic 48S preinitiation complex	RT			3	3.5E-1	8.4E-1
☐	GOTERM_CC_DIRECT	eukaryotic 43S preinitiation complex	RT			3	3.5E-1	8.4E-1
☐	GOTERM_MF_DIRECT	translation initiation factor activity	RT			6	4.8E-1	1.0E0
☐	GOTERM_BP_DIRECT	formation of translation preinitiation complex	RT			3	5.8E-1	1.0E0
	Annotation Cluster 134	Enrichment Score: 0.53	G			Count	P_Value	Benjamini
☐	INTERPRO	Aldo/keto reductase	RT			4	1.9E-1	9.8E-1
☐	UP_SEQ_FEATURE	site:Lowers pKa of active site Tyr	RT			3	2.0E-1	1.0E0
☐	INTERPRO	NADP-dependent oxidoreductase domain	RT			4	2.2E-1	9.9E-1
☐	INTERPRO	Aldo/keto reductase subgroup	RT			3	3.4E-1	1.0E0
☐	INTERPRO	Aldo/keto reductase, conserved site	RT			3	3.4E-1	1.0E0
☐	PIR_SUPERFAMILY	aldo-keto reductase	RT			3	6.7E-1	1.0E0
	Annotation Cluster 135	Enrichment Score: 0.52	G			Count	P_Value	Benjamini
☐	INTERPRO	Netrin domain	RT			4	2.6E-1	9.9E-1
☐	INTERPRO	Tissue inhibitor of metalloproteinases-like, OB-fold	RT			4	2.8E-1	1.0E0
☐	UP_SEQ_FEATURE	domain:NTR	RT			4	3.7E-1	1.0E0
	Annotation Cluster 136	Enrichment Score: 0.52	G			Count	P_Value	Benjamini

						Count	P_Value	Benjamini
	Annotation Cluster 1	Enrichment Score: 57.69	G			Count	P_Value	Benjamini
☐	UP_KEYWORDS	Centromere	RT			8	1.5E-1	3.1E-1
☐	GOTERM_CC_DIRECT	condensed chromosome kinetochore	RT			5	4.3E-1	8.9E-1
☐	UP_KEYWORDS	Kinetochore	RT			5	4.3E-1	6.7E-1
	Annotation Cluster 137	Enrichment Score: 0.52	G			Count	P_Value	Benjamini
☐	GOTERM_CC_DIRECT	protein phosphatase type 2A complex	RT			5	5.5E-2	3.3E-1
☐	GOTERM_CC_DIRECT	chromosome, centromeric region	RT			4	5.2E-1	9.3E-1
☐	KEGG_PATHWAY	Hepatitis C	RT			6	1.0E0	1.0E0
	Annotation Cluster 138	Enrichment Score: 0.5	G			Count	P_Value	Benjamini
☐	KEGG_PATHWAY	Endocrine and other factor-regulated calcium reabsorption	RT			11	8.2E-3	5.4E-2
☐	KEGG_PATHWAY	Oocyte meiosis	RT			21	1.1E-2	6.9E-2
☐	GOTERM_CC_DIRECT	heterotrimeric G-protein complex	RT			8	1.3E-2	1.1E-1
☐	KEGG_PATHWAY	Estrogen signaling pathway	RT			17	3.9E-2	2.0E-1
☐	UP_KEYWORDS	Sodium/potassium transport	RT			3	5.3E-2	1.3E-1
☐	KEGG_PATHWAY	Proximal tubule bicarbonate reclamation	RT			6	6.6E-2	2.8E-1
☐	GOTERM_BP_DIRECT	establishment or maintenance of transmembrane electrochemical gradient	RT			3	6.8E-2	8.7E-1
☐	KEGG_PATHWAY	Salivary secretion	RT			14	7.6E-2	3.1E-1
☐	KEGG_PATHWAY	Adrenergic signaling in cardiomyocytes	RT			20	9.6E-2	3.5E-1
☐	KEGG_PATHWAY	cGMP-PKG signaling pathway	RT			22	1.3E-1	4.2E-1
☐	KEGG_PATHWAY	Platelet activation	RT			18	1.5E-1	4.4E-1
☐	GOTERM_CC_DIRECT	sodium:potassium-exchanging ATPase complex	RT			3	1.5E-1	5.8E-1
☐	GOTERM_MF_DIRECT	sodium:potassium-exchanging ATPase activity	RT			3	1.6E-1	8.3E-1
☐	GOTERM_BP_DIRECT	sodium ion export from cell	RT			3	1.6E-1	9.7E-1
☐	KEGG_PATHWAY	Thyroid hormone synthesis	RT			11	1.6E-1	4.6E-1
☐	GOTERM_BP_DIRECT	cellular potassium ion homeostasis	RT			3	1.9E-1	9.9E-1
☐	KEGG_PATHWAY	Gastric acid secretion	RT			11	1.9E-1	5.0E-1
☐	UP_KEYWORDS	Potassium	RT			9	2.3E-1	4.3E-1
☐	KEGG_PATHWAY	Dopaminergic synapse	RT			17	2.3E-1	5.5E-1
☐	KEGG_PATHWAY	Mineral absorption	RT			7	2.9E-1	6.3E-1
☐	KEGG_PATHWAY	Vascular smooth muscle contraction	RT			15	3.3E-1	6.7E-1
☐	KEGG_PATHWAY	Long-term potentiation	RT			9	3.4E-1	6.8E-1
☐	GOTERM_BP_DIRECT	cellular sodium ion homeostasis	RT			3	3.7E-1	1.0E0
☐	KEGG_PATHWAY	Amphetamine addiction	RT			9	3.7E-1	7.2E-1
☐	KEGG_PATHWAY	Bile secretion	RT			9	3.9E-1	7.3E-1
☐	KEGG_PATHWAY	Carbohydrate digestion and absorption	RT			6	4.1E-1	7.4E-1
☐	KEGG_PATHWAY	Glutamatergic synapse	RT			13	4.9E-1	8.1E-1
☐	KEGG_PATHWAY	Renin secretion	RT			8	5.2E-1	8.3E-1
☐	KEGG_PATHWAY	Oxytocin signaling pathway	RT			16	5.2E-1	8.3E-1
☐	KEGG_PATHWAY	Dilated cardiomyopathy	RT			10	5.2E-1	8.3E-1
☐	KEGG_PATHWAY	cAMP signaling pathway	RT			21	5.5E-1	8.4E-1
☐	UP_KEYWORDS	Potassium transport	RT			6	5.6E-1	8.0E-1
☐	KEGG_PATHWAY	Aldosterone-regulated sodium reabsorption	RT			5	5.7E-1	8.4E-1
☐	KEGG_PATHWAY	Insulin secretion	RT			9	6.1E-1	8.6E-1
☐	GOTERM_BP_DIRECT	potassium ion import	RT			3	6.3E-1	1.0E0
☐	KEGG_PATHWAY	Protein digestion and absorption	RT			9	6.3E-1	8.7E-1
☐	KEGG_PATHWAY	GnRH signaling pathway	RT			9	6.6E-1	8.9E-1
☐	KEGG_PATHWAY	Circadian entrainment	RT			10	6.7E-1	8.9E-1
☐	KEGG_PATHWAY	Regulation of lipolysis in adipocytes	RT			6	6.7E-1	8.9E-1
☐	KEGG_PATHWAY	GABAergic synapse	RT			9	6.7E-1	8.9E-1
☐	KEGG_PATHWAY	Cholinergic synapse	RT			11	7.0E-1	9.0E-1
☐	KEGG_PATHWAY	Morphine addiction	RT			9	7.5E-1	9.1E-1
☐	KEGG_PATHWAY	Inflammatory mediator regulation of TRP channels	RT			10	7.5E-1	9.1E-1
☐	KEGG_PATHWAY	Cocaine addiction	RT			5	7.5E-1	9.1E-1

Annotation Cluster 1	Enrichment Score: 57.69	G		Count	P_Value	Benjamini
KEGG_PATHWAY	Ovarian steroidogenesis	RT		5	7.6E-1	9.2E-1
UP_KEYWORDS	Sodium transport	RT		4	8.3E-1	9.6E-1
KEGG_PATHWAY	Aldosterone synthesis and secretion	RT		7	8.3E-1	9.5E-1
KEGG_PATHWAY	Serotonergic synapse	RT		10	8.5E-1	9.5E-1
KEGG_PATHWAY	Chemokine signaling pathway	RT		16	8.6E-1	9.6E-1
UP_KEYWORDS	Sodium	RT		4	8.6E-1	9.7E-1
KEGG_PATHWAY	Progesterone-mediated oocyte maturation	RT		7	9.0E-1	9.7E-1
KEGG_PATHWAY	Thyroid hormone signaling pathway	RT		9	9.0E-1	9.7E-1
KEGG_PATHWAY	Retrograde endocannabinoid signaling	RT		8	9.2E-1	9.7E-1
KEGG_PATHWAY	Melanogenesis	RT		7	9.5E-1	9.9E-1
GOTERM_BP_DIRECT	potassium ion transmembrane transport	RT		3	9.5E-1	1.0E0
UP_KEYWORDS	Ion channel	RT		5	1.0E0	1.0E0
KEGG_PATHWAY	Olfactory transduction	RT		3	1.0E0	1.0E0
Annotation Cluster 139	Enrichment Score: 0.48	G		Count	P_Value	Benjamini
UP_SEQ_FEATURE	domain:VWFA	RT		7	1.7E-1	1.0E0
SMART	VWA	RT		6	4.6E-1	9.9E-1
INTERPRO	von Willebrand factor, type A	RT		9	4.7E-1	1.0E0
Annotation Cluster 140	Enrichment Score: 0.48	G		Count	P_Value	Benjamini
SMART	MORN	RT		3	2.5E-1	9.1E-1
INTERPRO	MORN motif	RT		3	3.4E-1	1.0E0
UP_SEQ_FEATURE	repeat:MORN 1	RT		3	3.8E-1	1.0E0
UP_SEQ_FEATURE	repeat:MORN 2	RT		3	3.8E-1	1.0E0
Annotation Cluster 141	Enrichment Score: 0.46	G		Count	P_Value	Benjamini
INTERPRO	Protein phosphatase 2C, manganese/magnesium aspartate binding site	RT		3	2.2E-1	9.9E-1
INTERPRO	Protein phosphatase 2C	RT		3	3.7E-1	1.0E0
SMART	PP2Cc	RT		3	3.8E-1	9.7E-1
INTERPRO	Protein phosphatase 2C (PP2C)-like	RT		3	4.6E-1	1.0E0
Annotation Cluster 142	Enrichment Score: 0.45	G		Count	P_Value	Benjamini
INTERPRO	Peptidase C12, ubiquitin carboxyl-terminal hydrolase 1	RT		3	3.5E-2	5.9E-1
GOTERM_BP_DIRECT	protein deubiquitination	RT		7	3.5E-1	1.0E0
GOTERM_MF_DIRECT	thiol-dependent ubiquitin-specific protease activity	RT		7	5.7E-1	1.0E0
INTERPRO	Peptidase C19, ubiquitin carboxyl-terminal hydrolase 2, conserved site	RT		3	8.8E-1	1.0E0
INTERPRO	Peptidase C19, ubiquitin carboxyl-terminal hydrolase 2	RT		3	9.1E-1	1.0E0
Annotation Cluster 143	Enrichment Score: 0.45	G		Count	P_Value	Benjamini
SMART	SM01391	RT		9	8.0E-2	6.3E-1
UP_KEYWORDS	Keratin	RT		8	9.6E-2	2.1E-1
INTERPRO	Type II keratin	RT		5	1.2E-1	9.3E-1
UP_KEYWORDS	Intermediate filament	RT		9	1.5E-1	3.1E-1
INTERPRO	Intermediate filament protein, conserved site	RT		8	1.6E-1	9.6E-1
INTERPRO	Intermediate filament protein	RT		9	2.2E-1	9.9E-1
UP_SEQ_FEATURE	site:Stutter	RT		7	3.3E-1	1.0E0
GOTERM_CC_DIRECT	intermediate filament	RT		7	3.3E-1	8.2E-1
INTERPRO	Keratin, type I	RT		4	4.2E-1	1.0E0
GOTERM_MF_DIRECT	structural molecule activity	RT		15	4.4E-1	9.9E-1
UP_SEQ_FEATURE	region of interest:Head	RT		9	4.9E-1	1.0E0
GOTERM_CC_DIRECT	keratin filament	RT		6	5.1E-1	9.3E-1
UP_SEQ_FEATURE	region of interest:Linker 12	RT		8	5.8E-1	1.0E0
UP_SEQ_FEATURE	region of interest:Coil 1B	RT		8	6.2E-1	1.0E0
UP_SEQ_FEATURE	region of interest:Coil 1A	RT		8	6.2E-1	1.0E0
UP_SEQ_FEATURE	region of interest:Linker 1	RT		8	6.2E-1	1.0E0
UP_SEQ_FEATURE	region of interest:Coil 2	RT		8	6.5E-1	1.0E0

							Count	P_Value	Benjamini
	Annotation Cluster 1	Enrichment Score: 57.69	G				Count	P_Value	Benjamini
☐	UP_SEQ_FEATURE	region of interest:Rod	RT				9	6.8E-1	1.0E0
☐	UP_SEQ_FEATURE	region of interest:Tail	RT				8	7.9E-1	1.0E0
☐	UP_SEQ_FEATURE	compositionally biased region:Gly-rich	RT				7	9.9E-1	1.0E0
	Annotation Cluster 144	Enrichment Score: 0.44	G				Count	P_Value	Benjamini
☐	UP_SEQ_FEATURE	domain:Sema	RT				3	1.2E-1	1.0E0
☐	GOTERM_BP_DIRECT	semaphorin-plexin signaling pathway	RT				5	1.3E-1	9.6E-1
☐	SMART	PSI	RT				4	5.2E-1	9.9E-1
☐	SMART	Sema	RT				3	6.0E-1	1.0E0
☐	INTERPRO	Plexin-like fold	RT				4	6.7E-1	1.0E0
☐	INTERPRO	Semaphorin/CD100 antigen	RT				3	6.9E-1	1.0E0
	Annotation Cluster 145	Enrichment Score: 0.41	G				Count	P_Value	Benjamini
☐	UP_KEYWORDS	Immunoglobulin domain	RT				19	5.4E-2	1.3E-1
☐	UP_SEQ_FEATURE	domain:Ig-like V-type 2	RT				5	7.1E-2	9.9E-1
☐	UP_SEQ_FEATURE	domain:Ig-like C2-type 1	RT				9	8.0E-2	1.0E0
☐	UP_SEQ_FEATURE	domain:Ig-like C2-type 2	RT				9	1.0E-1	1.0E0
☐	UP_SEQ_FEATURE	domain:Ig-like V-type 1	RT				5	1.1E-1	1.0E0
☐	UP_SEQ_FEATURE	domain:Ig-like C2-type 3	RT				4	4.9E-1	1.0E0
☐	INTERPRO	CD80-like, immunoglobulin C2-set	RT				3	6.7E-1	1.0E0
☐	GOTERM_BP_DIRECT	heterophilic cell-cell adhesion via plasma membrane cell adhesion molecules	RT				3	8.8E-1	1.0E0
☐	SMART	IGc2	RT				10	9.0E-1	1.0E0
☐	INTERPRO	Immunoglobulin subtype 2	RT				10	9.7E-1	1.0E0
☐	INTERPRO	Immunoglobulin V-set	RT				6	9.9E-1	1.0E0
☐	SMART	IG	RT				14	9.9E-1	1.0E0
☐	INTERPRO	Immunoglobulin subtype	RT				14	1.0E0	1.0E0
☐	INTERPRO	Immunoglobulin-like domain	RT				20	1.0E0	1.0E0
☐	INTERPRO	Immunoglobulin-like fold	RT				25	1.0E0	1.0E0
	Annotation Cluster 146	Enrichment Score: 0.4	G				Count	P_Value	Benjamini
☐	GOTERM_BP_DIRECT	positive regulation of GTPase activity	RT				17	2.3E-1	9.9E-1
☐	UP_KEYWORDS	GTPase activation	RT				7	2.8E-1	5.0E-1
☐	GOTERM_MF_DIRECT	GTPase activator activity	RT				8	1.0E0	1.0E0
	Annotation Cluster 147	Enrichment Score: 0.39	G				Count	P_Value	Benjamini
☐	GOTERM_BP_DIRECT	regulation of proteasomal ubiquitin-dependent protein catabolic process	RT				4	8.8E-2	9.1E-1
☐	SMART	UBA	RT				3	5.8E-1	9.9E-1
☐	INTERPRO	UBA-like	RT				5	6.4E-1	1.0E0
☐	INTERPRO	Ubiquitin-associated/translation elongation factor EF1B, N-terminal, eukaryote	RT				3	8.6E-1	1.0E0
	Annotation Cluster 148	Enrichment Score: 0.37	G				Count	P_Value	Benjamini
☐	UP_KEYWORDS	Peroxisome	RT				6	2.3E-1	4.4E-1
☐	GOTERM_CC_DIRECT	peroxisome	RT				8	3.1E-1	8.0E-1
☐	KEGG_PATHWAY	Peroxisome	RT				9	6.3E-1	8.7E-1
☐	UP_SEQ_FEATURE	short sequence motif:Microbody targeting signal	RT				4	7.0E-1	1.0E0
	Annotation Cluster 149	Enrichment Score: 0.34	G				Count	P_Value	Benjamini
☐	SMART	FA58C	RT				3	3.5E-1	9.7E-1
☐	INTERPRO	Coagulation factor 5/8 C-terminal type domain	RT				3	4.9E-1	1.0E0
☐	INTERPRO	Galactose-binding domain-like	RT				7	5.4E-1	1.0E0
	Annotation Cluster 150	Enrichment Score: 0.34	G				Count	P_Value	Benjamini
☐	UP_KEYWORDS	DNA repair	RT				13	2.6E-1	4.7E-1
☐	UP_KEYWORDS	DNA damage	RT				13	3.8E-1	6.1E-1
☐	GOTERM_BP_DIRECT	DNA repair	RT				5	9.9E-1	1.0E0
	Annotation Cluster 151	Enrichment Score: 0.33	G				Count	P_Value	Benjamini
☐	SMART	KU	RT				3	4.0E-1	9.8E-1
☐	INTERPRO	Proteinase inhibitor I2, Kunitz, conserved site	RT				3	4.9E-1	1.0E0
☐	INTERPRO	Proteinase inhibitor I2, Kunitz metazoa	RT				3	5.1E-1	1.0E0
	Annotation Cluster 152	Enrichment Score: 0.32	G				Count	P_Value	Benjamini

	Category	Term		Count	P_Value	Benjamini
	Annotation Cluster 1	Enrichment Score: 57.69	G	Count	P_Value	Benjamini
☐	INTERPRO	HAD-like domain	RT	9	2.8E-1	1.0E0
☐	SMART	SM00831	RT	3	3.0E-1	9.5E-1
☐	INTERPRO	Cation-transporting P-type ATPase, C-terminal	RT	3	3.1E-1	1.0E0
☐	INTERPRO	P-type ATPase, transmembrane domain	RT	3	3.4E-1	1.0E0
☐	INTERPRO	Cation-transporting P-type ATPase, N-terminal	RT	3	4.0E-1	1.0E0
☐	GOTERM_CC_DIRECT	T-tubule	RT	3	4.4E-1	9.0E-1
☐	INTERPRO	P-type ATPase, phosphorylation site	RT	3	7.6E-1	1.0E0
☐	INTERPRO	P-type ATPase, cytoplasmic domain N	RT	3	7.6E-1	1.0E0
☐	INTERPRO	Cation-transporting P-type ATPase	RT	3	7.9E-1	1.0E0
☐	INTERPRO	P-type ATPase, A domain	RT	3	7.9E-1	1.0E0
	Annotation Cluster 153	Enrichment Score: 0.31	G	Count	P_Value	Benjamini
☐	UP_KEYWORDS	Vision	RT	6	2.3E-1	4.4E-1
☐	GOTERM_CC_DIRECT	photoreceptor inner segment	RT	4	3.5E-1	8.4E-1
☐	GOTERM_BP_DIRECT	visual perception	RT	9	7.2E-1	1.0E0
☐	UP_KEYWORDS	Sensory transduction	RT	6	1.0E0	1.0E0
	Annotation Cluster 154	Enrichment Score: 0.29	G	Count	P_Value	Benjamini
☐	INTERPRO	Tetraspanin, conserved site	RT	4	2.2E-1	9.9E-1
☐	INTERPRO	Tetraspanin	RT	4	4.6E-1	1.0E0
☐	INTERPRO	Tetraspanin, EC2 domain	RT	4	4.6E-1	1.0E0
☐	INTERPRO	Tetraspanin/Peripherin	RT	4	5.0E-1	1.0E0
☐	PIR_SUPERFAMILY	tetraspanin	RT	4	8.2E-1	1.0E0
☐	GOTERM_BP_DIRECT	cell surface receptor signaling pathway	RT	7	1.0E0	1.0E0
	Annotation Cluster 155	Enrichment Score: 0.28	G	Count	P_Value	Benjamini
☐	UP_SEQ_FEATURE	domain:PDZ	RT	6	4.4E-1	1.0E0
☐	SMART	PDZ	RT	10	5.2E-1	9.9E-1
☐	INTERPRO	PDZ domain	RT	11	6.6E-1	1.0E0
	Annotation Cluster 156	Enrichment Score: 0.26	G	Count	P_Value	Benjamini
☐	UP_KEYWORDS	Wnt signaling pathway	RT	11	5.6E-2	1.3E-1
☐	KEGG_PATHWAY	Hedgehog signaling pathway	RT	6	1.0E-1	3.7E-1
☐	UP_SEQ_FEATURE	binding site:ATP	RT	29	3.1E-1	1.0E0
☐	UP_KEYWORDS	Kinase	RT	39	3.6E-1	6.0E-1
☐	GOTERM_BP_DIRECT	protein phosphorylation	RT	14	4.8E-1	1.0E0
☐	GOTERM_BP_DIRECT	peptidyl-serine phosphorylation	RT	8	8.9E-1	1.0E0
☐	UP_KEYWORDS	Serine/threonine-protein kinase	RT	13	9.2E-1	9.9E-1
☐	UP_SEQ_FEATURE	domain:Protein kinase	RT	16	9.2E-1	1.0E0
☐	GOTERM_MF_DIRECT	protein serine/threonine kinase activity	RT	13	9.8E-1	1.0E0
☐	SMART	S_TKc	RT	15	9.9E-1	1.0E0
☐	INTERPRO	Serine/threonine-protein kinase, active site	RT	13	1.0E0	1.0E0
☐	INTERPRO	Protein kinase, ATP binding site	RT	14	1.0E0	1.0E0
☐	INTERPRO	Protein kinase, catalytic domain	RT	17	1.0E0	1.0E0
☐	INTERPRO	Protein kinase-like domain	RT	17	1.0E0	1.0E0
	Annotation Cluster 157	Enrichment Score: 0.26	G	Count	P_Value	Benjamini
☐	UP_SEQ_FEATURE	repeat:1-1	RT	4	4.3E-1	1.0E0
☐	UP_SEQ_FEATURE	repeat:2-3	RT	3	4.6E-1	1.0E0
☐	UP_SEQ_FEATURE	repeat:1-2	RT	4	4.9E-1	1.0E0
☐	UP_SEQ_FEATURE	repeat:2-2	RT	3	7.2E-1	1.0E0
☐	UP_SEQ_FEATURE	repeat:2-1	RT	3	7.2E-1	1.0E0
	Annotation Cluster 158	Enrichment Score: 0.25	G	Count	P_Value	Benjamini
☐	SMART	H2B	RT	4	5.1E-1	9.9E-1
☐	GOTERM_CC_DIRECT	nuclear nucleosome	RT	5	5.4E-1	9.4E-1
☐	GOTERM_BP_DIRECT	nucleosome assembly	RT	11	5.7E-1	1.0E0
☐	INTERPRO	Histone H2B	RT	4	6.2E-1	1.0E0
	Annotation Cluster 159	Enrichment Score: 0.24	G	Count	P_Value	Benjamini

	Category	Term				Count	P_Value	Benjamini
	Annotation Cluster 1	Enrichment Score: 57.69	G			Count	P_Value	Benjamini
☐	SMART	SM01055	RT			3	1.0E-1	6.8E-1
☐	INTERPRO	Cadherin prodomain	RT			3	1.3E-1	9.4E-1
☐	UP_SEQ_FEATURE	domain:Cadherin 2	RT			4	5.5E-1	1.0E0
☐	UP_SEQ_FEATURE	domain:Cadherin 1	RT			4	5.5E-1	1.0E0
☐	UP_SEQ_FEATURE	domain:Cadherin 5	RT			3	7.7E-1	1.0E0
☐	UP_SEQ_FEATURE	domain:Cadherin 4	RT			3	8.0E-1	1.0E0
☐	UP_SEQ_FEATURE	domain:Cadherin 3	RT			3	8.0E-1	1.0E0
☐	INTERPRO	Cadherin-like	RT			5	8.8E-1	1.0E0
☐	SMART	CA	RT			4	8.9E-1	1.0E0
☐	INTERPRO	Cadherin	RT			4	9.4E-1	1.0E0
☐	INTERPRO	Cadherin conserved site	RT			3	9.8E-1	1.0E0
☐	GOTERM_BP_DIRECT	homophilic cell adhesion via plasma membrane adhesion molecules	RT			5	9.8E-1	1.0E0
	Annotation Cluster 160	Enrichment Score: 0.23	G			Count	P_Value	Benjamini
☐	UP_SEQ_FEATURE	repeat:HEAT 4	RT			3	5.4E-1	1.0E0
☐	UP_SEQ_FEATURE	repeat:HEAT 1	RT			3	6.1E-1	1.0E0
☐	UP_SEQ_FEATURE	repeat:HEAT 3	RT			3	6.1E-1	1.0E0
☐	UP_SEQ_FEATURE	repeat:HEAT 2	RT			3	6.1E-1	1.0E0
	Annotation Cluster 161	Enrichment Score: 0.22	G			Count	P_Value	Benjamini
☐	SMART	FBG	RT			3	4.3E-1	9.8E-1
☐	INTERPRO	Fibrinogen, alpha/beta/gamma chain, C-terminal globular, subdomain 2	RT			3	5.1E-1	1.0E0
☐	INTERPRO	Fibrinogen, alpha/beta/gamma chain, C-terminal globular, subdomain 1	RT			3	6.5E-1	1.0E0
☐	INTERPRO	Fibrinogen, alpha/beta/gamma chain, C-terminal globular domain	RT			3	6.9E-1	1.0E0
☐	UP_SEQ_FEATURE	domain:Fibrinogen C-terminal	RT			3	7.7E-1	1.0E0
	Annotation Cluster 162	Enrichment Score: 0.22	G			Count	P_Value	Benjamini
☐	GOTERM_BP_DIRECT	positive regulation of osteoblast differentiation	RT			6	3.9E-1	1.0E0
☐	GOTERM_BP_DIRECT	negative regulation of extrinsic apoptotic signaling pathway	RT			4	5.7E-1	1.0E0
☐	KEGG_PATHWAY	Signaling pathways regulating pluripotency of stem cells	RT			3	1.0E0	1.0E0
	Annotation Cluster 163	Enrichment Score: 0.21	G			Count	P_Value	Benjamini
☐	GOTERM_BP_DIRECT	xenophagy	RT			9	5.0E-1	1.0E0
☐	GOTERM_BP_DIRECT	positive regulation of defense response to virus by host	RT			10	5.1E-1	1.0E0
☐	GOTERM_BP_DIRECT	mitophagy in response to mitochondrial depolarization	RT			8	8.9E-1	1.0E0
	Annotation Cluster 164	Enrichment Score: 0.2	G			Count	P_Value	Benjamini
☐	INTERPRO	Low-density lipoprotein (LDL) receptor class A repeat	RT			5	5.2E-1	1.0E0
☐	SMART	LDLa	RT			4	5.7E-1	9.9E-1
☐	INTERPRO	Low-density lipoprotein (LDL) receptor class A, conserved site	RT			4	5.8E-1	1.0E0
☐	INTERPRO	Thrombospondin, type 1 repeat	RT			5	7.2E-1	1.0E0
☐	SMART	TSP1	RT			4	7.7E-1	1.0E0
	Annotation Cluster 165	Enrichment Score: 0.2	G			Count	P_Value	Benjamini
☐	SMART	ARM	RT			4	5.4E-1	9.9E-1
☐	UP_SEQ_FEATURE	repeat:ARM 2	RT			3	6.7E-1	1.0E0
☐	UP_SEQ_FEATURE	repeat:ARM 3	RT			3	6.7E-1	1.0E0
☐	INTERPRO	Armadillo	RT			4	6.9E-1	1.0E0
	Annotation Cluster 166	Enrichment Score: 0.18	G			Count	P_Value	Benjamini
☐	UP_KEYWORDS	TPR repeat	RT			8	1.2E-1	2.6E-1
☐	INTERPRO	Tetratricopeptide TPR-1	RT			3	4.9E-1	1.0E0
☐	INTERPRO	Tetratricopeptide-like helical	RT			16	5.9E-1	1.0E0
☐	UP_SEQ_FEATURE	repeat:TPR 3	RT			7	7.1E-1	1.0E0
☐	SMART	TPR	RT			7	8.0E-1	1.0E0
☐	UP_SEQ_FEATURE	repeat:TPR 1	RT			7	8.6E-1	1.0E0
☐	UP_SEQ_FEATURE	repeat:TPR 2	RT			7	8.6E-1	1.0E0
☐	UP_SEQ_FEATURE	repeat:TPR 5	RT			3	8.9E-1	1.0E0

	Category	Term		Count	P_Value	Benjamini
	Annotation Cluster 1	Enrichment Score: 57.69	G	Count	P_Value	Benjamini
☐	UP_SEQ_FEATURE	repeat:TPR 4	RT	3	9.1E-1	1.0E0
☐	INTERPRO	Tetratricopeptide repeat-containing domain	RT	7	9.1E-1	1.0E0
☐	INTERPRO	Tetratricopeptide repeat	RT	7	9.4E-1	1.0E0
	Annotation Cluster 167	Enrichment Score: 0.18	G	Count	P_Value	Benjamini
☐	SMART	TSPN	RT	3	3.5E-1	9.7E-1
☐	SMART	LamG	RT	3	7.2E-1	1.0E0
☐	INTERPRO	Laminin G domain	RT	4	7.7E-1	1.0E0
☐	INTERPRO	Concanavalin A-like lectin/glucanase, subgroup	RT	9	9.9E-1	1.0E0
	Annotation Cluster 168	Enrichment Score: 0.17	G	Count	P_Value	Benjamini
☐	UP_SEQ_FEATURE	domain:Fibronectin type-III 3	RT	3	4.6E-1	1.0E0
☐	UP_SEQ_FEATURE	domain:Fibronectin type-III 2	RT	4	5.5E-1	1.0E0
☐	UP_SEQ_FEATURE	domain:Fibronectin type-III 1	RT	4	5.5E-1	1.0E0
☐	SMART	FN3	RT	5	9.9E-1	1.0E0
☐	INTERPRO	Fibronectin, type III	RT	6	1.0E0	1.0E0
	Annotation Cluster 169	Enrichment Score: 0.14	G	Count	P_Value	Benjamini
☐	UP_SEQ_FEATURE	domain:Ig-like C1-type	RT	3	3.8E-1	1.0E0
☐	SMART	IGc1	RT	4	8.3E-1	1.0E0
☐	INTERPRO	Immunoglobulin C1-set	RT	4	9.1E-1	1.0E0
☐	INTERPRO	Immunoglobulin/major histocompatibility complex, conserved site	RT	3	9.3E-1	1.0E0
	Annotation Cluster 170	Enrichment Score: 0.13	G	Count	P_Value	Benjamini
☐	INTERPRO	Transforming growth factor-beta, N-terminal	RT	3	5.1E-1	1.0E0
☐	GOTERM_BP_DIRECT	positive regulation of pathway-restricted SMAD protein phosphorylation	RT	5	5.6E-1	1.0E0
☐	SMART	TGFB	RT	3	6.9E-1	1.0E0
☐	INTERPRO	Transforming growth factor-beta-related	RT	3	7.3E-1	1.0E0
☐	INTERPRO	Transforming growth factor beta, conserved site	RT	3	7.3E-1	1.0E0
☐	GOTERM_BP_DIRECT	SMAD protein signal transduction	RT	5	7.8E-1	1.0E0
☐	GOTERM_MF_DIRECT	transforming growth factor beta receptor binding	RT	3	7.8E-1	1.0E0
☐	INTERPRO	Transforming growth factor-beta, C-terminal	RT	3	8.0E-1	1.0E0
☐	GOTERM_MF_DIRECT	cytokine activity	RT	9	9.0E-1	1.0E0
☐	GOTERM_BP_DIRECT	regulation of MAPK cascade	RT	3	9.2E-1	1.0E0
☐	GOTERM_BP_DIRECT	cell development	RT	3	9.4E-1	1.0E0
	Annotation Cluster 171	Enrichment Score: 0.12	G	Count	P_Value	Benjamini
☐	GOTERM_CC_DIRECT	SNARE complex	RT	6	3.9E-1	8.7E-1
☐	GOTERM_MF_DIRECT	SNAP receptor activity	RT	3	8.5E-1	1.0E0
☐	KEGG_PATHWAY	SNARE interactions in vesicular transport	RT	3	8.8E-1	9.6E-1
☐	UP_SEQ_FEATURE	topological domain:Vesicular	RT	3	8.9E-1	1.0E0
☐	GOTERM_BP_DIRECT	vesicle fusion	RT	3	9.5E-1	1.0E0
	Annotation Cluster 172	Enrichment Score: 0.09	G	Count	P_Value	Benjamini
☐	UP_SEQ_FEATURE	metal ion-binding site:Calcium 2	RT	5	7.9E-1	1.0E0
☐	UP_SEQ_FEATURE	metal ion-binding site:Calcium 1	RT	4	8.1E-1	1.0E0
☐	UP_SEQ_FEATURE	metal ion-binding site:Calcium 2; via carbonyl oxygen	RT	4	8.4E-1	1.0E0
	Annotation Cluster 173	Enrichment Score: 0.07	G	Count	P_Value	Benjamini
☐	UP_KEYWORDS	WD repeat	RT	13	2.6E-1	4.8E-1
☐	INTERPRO	G-protein beta WD-40 repeat	RT	6	7.6E-1	1.0E0
☐	UP_SEQ_FEATURE	repeat:WD 3	RT	13	9.1E-1	1.0E0
☐	UP_SEQ_FEATURE	repeat:WD 4	RT	13	9.1E-1	1.0E0
☐	UP_SEQ_FEATURE	repeat:WD 5	RT	12	9.1E-1	1.0E0
☐	UP_SEQ_FEATURE	repeat:WD 1	RT	13	9.2E-1	1.0E0
☐	UP_SEQ_FEATURE	repeat:WD 2	RT	13	9.2E-1	1.0E0
☐	INTERPRO	WD40 repeat, conserved site	RT	9	9.4E-1	1.0E0
☐	UP_SEQ_FEATURE	repeat:WD 7	RT	6	9.4E-1	1.0E0
☐	SMART	WD40	RT	12	9.5E-1	1.0E0

Annotation Cluster 1	Enrichment Score: 57.69	G	Count	P_Value	Benjamini
UP_SEQ_FEATURE	repeat:WD 6	RT	8	9.6E-1	1.0E0
INTERPRO	WD40/YVTN repeat-like-containing domain	RT	18	9.6E-1	1.0E0
INTERPRO	WD40 repeat	RT	12	9.9E-1	1.0E0
INTERPRO	WD40-repeat-containing domain	RT	12	1.0E0	1.0E0
Annotation Cluster 174	Enrichment Score: 0.06	G	Count	P_Value	Benjamini
UP_SEQ_FEATURE	region of interest:S-adenosyl-L-methionine binding	RT	3	7.2E-1	1.0E0
UP_SEQ_FEATURE	binding site:S-adenosyl-L-methionine	RT	3	8.9E-1	1.0E0
UP_KEYWORDS	Methyltransferase	RT	6	9.2E-1	9.9E-1
UP_KEYWORDS	S-adenosyl-L-methionine	RT	5	9.5E-1	1.0E0
Annotation Cluster 175	Enrichment Score: 0.05	G	Count	P_Value	Benjamini
UP_KEYWORDS	Leucine-rich repeat	RT	13	6.3E-1	8.4E-1
UP_SEQ_FEATURE	repeat:LRR 2	RT	13	8.2E-1	1.0E0
UP_SEQ_FEATURE	repeat:LRR 1	RT	13	8.2E-1	1.0E0
UP_SEQ_FEATURE	repeat:LRR 3	RT	12	8.4E-1	1.0E0
UP_SEQ_FEATURE	repeat:LRR 4	RT	10	8.8E-1	1.0E0
UP_SEQ_FEATURE	repeat:LRR 5	RT	9	8.9E-1	1.0E0
UP_SEQ_FEATURE	repeat:LRR 7	RT	7	8.9E-1	1.0E0
UP_SEQ_FEATURE	repeat:LRR 6	RT	8	9.1E-1	1.0E0
UP_SEQ_FEATURE	repeat:LRR 9	RT	4	9.7E-1	1.0E0
UP_SEQ_FEATURE	repeat:LRR 8	RT	4	9.8E-1	1.0E0
UP_SEQ_FEATURE	repeat:LRR 10	RT	3	9.9E-1	1.0E0
INTERPRO	Leucine-rich repeat	RT	10	1.0E0	1.0E0
SMART	LRR_TYP	RT	4	1.0E0	1.0E0
INTERPRO	Leucine-rich repeat, typical subtype	RT	4	1.0E0	1.0E0
Annotation Cluster 176	Enrichment Score: 0.05	G	Count	P_Value	Benjamini
KEGG_PATHWAY	VEGF signaling pathway	RT	6	7.3E-1	9.1E-1
KEGG_PATHWAY	B cell receptor signaling pathway	RT	4	9.8E-1	1.0E0
KEGG_PATHWAY	Natural killer cell mediated cytotoxicity	RT	4	1.0E0	1.0E0
Annotation Cluster 177	Enrichment Score: 0.02	G	Count	P_Value	Benjamini
UP_KEYWORDS	Membrane	RT	435	7.7E-1	9.4E-1
UP_SEQ_FEATURE	topological domain:Cytoplasmic	RT	118	1.0E0	1.0E0
UP_SEQ_FEATURE	transmembrane region	RT	194	1.0E0	1.0E0
UP_KEYWORDS	Transmembrane helix	RT	212	1.0E0	1.0E0
GOTERM_CC_DIRECT	integral component of membrane	RT	188	1.0E0	1.0E0
UP_KEYWORDS	Transmembrane	RT	217	1.0E0	1.0E0
Annotation Cluster 178	Enrichment Score: 0.01	G	Count	P_Value	Benjamini
SMART	LIM	RT	3	9.5E-1	1.0E0
UP_KEYWORDS	LIM domain	RT	3	9.7E-1	1.0E0
INTERPRO	Zinc finger, LIM-type	RT	3	9.8E-1	1.0E0
Annotation Cluster 179	Enrichment Score: 0.01	G	Count	P_Value	Benjamini
UP_SEQ_FEATURE	domain:C2	RT	3	9.2E-1	1.0E0
SMART	C2	RT	3	1.0E0	1.0E0
INTERPRO	C2 calcium-dependent membrane targeting	RT	3	1.0E0	1.0E0
Annotation Cluster 180	Enrichment Score: 0.01	G	Count	P_Value	Benjamini
UP_KEYWORDS	Spliceosome	RT	3	9.6E-1	1.0E0
GOTERM_BP_DIRECT	RNA splicing	RT	3	9.8E-1	1.0E0
GOTERM_BP_DIRECT	mRNA processing	RT	4	9.9E-1	1.0E0
UP_KEYWORDS	mRNA splicing	RT	4	9.9E-1	1.0E0
UP_KEYWORDS	mRNA processing	RT	4	1.0E0	1.0E0
Annotation Cluster 181	Enrichment Score: 0	G	Count	P_Value	Benjamini
UP_SEQ_FEATURE	domain:SH3	RT	4	9.8E-1	1.0E0
UP_KEYWORDS	SH3 domain	RT	4	1.0E0	1.0E0
SMART	SH3	RT	5	1.0E0	1.0E0
INTERPRO	Src homology-3 domain	RT	5	1.0E0	1.0E0

	Category	Term		Count	P_Value	Benjamini
	Annotation Cluster 1	Enrichment Score: 57.69	G	Count	P_Value	Benjamini
	Annotation Cluster 182	Enrichment Score: 0	G	Count	P_Value	Benjamini
☐	UP_SEQ_FEATURE	repeat:ANK 4	RT	3	9.9E-1	1.0E0
☐	UP_SEQ_FEATURE	repeat:ANK 3	RT	4	9.9E-1	1.0E0
☐	UP_SEQ_FEATURE	repeat:ANK 1	RT	4	1.0E0	1.0E0
☐	UP_SEQ_FEATURE	repeat:ANK 2	RT	4	1.0E0	1.0E0
☐	SMART	ANK	RT	5	1.0E0	1.0E0
☐	UP_KEYWORDS	ANK repeat	RT	5	1.0E0	1.0E0
☐	INTERPRO	Ankyrin repeat	RT	5	1.0E0	1.0E0
☐	INTERPRO	Ankyrin repeat-containing domain	RT	5	1.0E0	1.0E0
	Annotation Cluster 183	Enrichment Score: 0	G	Count	P_Value	Benjamini
☐	SMART	RRM	RT	4	1.0E0	1.0E0
☐	INTERPRO	RNA recognition motif domain	RT	4	1.0E0	1.0E0
☐	INTERPRO	Nucleotide-binding, alpha-beta plait	RT	4	1.0E0	1.0E0
	Annotation Cluster 184	Enrichment Score: 0	G	Count	P_Value	Benjamini
☐	UP_KEYWORDS	Transcription regulation	RT	26	1.0E0	1.0E0
☐	UP_KEYWORDS	Transcription	RT	29	1.0E0	1.0E0
☐	GOTERM_BP_DIRECT	transcription, DNA-templated	RT	27	1.0E0	1.0E0
☐	GOTERM_BP_DIRECT	regulation of transcription, DNA-templated	RT	17	1.0E0	1.0E0
	Annotation Cluster 185	Enrichment Score: 0	G	Count	P_Value	Benjamini
☐	SMART	RING	RT	3	1.0E0	1.0E0
☐	INTERPRO	Zinc finger, RING-type	RT	3	1.0E0	1.0E0
☐	INTERPRO	Zinc finger, RING/FYVE/PHD-type	RT	4	1.0E0	1.0E0
	Annotation Cluster 186	Enrichment Score: 0	G	Count	P_Value	Benjamini
☐	KEGG_PATHWAY	Neuroactive ligand-receptor interaction	RT	5	1.0E0	1.0E0
☐	INTERPRO	G protein-coupled receptor, rhodopsin-like	RT	4	1.0E0	1.0E0
☐	UP_KEYWORDS	G-protein coupled receptor	RT	5	1.0E0	1.0E0
☐	INTERPRO	GPCR, rhodopsin-like, 7TM	RT	4	1.0E0	1.0E0

1227 terms were not clustered.
