# Supplementary material for: Protein signatures of seminal plasma from bulls with contrasting frozen-thawed sperm viability
Source: Sci Rep. 2020 Sep 4;10:14661. doi: 10.1038/s41598-020-71015-9 (PMC7474054; doi:10.1038/s41598-020-71015-9)
Supplement: Supplementary file 3 — Supplementary Table 3. [file 41598_2020_71015_MOESM3_ESM.pdf]

Protein Signatures of Seminal Plasma from Bulls with Contrasting Frozen-Thawed Sperm Viability

Fabio P. Gomes,

Robin Park,

Arabela G. Viana,

Carolina Fernandez-Costa,

Einko Topper,

Abdullah Kaya,

Erdogan Memili,

John R. Yates, III,

Arlindo A. Moura

Supplemental Table 3. Analyses of variance, parameter estimates and fit diagnostics of regression analyses carried out on SAS University Edition, using a stepwise approach. For the regression models (summarized in Table 3), the frozen-thawed sperm viability (freezability score) of bulls was set as the dependent variable and abundances of seminal plasma proteins with the highest VIP scores (as shown in Figure 2), as the independent variables. Q9BGI1L and P29392L: log-transformed abundances of peroxiredoxin-5 (accession # Q9BGI1) and spermadhesin-1 (accession # P29392), respectively. P81019 and P81019L: abundances and log-transformed abundances of BSP5 (accession # P81019).

**Data Set**	WORK.IMPORTPLAN4ANDLOG
**Dependent Variable**	FreezeScore
**Selection Method**	Stepwise
**Select Criterion**	SBC
**Stop Criterion**	SBC
**Effect Hierarchy Enforced**	None

**Number of Observations Read**	14
**Number of Observations Used**	14

Dimensions	
**Number of Effects**	4
**Number of Parameters**	4

Stepwise Selection Summary				
**Step**	**Effect Entered**	**Effect Removed**	**Number Effects In**	**SBC**
**0**	**Intercept**		1	50.0757
**1**	**Q9BGI1L**		2	42.0419
**2**	**P29392L**		3	40.5569
**3**	**P29392L*P81019L**		4	36.1779*
*** Optimal Value of Criterion**				

Selection stopped because all effects are in the final model.

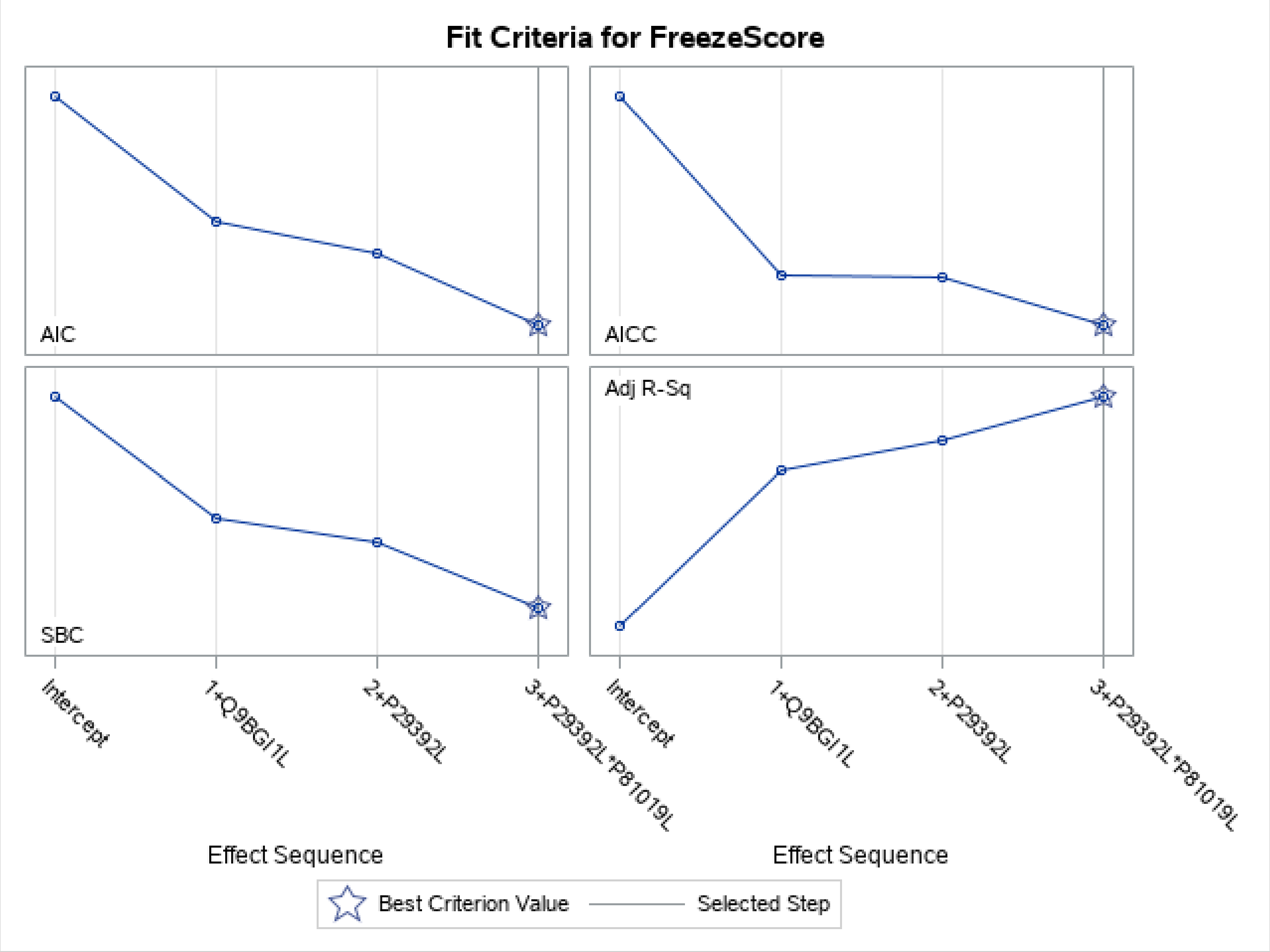

Selected Model

The selected model is the model at the last step (Step 3).

**Effects:**	Intercept P29392L Q9BGI1L P29392L*P81019L

Note: The p-values for parameters and effects are not adjusted for the fact that the terms in the model have been selected and so are generally liberal.

Analysis of Variance

Source	DF	Sum of Squares	Mean Square	F Value	Pr > F
Model	3	327.34750	109.11583	12.50	0.0010
Error	10	87.28465	8.72846		
Corrected Total	13	414.63214			

Root MSE	2.95440
Dependent Mean	3.53571
R-Square	0.7895
Adj R-Sq	0.7263
AIC	49.62164
AICC	57.12164
SBC	36.17787

Parameter Estimates

Parameter	DF	Estimate	Standard Error	t Value	Pr > |t|

Parameter Estimates					
**Parameter**	**DF**	**Estimate**	**Standard Error**	**t Value**	**Pr > |t|**
**Intercept**	1	221.301878	40.724327	5.43	0.0003
**P29392L**	1	-30.270225	8.902829	-3.40	0.0068
**Q9BGI1L**	1	-7.814084	3.166969	-2.47	0.0333
**P29392L*P81019L**	1	1.515310	0.593961	2.55	0.0288

Model: MODEL1
Dependent Variable: FreezeScore FreezeScore

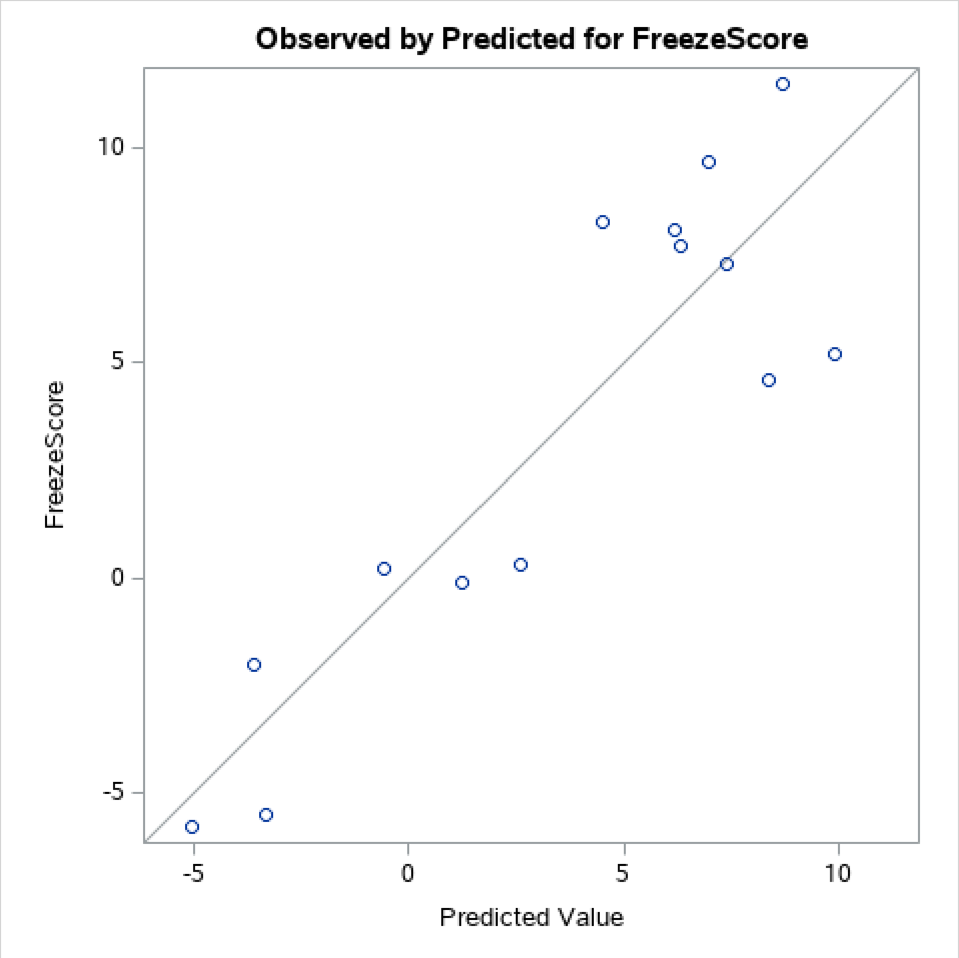

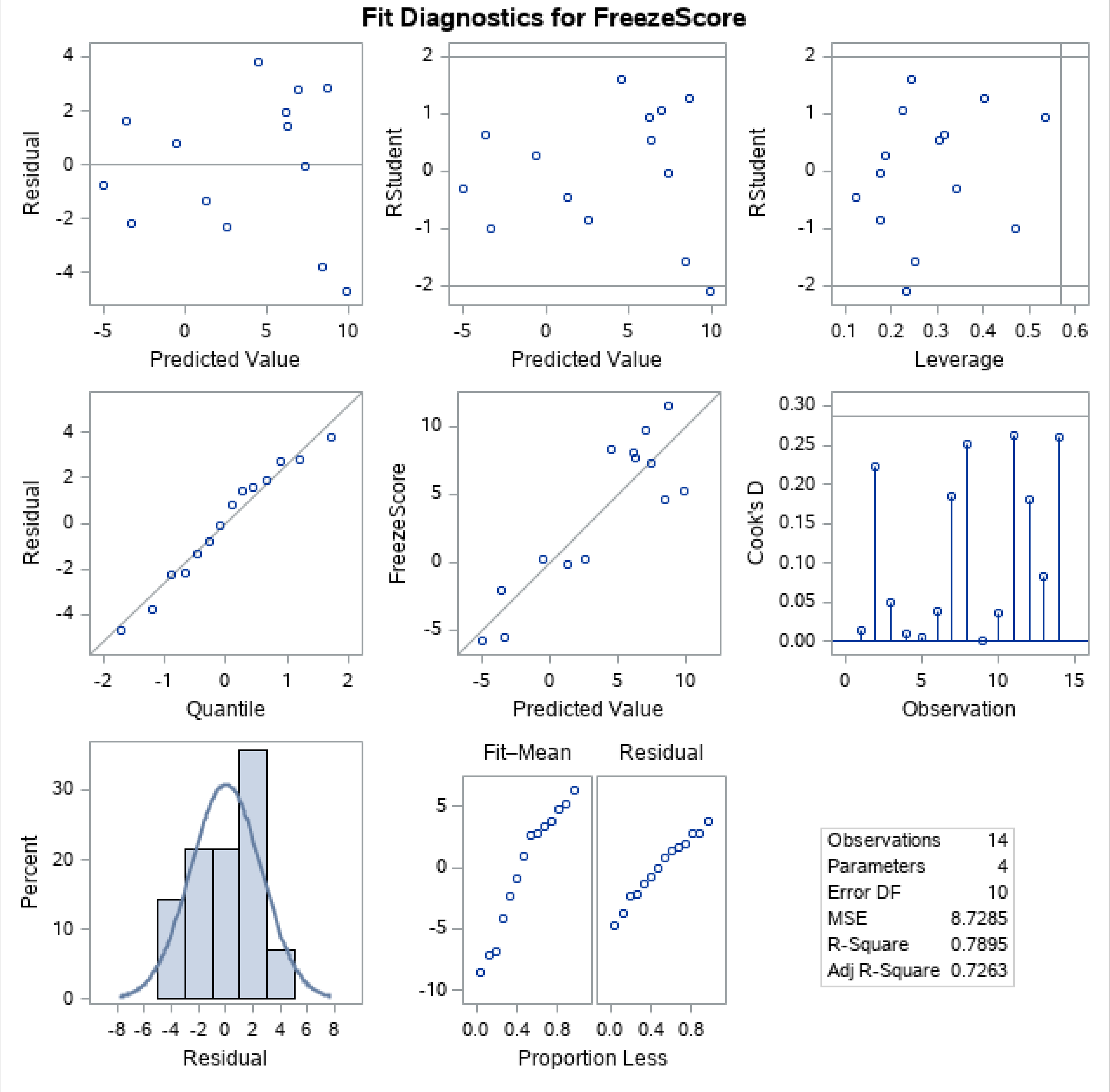

Observations	14
Parameters	4
Error DF	10
MSE	8.7285
R-Square	0.7895
Adj R-Square	0.7263

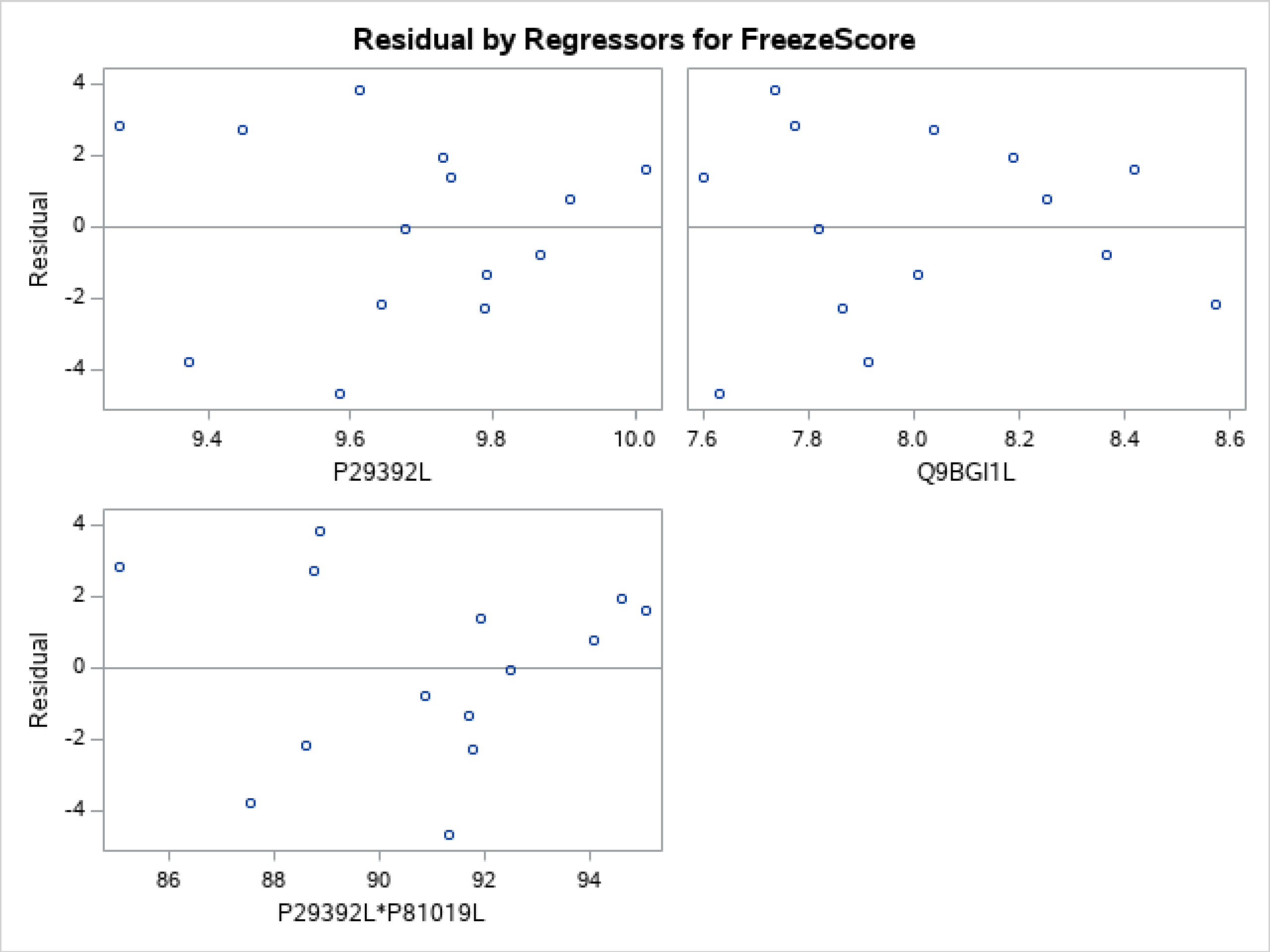
Residual by Regressors for FreezeScore
Residual
P29392L
Q9BGI1L
P29392L*P81019L

**Data Set**	WORK.IMPORTPLAN4ANDLOG
**Dependent Variable**	FreezeScore
**Selection Method**	Stepwise
**Select Criterion**	SBC
**Stop Criterion**	SBC
**Effect Hierarchy Enforced**	None

**Number of Observations Read**	14
**Number of Observations Used**	14

**Dimensions**	
**Number of Effects**	4
**Number of Parameters**	4

Stepwise Selection Summary

Step	Effect Entered	Effect Removed	Number Effects In	SBC
**0**	**Intercept**		1	50.0757
**1**	**Q9BGI1L**		2	42.0419
**2**	**P29392L**		3	40.5569
**3**	**P81019**		4	35.3358*

*** Optimal Value of Criterion**

Selection stopped because all effects are in the final model.

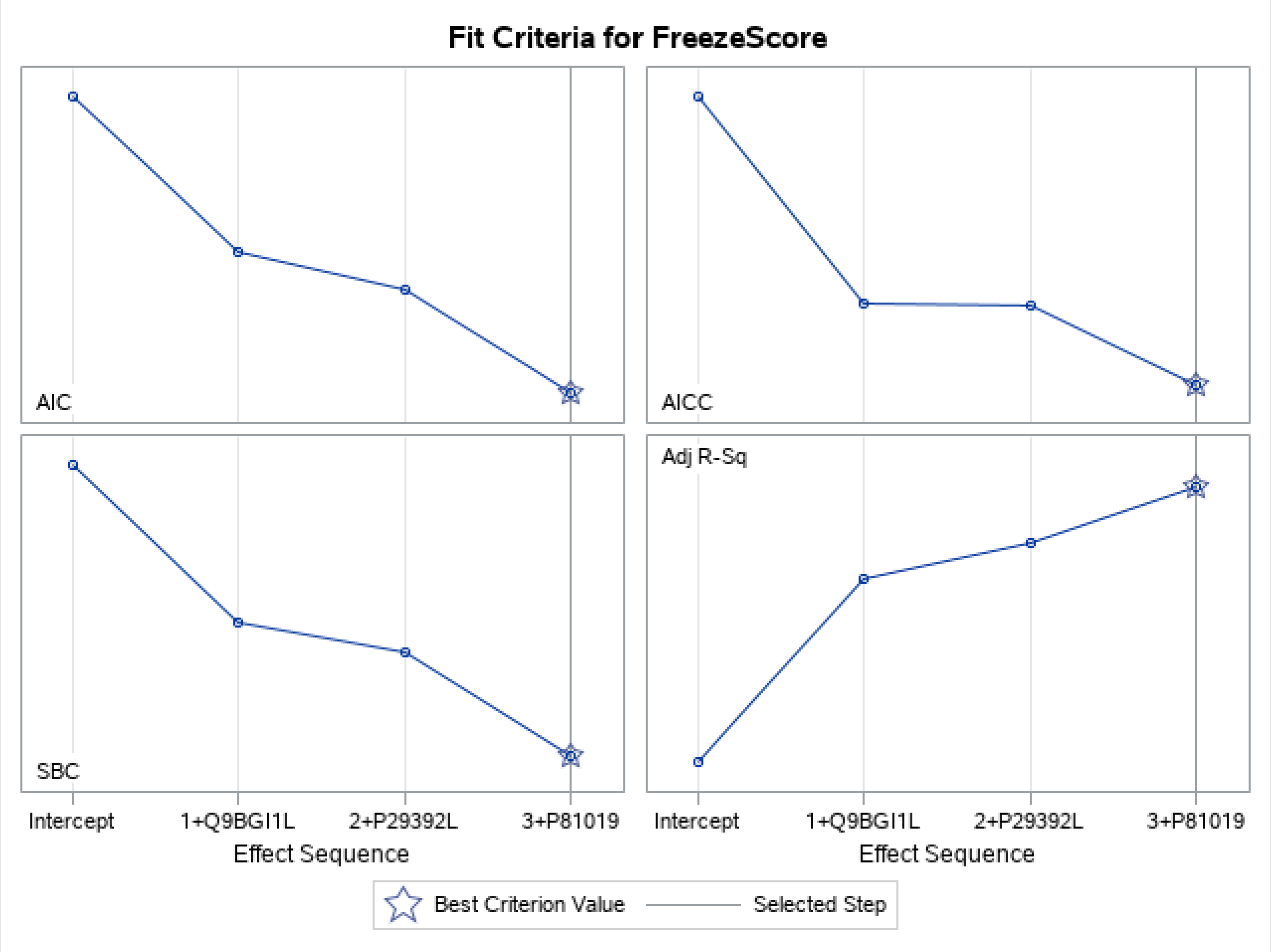

Selected Model

The selected model is the model at the last step (Step 3).

**Effects:**	Intercept Q9BGI1L P81019 P29392L

Note: The p-values for parameters and effects are not adjusted for the fact that the terms in the model have been selected and so are generally liberal.

Analysis of Variance					
**Source**	**DF**	**Sum of Squares**	**Mean Square**	**F Value**	**Pr > F**
**Model**	3	332.44248	110.81416	13.48	0.0008
**Error**	10	82.18967	8.21897		
**Corrected Total**	13	414.63214			

**Root MSE**	2.86687
**Dependent Mean**	3.53571
**R-Square**	0.8018
**Adj R-Sq**	0.7423
**AIC**	48.77961
**AICC**	56.27961
**SBC**	35.33584

Parameter Estimates					
**Parameter**	**DF**	**Estimate**	**Standard Error**	**t Value**	**Pr > |t|**

Parameter Estimates					
Parameter	DF	Estimate	Standard Error	t Value	Pr > |t|
Intercept	1	212.062253	38.441654	5.52	0.0003
Q9BGI1L	1	-8.614414	2.993342	-2.88	0.0164
P81019	1	2.2628731E-9	8.245296E-10	2.74	0.0207
P29392L	1	-15.033786	4.623548	-3.25	0.0087

Model: MODEL1
Dependent Variable: FreezeScore FreezeScore

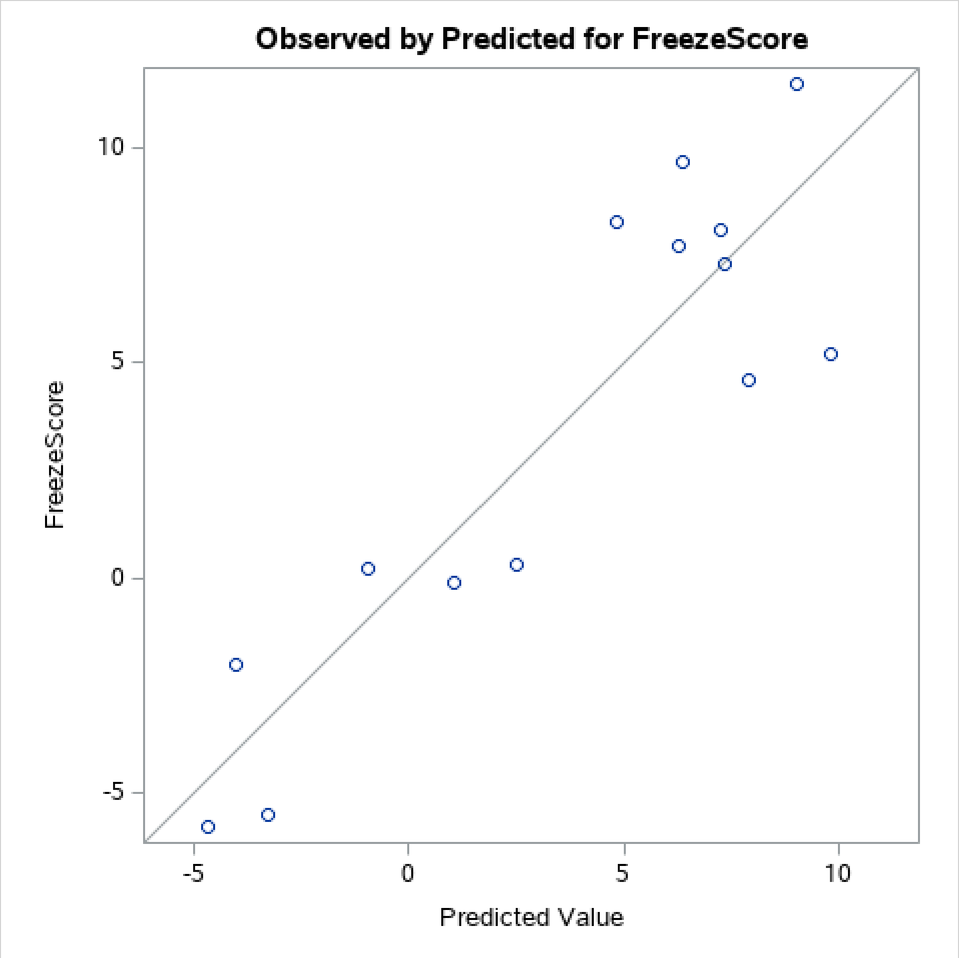

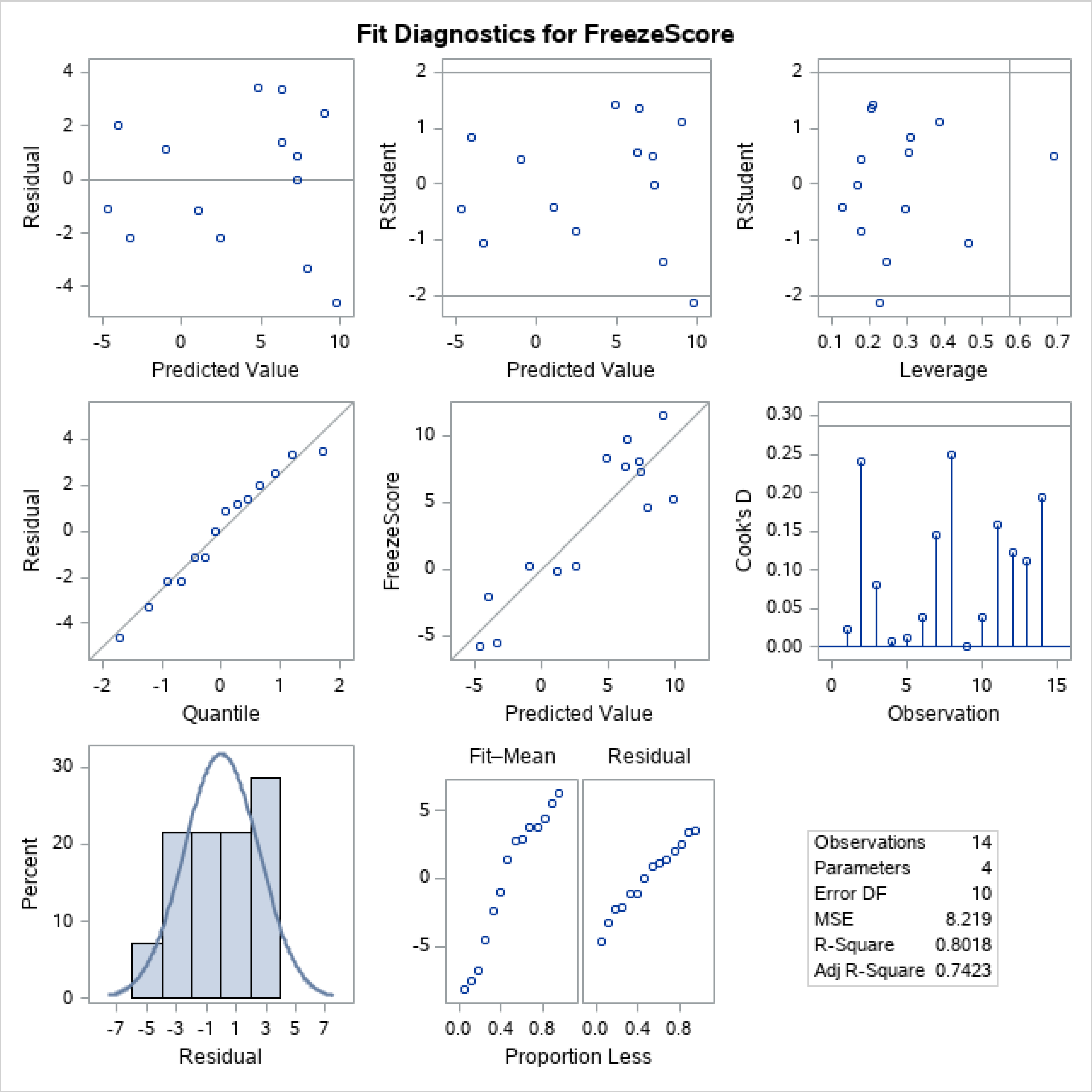
Fit Diagnostics for FreezeScore
Residual
Predicted Value
RStudent
Predicted Value
RStudent
Leverage
Residual
Quantile
FreezeScore
Predicted Value
Cook's D
Observation
Percent
Residual
Fit–Mean
Residual
Proportion Less
Observations 14
Parameters 4
Error DF 10
MSE 8.219
R-Square 0.8018
Adj R-Square 0.7423

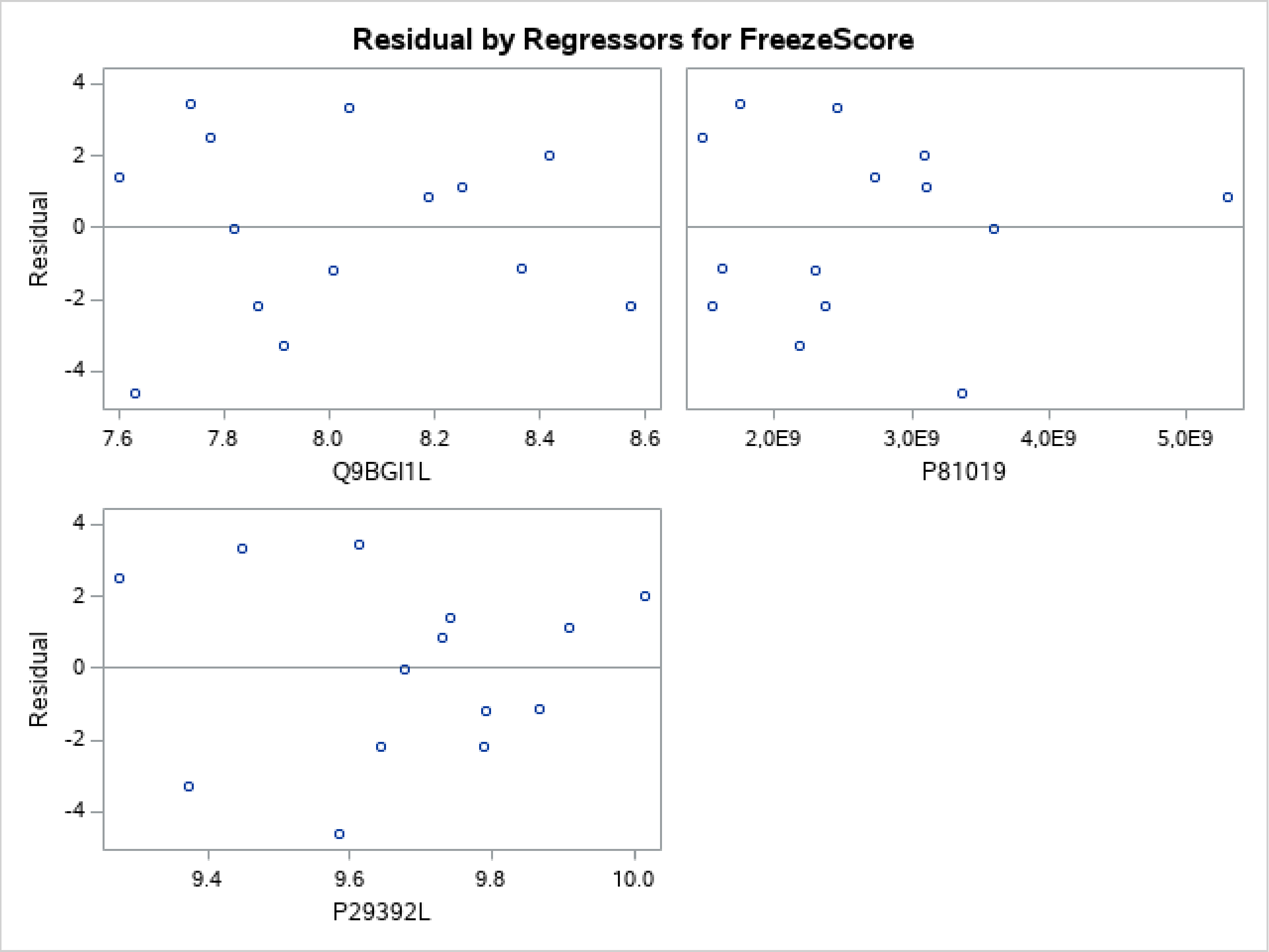
Residual by Regressors for FreezeScore
Residual
Q9BGI1L
P81019
P29392L
4
2
0
-2
-4
7.6
7.8
8.0
8.2
8.4
8.6
2,0E9
3,0E9
4,0E9
5,0E9
9.4
9.6
9.8
10.0
