# Supplementary material for: Protein signatures of seminal plasma from bulls with contrasting frozen-thawed sperm viability
Source: Sci Rep. 2020 Sep 4;10:14661. doi: 10.1038/s41598-020-71015-9 (PMC7474054; doi:10.1038/s41598-020-71015-9)
Supplement: Supplementary file 4 — Supplementary Table 4. [file 41598_2020_71015_MOESM4_ESM.pdf]

Protein Signatures of Seminal Plasma from Bulls with Contrasting Frozen-Thawed Sperm Viability

Fabio P. Gomes,

Robin Park,

Arabela G. Viana,

Carolina Fernandez-Costa,

Einko Topper,

Abdullah Kaya,

Erdogan Memili,

John R. Yates, III,

Arlindo A. Moura

Supplemental table 4. Pearson's correlations (and p-values) among abundances of seminal plasma proteins with the highest VIP scores (as shown in Figure 2). Analyses were carried out using SAS University Edition.

15 Variables:	P29392 P81019 Q2KJD0 P62157 P00669 Q3MHM5 Q3ZBU7 Q3SX14 Q2KJE5 P00829 Q9BGI1 Q32KL7 A0JNP2 Q3ZBD7 Q6B856

Pearson Correlation Coefficients, N = 14
Prob > |r| under H0: Rho=0

	P29392	P81019	Q2KJD0	P62157	P00669	Q3MHM5	Q3ZBU7	Q3SX14	Q2KJE5	P00829	Q9BGI1	Q32KL7	A0JNP2	Q3ZBD7	Q6B856
**P29392**<br>**P29392**	1.00000	0.23899<br>0.4106	0.77289<br>0.0012	0.60219<br>0.0227	-0.16414<br>0.5750	0.72287<br>0.0035	0.73463<br>0.0028	0.63904<br>0.0139	0.74157<br>0.0024	0.74314<br>0.0023	0.46332<br>0.0952	0.67380<br>0.0082	0.43593<br>0.1192	0.72511<br>0.0033	0.69303<br>0.0060
**P81019**<br>**P81019**	0.23899<br>0.4106	1.00000	0.04297<br>0.8840	-0.04901<br>0.8679	0.03190<br>0.9138	-0.02346<br>0.9365	0.00298<br>0.9919	-0.13836<br>0.6371	0.18480<br>0.5271	0.07069<br>0.8102	-0.11780<br>0.6884	0.09700<br>0.7415	-0.02440<br>0.9340	0.18156<br>0.5345	-0.04711<br>0.8729
**Q2KJD0**<br>**Q2KJD0**	0.77289<br>0.0012	0.04297<br>0.8840	1.00000	0.72665<br>0.0032	-0.49218<br>0.0738	0.98355<br><.0001	0.98804<br><.0001	0.58668<br>0.0274	0.85922<br><.0001	0.88444<br><.0001	0.44181<br>0.1137	0.73234<br>0.0029	0.66091<br>0.0101	0.91883<br><.0001	0.98049<br><.0001
**P62157**<br>**P62157**	0.60219<br>0.0227	-0.04901<br>0.8679	0.72665<br>0.0032	1.00000	-0.44254<br>0.1131	0.71793<br>0.0038	0.71351<br>0.0042	0.44072<br>0.1147	0.56115<br>0.0368	0.52838<br>0.0521	0.45569<br>0.1015	0.41244<br>0.1428	0.74801<br>0.0021	0.70152<br>0.0052	0.74426<br>0.0023
**P00669**<br>**P00669**	-0.16414<br>0.5750	0.03190<br>0.9138	-0.49218<br>0.0738	-0.44254<br>0.1131	1.00000	-0.49169<br>0.0741	-0.48041<br>0.0821	-0.26473<br>0.3604	-0.38947<br>0.1687	-0.37171<br>0.1907	-0.36065<br>0.2052	-0.25770<br>0.3737	-0.14580<br>0.6189	-0.40225<br>0.1539	-0.51001<br>0.0624
**Q3MHM5**<br>**Q3MHM5**	0.72287<br>0.0035	-0.02346<br>0.9365	0.98355<br><.0001	0.71793<br>0.0038	-0.49169<br>0.0741	1.00000	0.99896<br><.0001	0.52390<br>0.0545	0.86974<br><.0001	0.89685<br><.0001	0.47167<br>0.0886	0.74676<br>0.0022	0.68982<br>0.0063	0.92962<br><.0001	0.99529<br><.0001
**Q3ZBU7**<br>**Q3ZBU7**	0.73463<br>0.0028	0.00298<br>0.9919	0.98804<br><.0001	0.71351<br>0.0042	-0.48041<br>0.0821	0.99896<br><.0001	1.00000	0.52257<br>0.0552	0.87441<br><.0001	0.89458<br><.0001	0.44308<br>0.1126	0.74021<br>0.0025	0.69489<br>0.0058	0.93012<br><.0001	0.99432<br><.0001
**Q3SX14**<br>**Q3SX14**	0.63904<br>0.0139	-0.13836<br>0.6371	0.58668<br>0.0274	0.44072<br>0.1147	-0.26473<br>0.3604	0.52390<br>0.0545	0.52257<br>0.0552	1.00000	0.26920<br>0.3520	0.44828<br>0.1079	0.51048<br>0.0622	0.33433<br>0.2427	0.13388<br>0.6482	0.39601<br>0.1610	0.50957<br>0.0627
**Q2KJE5**<br>**Q2KJE5**	0.74157<br>0.0024	0.18480<br>0.5271	0.85922<br><.0001	0.56115<br>0.0368	-0.38947<br>0.1687	0.86974<br><.0001	0.87441<br><.0001	0.26920<br>0.3520	1.00000	0.94917<br><.0001	0.41062<br>0.1447	0.86997<br><.0001	0.49213<br>0.0738	0.87043<br><.0001	0.83923<br>0.0002
**P00829**<br>**P00829**	0.74314<br>0.0023	0.07069<br>0.8102	0.88444<br><.0001	0.52838<br>0.0521	-0.37171<br>0.1907	0.89685<br><.0001	0.89458<br><.0001	0.44828<br>0.1079	0.94917<br><.0001	1.00000	0.59175<br>0.0258	0.94500<br><.0001	0.42896<br>0.1259	0.89427<br><.0001	0.85850<br><.0001
**Q9BGI1**<br>**Q9BGI1**	0.46332<br>0.0952	-0.11780<br>0.6884	0.44181<br>0.1137	0.45569<br>0.1015	-0.36065<br>0.2052	0.47167<br>0.0886	0.44308<br>0.1126	0.51048<br>0.0622	0.41062<br>0.1447	0.59175<br>0.0258	1.00000	0.67418<br>0.0082	0.07021<br>0.8115	0.52308<br>0.0549	0.43863<br>0.1167
**Q32KL7**<br>**Q32KL7**	0.67380<br>0.0082	0.09700<br>0.7415	0.73234<br>0.0029	0.41244<br>0.1428	-0.25770<br>0.3737	0.74676<br>0.0022	0.74021<br>0.0025	0.33433<br>0.2427	0.86997<br><.0001	0.94500<br><.0001	0.67418<br>0.0082	1.00000	0.29438<br>0.3070	0.82830<br>0.0003	0.69848<br>0.0055
**A0JNP2**<br>**A0JNP2**	0.43593<br>0.1192	-0.02440<br>0.9340	0.66091<br>0.0101	0.74801<br>0.0021	-0.14580<br>0.6189	0.68982<br>0.0063	0.69489<br>0.0058	0.13388<br>0.6482	0.49213<br>0.0738	0.42896<br>0.1259	0.07021<br>0.8115	0.29438<br>0.3070	1.00000	0.72182<br>0.0036	0.72131<br>0.0036
**Q3ZBD7**<br>**Q3ZBD7**	0.72511<br>0.0033	0.18156<br>0.5345	0.91883<br><.0001	0.70152<br>0.0052	-0.40225<br>0.1539	0.92962<br><.0001	0.93012<br><.0001	0.39601<br>0.1610	0.87043<br><.0001	0.89427<br><.0001	0.52308<br>0.0549	0.82830<br>0.0003	0.72182<br>0.0036	1.00000	0.91329<br><.0001
**Q6B856**<br>**Q6B856**	0.69303<br>0.0060	-0.04711<br>0.8729	0.98049<br><.0001	0.74426<br>0.0023	-0.51001<br>0.0624	0.99529<br><.0001	0.99432<br><.0001	0.50957<br>0.0627	0.83923<br>0.0002	0.85850<br><.0001	0.43863<br>0.1167	0.69848<br>0.0055	0.72131<br>0.0036	0.91329<br><.0001	1.00000
